# Azides and Porphyrinoids: Synthetic Approaches and Applications. Part 1—Azides, Porphyrins and Corroles

**DOI:** 10.3390/molecules25071662

**Published:** 2020-04-03

**Authors:** Ana R. L. Araújo, Augusto C. Tomé, Carla I. M. Santos, Maria A. F. Faustino, Maria G. P. M. S. Neves, Mário M. Q. Simões, Nuno M. M. Moura, Sultan T. Abu-Orabi, José A. S. Cavaleiro

**Affiliations:** 1 LAQV-REQUIMTE, Department of Chemistry, University of Aveiro, 3810-193 Aveiro, Portugal; anararaujo@ua.pt (A.R.L.A.); actome@ua.pt (A.C.T.); cims@ua.pt (C.I.M.S.); faustino@ua.pt (M.A.F.F.); gneves@ua.pt (M.G.P.M.S.N.); msimoes@ua.pt (M.M.Q.S.); 2 CQE, Centro de Química Estrutural and IN-Institute of Nanoscience and Nanotechnology of Instituto Superior Técnico, Av. Rovisco Pais, 1049-001 Lisbon, Portugal; 3 Chemistry Department, Yarmouk University, Irbid 211-63, Jordan; dr.abuorabi@gmail.com

**Keywords:** Azides, Porphyrinoids, Porphyrins, Corroles, Click Chemistry, Cycloadditions, Photodynamic Therapy, Microorganisms Photoinactivation, Supramolecular assembly, Catalysis

## Abstract

Azides and porphyrinoids (such as porphyrin and corrole macrocycles) can give rise to new derivatives with significant biological properties and as new materials’ components. Significant synthetic approaches have been studied. A wide range of products (e.g., microporous organic networks, rotaxane and dendritic motifs, dendrimers as liquid crystals, as blood substitutes for transfusions and many others) can now be available and used for several medicinal and industrial purposes.

## 1. Introduction

Azide derivatives have been used in synthetic methodologies leading to a wide range of acyclic and cyclic nitrogen compounds. Such a “chemical avenue” started near the end of the 19th century. Phenyl azide was discovered by Griess in 1864 by diazotization of phenyl hydrazine with nitrous acid [1] and in 1890, Curtius, using hydrazine and nitrous acid, discovered hydrazoic acid under an identical diazotization approach [2]. At that time, sodium azide and potassium azide were also obtained from hydrazine and an alkyl nitrite respectively in NaOH or KOH [3].

Nature is really impressive with this use of such small molecules in methodologies that lead to a wide range of compounds, some of them with highly important applications. Such interest and development by scientists took place mainly after the 1950s. Since then, many publications have been taking place, particularly related with the synthesis of several organic azide derivatives and the study of their potential applications (e.g., vaccine preservatives, pesticides and AIDS treatments). A wide range of publications are available for azide synthesis and azide derivatizations [4,5].

Some products of such derivatization methods have demonstrated significant properties and applications. Zidovudin (AZT), an azide derivative displaying anti-AIDS application, is an example [6]. Other azide derivatives are involved in the synthesis of Paclitaxel (Taxol), an anticancer drug and Oseltamivir (Tamiflu), an anti-influenza A/B drug [7,8].

As already mentioned, a significant number of publications on the synthesis and reactions of azides have been put forward, mainly in the recent decades [9]. However, the cycloaddition reactions with azides is a topic that deserves to be highlighted in this introduction. This is due to the fact that depending on the dipolarophiles being used, several biologically active 1,2,3-triazole derivatives can be obtained by 1,3-dipolar cycloadditions [10,11]. In particular, when an organic azide and an alk-1-yne are being used there is the formation of 1,4- and 1,5-disubstituted 1,2,3-triazole isomers.

Such a method was further improved independently by the groups of Meldal [12], Fokin and Sharpless [13] by developing the Cu(I)-catalyzed azide-alkyne cycloaddition (CuAAC) reactions. This new procedure involving Cu(I) species in reactions of azides with alk-1-ynes originates selectively 1,4-disubstituted 1,2,3-triazoles in very high yields and purities. Later, Fokin and Sharpless reported that the ruthenium(II)-catalyzed azide-alkyne cycloaddition (RuAAC) reaction affords exclusively the 1,5-disubstituted 1,2,3-triazoles [14]. These metal-catalyzed cycloadditions are usually mentioned as click reactions. A recent publication describes the synthesis of “modular click chemistry libraries” [15].

This review will consider the scientific information published in recent years, involving chemical transformations not only of porphyrinoid and azide species or azide-porphyrinoid derivatives and their applications but also the potential biological products obtained from them. Porphyrinoid macrocycles are known to be of great significance to life. Natural porphyrins and related compounds play vital functions (e.g., respiration, photosynthesis, drug detoxification) and others can be used in very important medicinal applications [16]. Certain formulations have been already approved for the diagnosis of neoplastic situations and in the photodynamic therapy of certain cancer types and in the age-related macular degeneration. The photoinactivation of antibiotic resistant bacteria and viruses is also an important application coming out in the present times. Under such a line of work it can be stated that transformations involving both porphyrinoids with azides or azide-substituted porphyrinoids (porphyrin and corrole types) can lead to new derivatives that might become of great significance as new drugs, as pesticides, and even as catalysts in a wide range of reactions and in the synthesis of certain materials. Newly obtained products include the coupling of species (porphyrinoid-type and other heterocycles), which are themselves biologically active. The biological properties of the new derivatives might be increased and in such case that would presumably be due to any synergistic effect involving the coupled units. A wide variety of information about the azides/porphyrinoids chemical transformations and properties is available, in reviews and books/book chapters published until five years ago. This review is then focused on the scientific information that became available, mainly since 2015. Part one of this review is mainly focused on azides, porphyrins and corroles. Other macrocycles like phthalocyanines, subphthalocyanines and porphyrazines, and their transformations involving azides, are available in part two of the review, also published in this *Molecules* special issue [17].

## 2. Porphyrins

Porphyrins are a group of widely studied tetrapyrrolic macrocycles. This is not only due to their natural biological functions but also to the applications man can get from such a type of macrocycle. Synthetic methodologies based mainly on the azide chemical behavior leading to new porphyrin derivatives and their potential applications will be considered in this review.

### 2.1. Porphyrins Bearing Alkyne Groups in CuAAC Reactions

#### 2.1.1. Modification at Peripheral Substituents

**Scheme 1:**
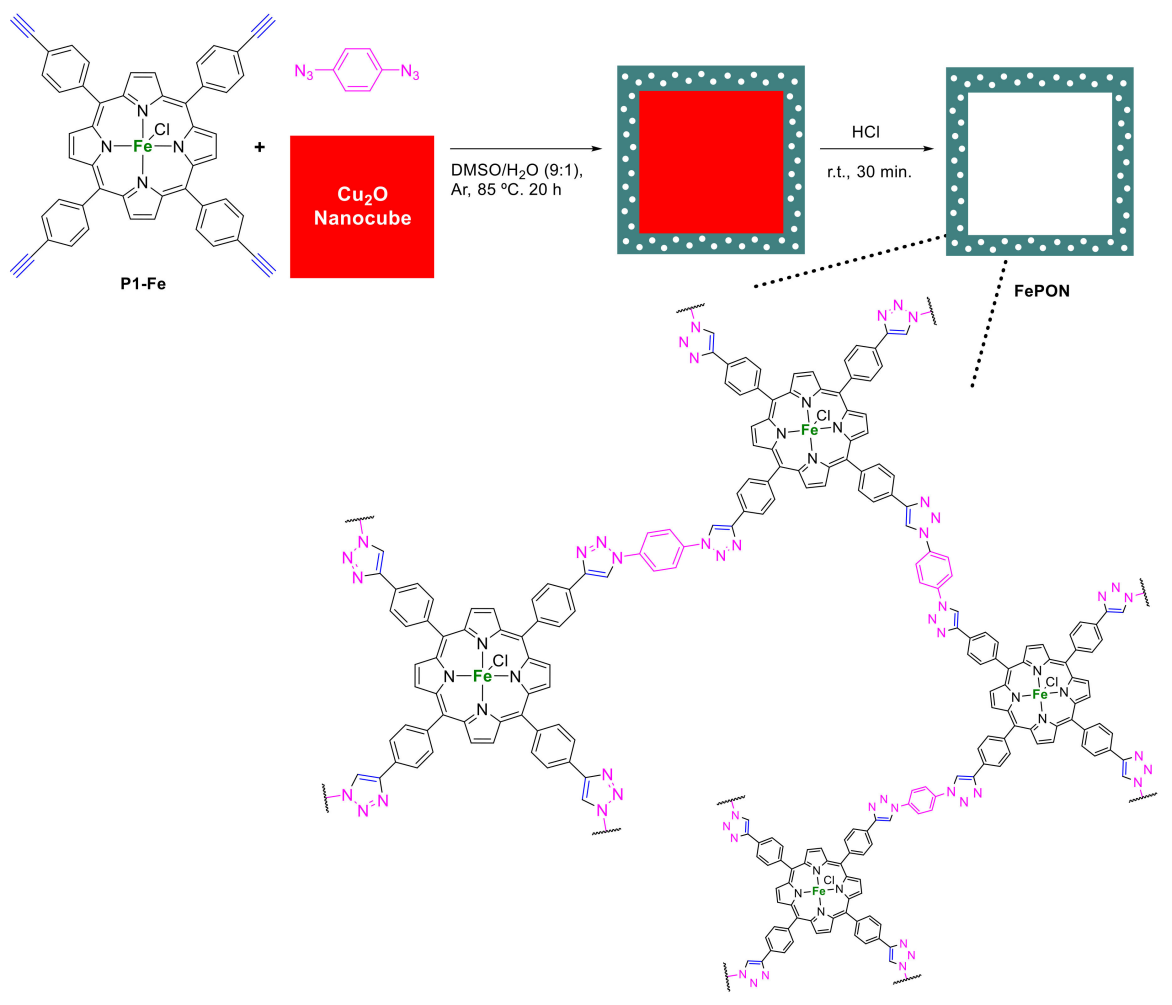
Schematic preparation of microporous porphyrin-based organic networks (**FePON**).

The iron(III) complex of 5,10,15,20-tetrakis(4-ethynylphenyl)porphyrin (**P1-Fe**) was used by Son and co-workers to prepare hollow microporous porphyrinic-based organic networks [18]. The authors used Cu_2_O nanocubes as templates and networking catalysts, and a click-chemistry approach. This reaction involved the addition of **P1-Fe** and 1,4-diazidobenzene to a suspension of Cu_2_O nanocubes in a DMSO/H_2_O mixture. The reaction mixture was heated for 20 h at 85 °C leading to the formation of the microporous Fe(III) porphyrin network on the surface of the Cu_2_O nanocubes. The Cu_2_O core was removed by acid treatment and the Fe(III) porphyrin network linked by bis-triazolylbenzene bridges (Scheme 1) was obtained. The Fe(III) porphyrin-based organic network (**FePON**) prepared was explored as a catalyst for carbene insertion into N-H bonds; the obtained results revealed excellent catalytic activity, selectivity and recyclability [18].

**Scheme 2:**
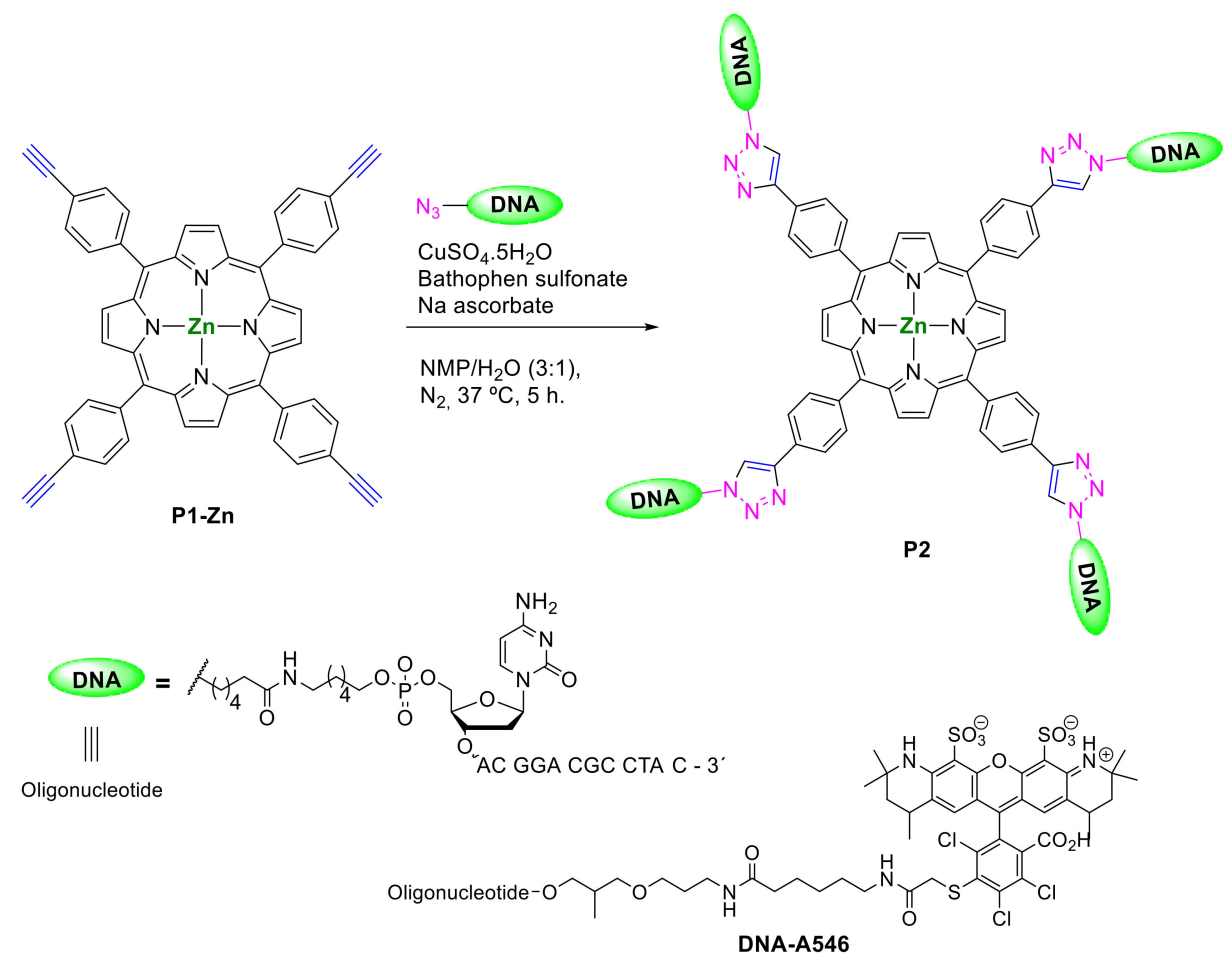
Synthesis of porphyrin derivatives bearing triazolyl-DNA oligonucleotides.

Wang and co-workers reported the reaction of the Zn(II) complex of 5,10,15,20-tetrakis(4-ethynylphenyl)porphyrin **P1-Zn** with 5´-azide terminated DNA oligonucleotide (Scheme 2) [19]. The reaction was performed under classical CuAAC conditions, in the presence of bathophenanthroline sulfonate as a Cu(I) stabilizing ligand, yielding a mixture of the corresponding porphyrin adducts containing one, two (*cis* and *trans*), three and four (**P2** in Scheme 2) triazolyl-DNA oligonucleotides.

**Scheme 3:**
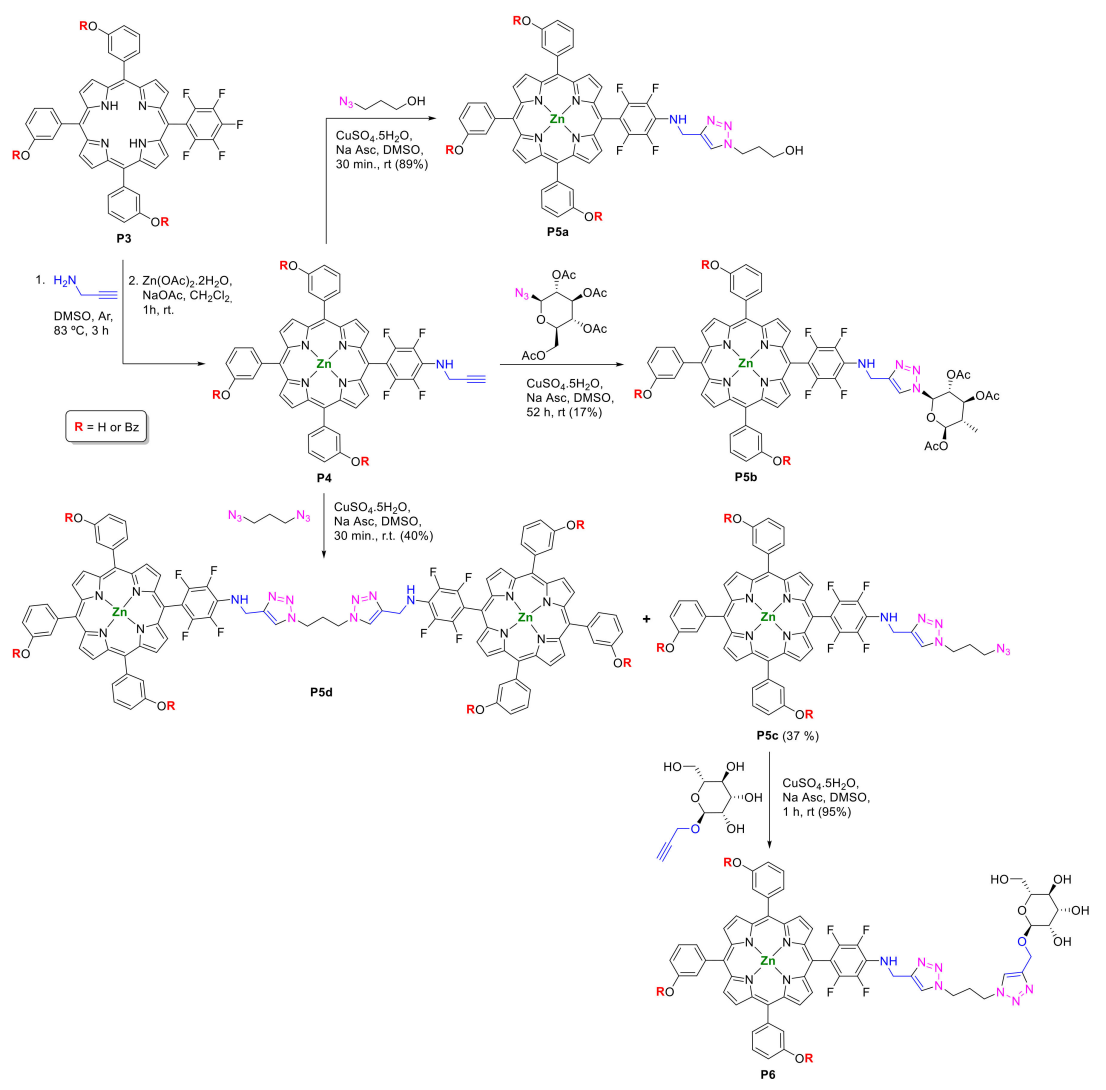
Porphyrin **P3** as useful template for CuAAC reactions.

The nucleophilic substitution of the *para* fluorine atom at the pentafluorophenyl ring in porphyrin **P3** by propargylamine afforded the alkynyl A_3_B porphyrin **P4** that showed to be a useful platform for further functionalization at the *meso*-substituents of the macrocycle via CuAAC reactions (Scheme 3). Using that strategy, Wiehe and co-workers prepared a set of porphyrin-triazole derivatives **P5a**–**d** containing several functions such as hydroxyl, mannosyl and azido groups, respectively **P5a**, **P5b** and **P5c**, and the porphyrinic dimeric species **P5d**. Macrocycle **P5c** was further modified with propargyl α-d-mannopyranoside affording derivative **P6** at a 95% yield (Scheme 3) [20].

Porphyrin conjugates **P5a**, **P5c**, and **P6** exhibited photocytotoxicity against human epidermoid carcinoma A-253 and squamous carcinoma CAL-27 cells [20].

**Scheme 4:**
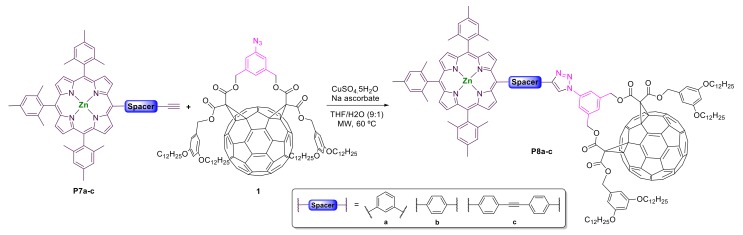
Synthesis of porphyrin–fullerene dyads **8a-c** by CuAAC reactions.

The access to porphyrin–fullerene dyads prepared by a CuAAC reaction using a fullerene functionalized with an azido group directly attached to the central bridging subunit was reported by Nierengarten and co-workers [21]. The reaction of the alkynylated Zn(II) porphyrins **P7a**–**c** bearing conjugated spacers (*m*-phenylene, *p*-phenylene, di-*p*-phenylene-ethynylene) with the azide moiety **1** was achieved under microwave irradiation (MW) at 60 °C and using CuSO_4_·5H_2_O and sodium ascorbate in a THF/H_2_O mixture (Scheme 4). Compounds **P8a**–**c** were obtained in moderate yields after 3 h (Scheme 4). The authors highlighted that when the reactions were performed at room temperature or under classical heating conditions a slow consumption of the starting material was observed and the desired compounds were obtained in low yields, this being due to the degradation of compound **1**. Compounds **8a**,**b** have shown relatively fast, efficient and long-lived photoinduced electron transfer (PET).

Other related synthetic approaches involving the preparation of porphyrin-fullerene dyads via CuAAC have been extensively reviewed by Hanh and Nierengarten [22] and Coutsolelos and co-workers [23], and will not be dealt with here.

**Scheme 5:**
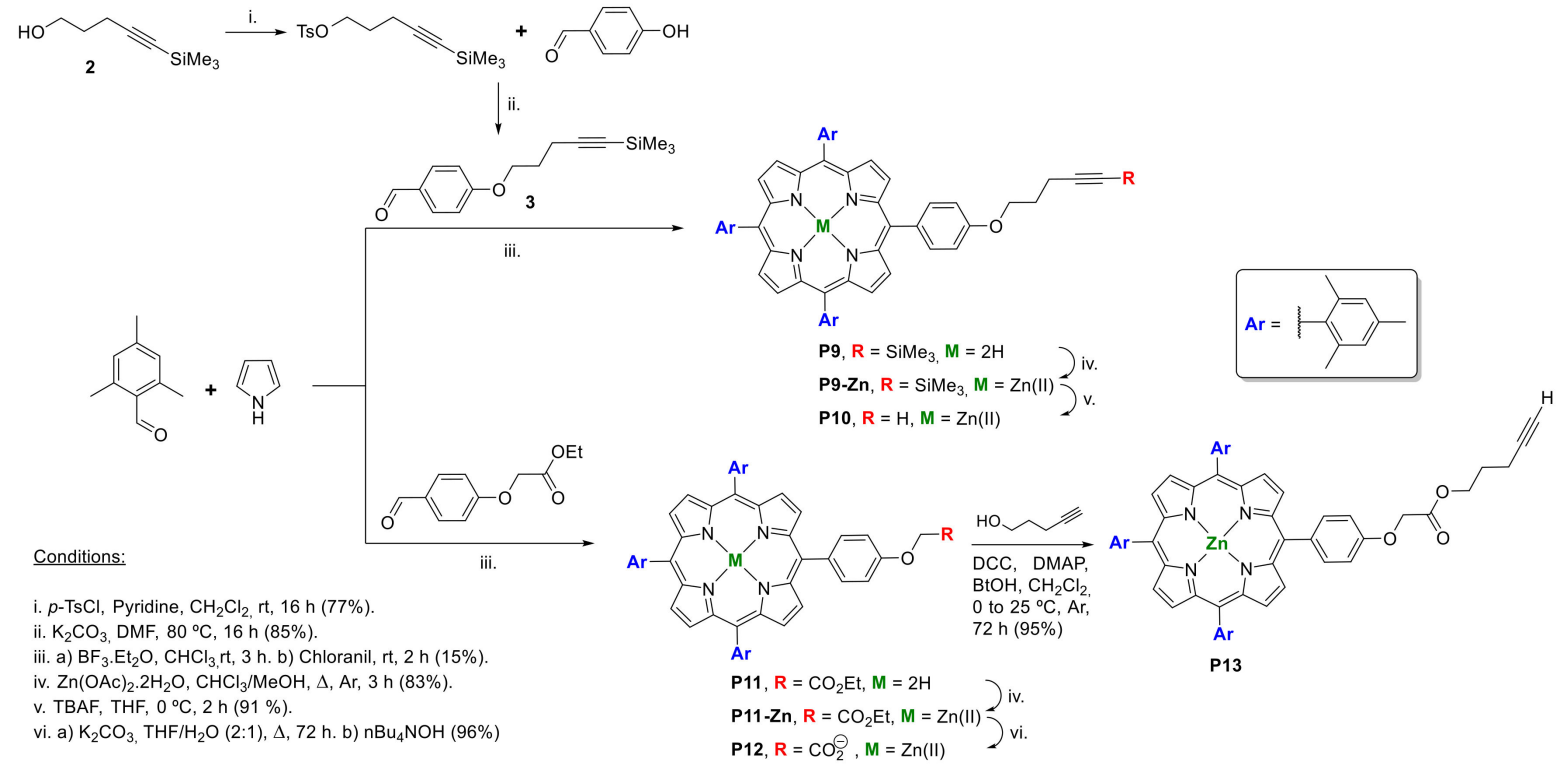
Synthetic approaches to prepare mono-substituted alkyne *meso*-tetraarylporphyrins.

Nierengarten and co-workers also developed a synthetic approach to prepare pillar[5]arene derivatives peripherally decorated with porphyrin units [24]. The synthetic route started by the preparation of the alkyne-substituted A_3_B-type porphyrin derivatives **P10** and **P13** (Scheme 5). The preparation of the Zn(II) porphyrin-alkyne complexes began with the preparation of the aldehyde **3** by tosylation of 5-(trimethylsilyl)pent-4-yn-1-ol **2** followed the Williamson etherification of the tosylate with 4-hydroxybenzaldehyde affording **3** in an 85% yield. The next step involved the condensation of pyrrole, mesitaldehyde and **3** in the presence of catalytic amounts of BF_3_.Et_2_O under the conditions described by Lindsey [25]. The porphyrinic core **P9** was metalated with Zn(II) and the obtained product was reacted with tetrabutylammonium fluoride (TBAF) in THF to give the porphyrin derivative **P10** with a terminal alkyne moiety (Scheme 5) [24].

**Scheme 6:**
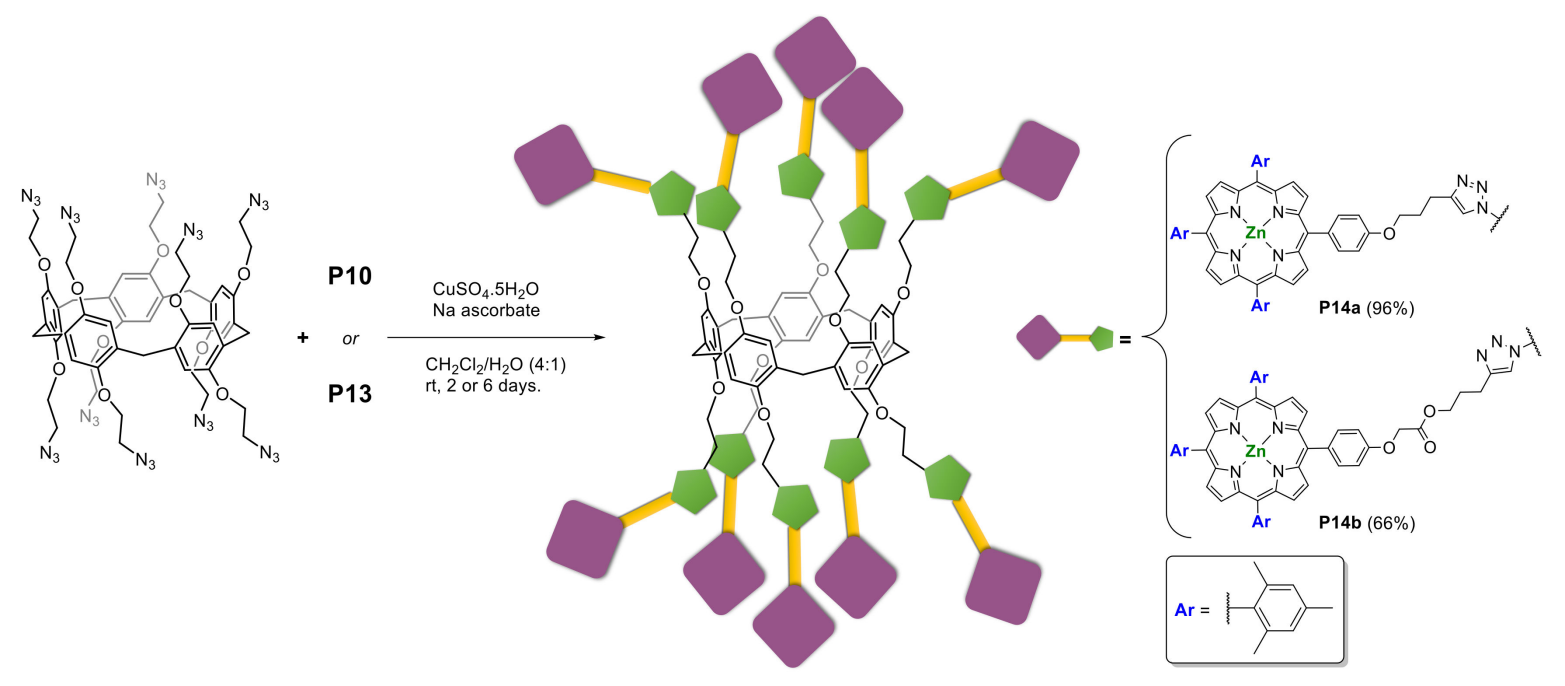
Synthesis of pillar[5]arene **P14** containing porphyrin–triazolyl motifs.

A similar strategy was used to prepare the Zn(II) porphyrin-alkyne complex **P11-Zn**. The latter derivative, by basic hydrolysis and tetrabutylammonium hydroxide treatment, gave rise to the corresponding carboxylate **P12** as its tetrabutylammonium salt. Esterification of **P12** with pent-4-yn-1-ol under classical peptide conditions with *N*,*N’*-dicyclohexylcarbodiimide (DCC), 4-(dimethylamino)pyridine (DMAP), and 1-hydroxybenzotriazole (HOBt) afforded the Zn(II) porphyrin complex **P13** (Scheme 5) [24]. The Zn(II) porphyrin-alkyne complexes **P10** and **P13** were grafted to azido pillar[5]arene building blocks via CuAAC reaction, affording the ten porphyrin-triazole rings **P14a** and **P14b** in 96% and 66% yield, respectively (Scheme 6).

Photophysical studies with the former products have revealed that derivative **P14b** showed a temperature-dependent intramolecular binding between the 1,2,3-triazole moieties and Zn(II)-porphyrin core. These intramolecular interactions are favored at low temperature; however, at higher temperatures, the Zn(II) porphyrin-triazole coordination is broken and the molecules adopt an open flower-type conformation. The intramolecular coordination can be avoided by the addition of an external chemical stimulus, such as 1-phenylimidazole, a stronger ligand when compared with 1,2,3-triazole moieties [24].

**Scheme 7:**
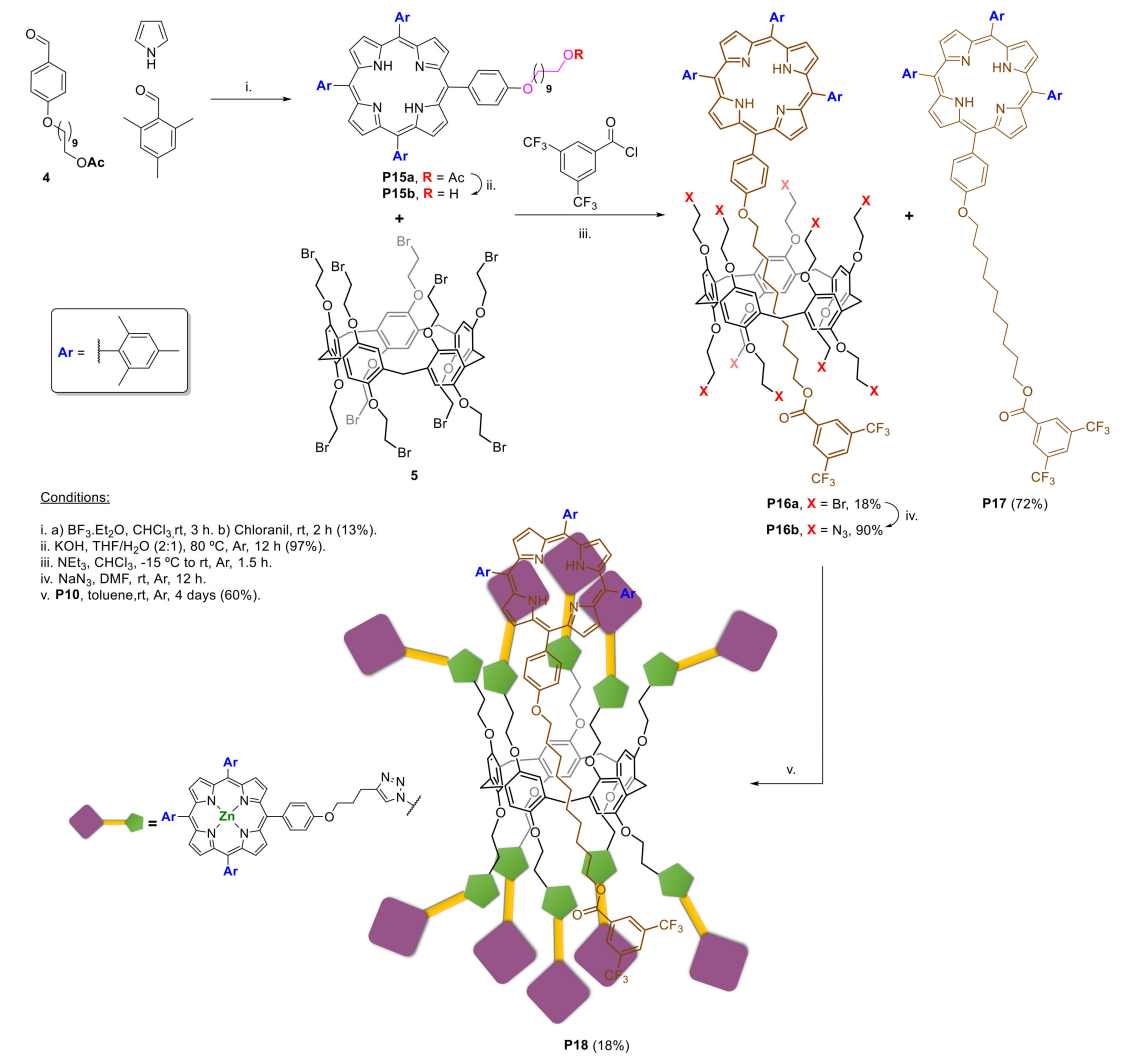
Synthetic approach to porphyrin [2]rotaxane derivative **P18**.

The search for light-harvesting devices led Nierengarten and co-workers [26] to the synthesis of rotaxanes bearing porphyrin moieties by using click chemistry and supramolecular self-assembly approaches (Scheme 7). The authors performed the statistical condensation of pyrrole, mesitaldehyde and aldehyde **4**, catalyzed by BF_3,_ followed by oxidation of the porphyrinogen intermediate, affording the corresponding porphyrin-decyloxy acetate derivative **P15a**. Hydrolysis of **P15a** with KOH provided the hydroxydecyloxy porphyrin derivative **P15b**.

Reaction of **P15b** with 3,5-bis(trifluoromethyl)benzoyl chloride in the presence of the bromo pillar[5]arene **5** and triethylamine gave rise to [2]rotaxane **P16a** (18% yield). The axle-type derivative **P17** was also isolated at a 72% yield. When the same conditions were extended to the pillar[5]arene peripherally functionalized with porphyrinic moieties **P14a** only the axle **P17** was isolated [26].

The target azide derivative **P16b** was obtained at a 90% yield by treating compound **P16a** with NaN_3_. The clickable [2]rotaxane scaffold **P16b** was then reacted with Zn(II)-porphyrin-alkyne **P10** (see structure in Scheme 5) in the presence of [Cu(phen)(PPh_3_)_2_)](BF_4_) in toluene affording derivative **P18** at an 18% yield (Scheme 7). The obtained [2]rotaxane peripherally decorated with porphyrin units presents adequate features to act as an antenna for a light-harvesting device, able to transfer energy from the Zn(II) porphyrin moieties to the core [26].

**Scheme 8:**
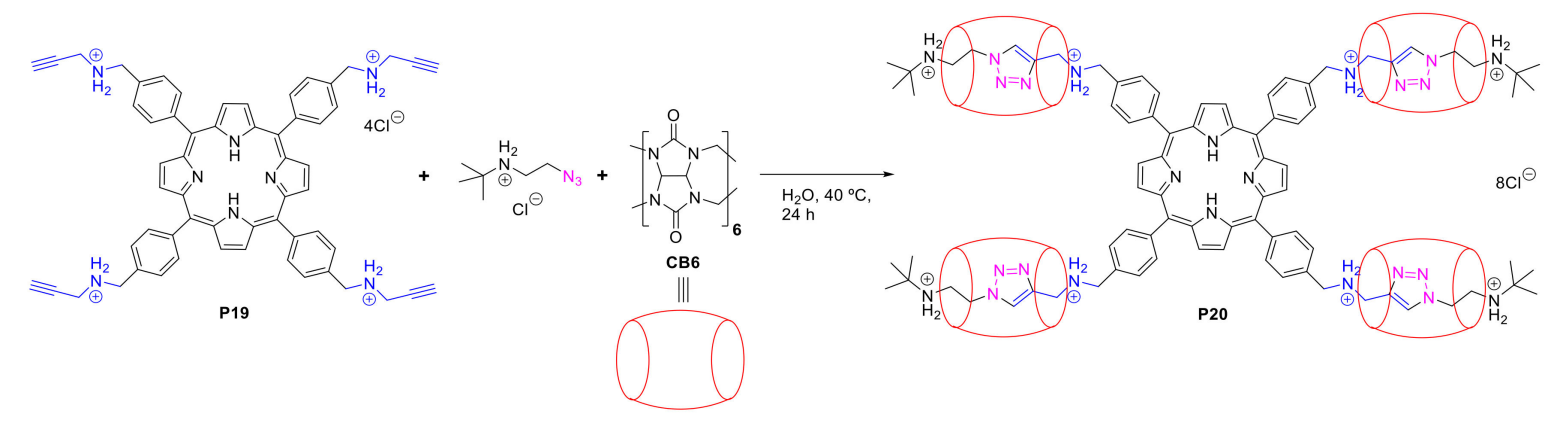
Preparation of porphyrin-based cucurbit[6]uril [5]rotaxane **P20**.

More recently, Tuncel and co-workers reported the synthesis of a cucurbit[6]uril (**CB6**) [5]rotaxane **P20** bearing a tetracationic porphyrin decorated with triazole units [27]. The [5]rotaxane **P20** was synthesized as depicted in Scheme 8; the tetracationic porphyrin derivative **P19** was reacted with *tert*-butyl azidoethylammonium chloride and **CB6** in water for 24 h at 40 °C (Scheme 8). In this reaction the **CB6** has a dual function; it is the catalyst for the 1,3-dipolar cycloaddition between the azido and the alkynyl groups, leading to the formation of the 1,2,3-triazole ring, and it is the macrocycle for the inclusion of the four triazole rings. The porphyrin-core [5]rotaxane **P20** was obtained at an 87% yield [27].

The photosensitizing properties of the water-soluble compound **P20** was assessed on the photoinactivation of *Escherichia coli* (*E. coli*), a Gram-negative bacterial strain, *Bacillus subtilis (B. subtilis*), a Gram-positive bacterium, and breast cancer cell line MCF7. It displayed no cytotoxicity in the dark but under light **P20** showed to be an efficient photosensitizer, even at low concentrations, demonstrating the potential for being used in both cancer photodynamic therapy (PDT) and photodynamic inactivation of microorganisms (PDI) [27].

**Figure 1:**
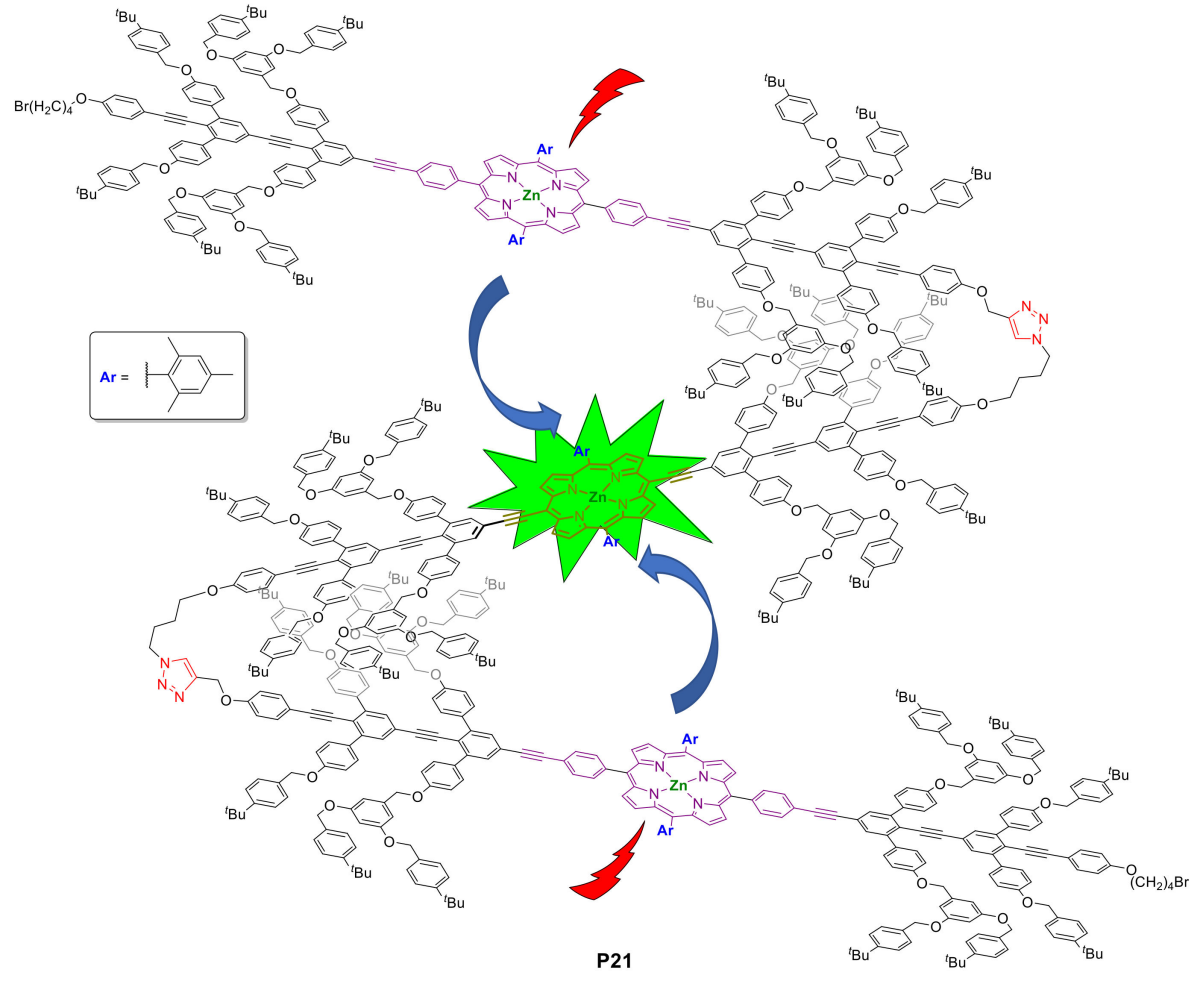
Structure of trimeric porphyrin—dendritic derivative **P21**.

Kozaki and co-workers reported the synthesis of a trimeric assembly of dendritic porphyrins with two kinds of porphyrin cores [28]. Compound **P21** (Figure 1) was prepared using a CuAAC reaction between a dendrimer having a Zn(II) diethynyldiphenylporphyrin core with two azide terminals and two equivalents of a dendrimer having a Zn(II) tetraphenylporphyrin core with one ethynyl terminal. This reaction was performed using copper sulfate (10 equiv.) and sodium ascorbate (45 equiv.) in dry DMF at room temperature for two days to afford trimer **P21** as a black solid at a 66% yield. Absorption and fluorescence studies revealed that trimer **P21** has a solvent-dependent conformation; while extended forms are favored in 1,1,2,2-tetrachloroethane (polar), the folded conformation is predominant in toluene (nonpolar). These different conformations explain why the singlet energy transfer from the tetraphenylporphyrins to the central diphenylporphyrin takes place with higher quantum efficiency in toluene than in 1,1,2,2-tetrachloroethane.

**Scheme 9:**
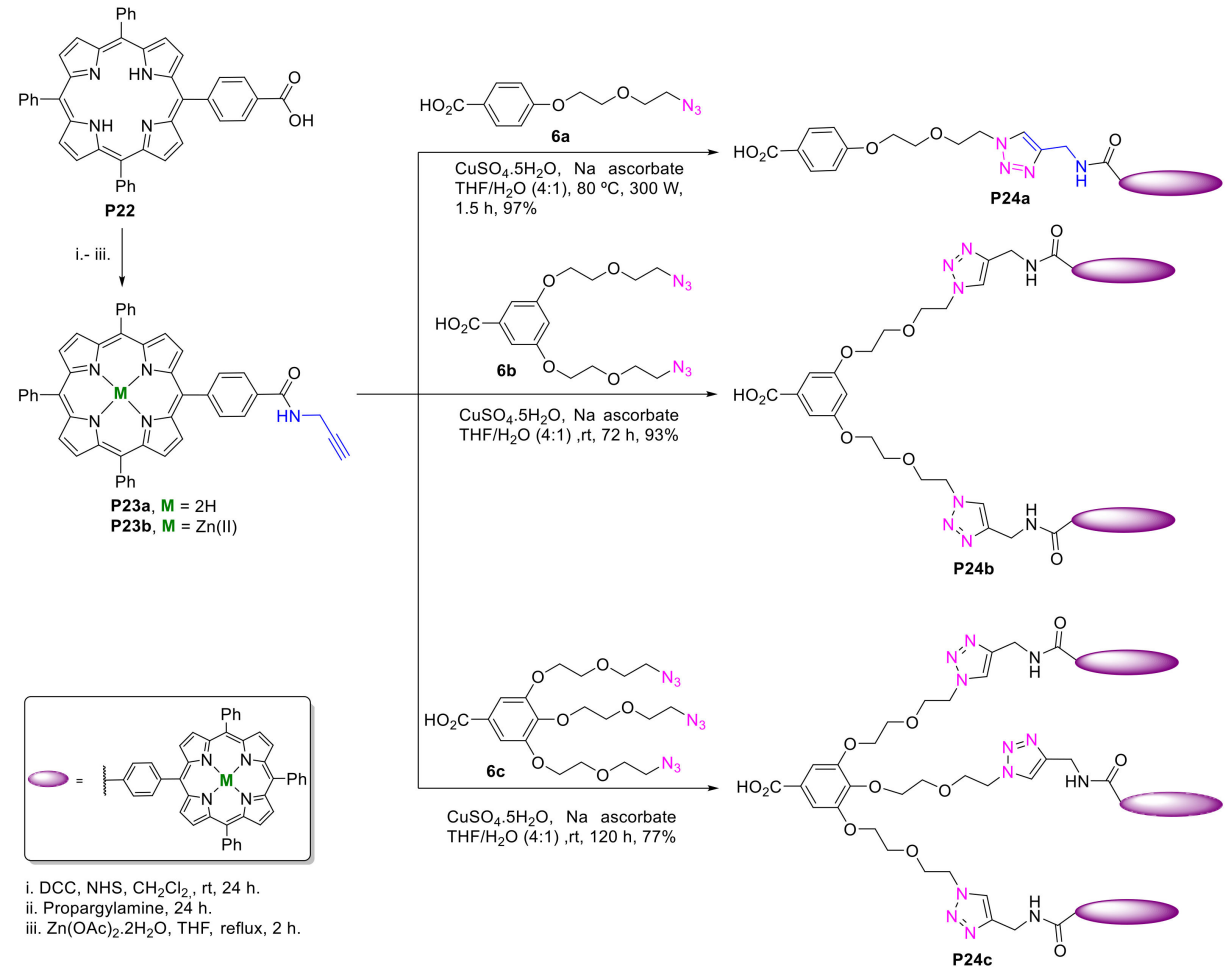
Synthetic approach to prepare mono-, di- and tri-substituted porphyrin-triazolyl entities **P24**.

Acherar and co-workers reported the preparation of porphyrin-based building blocks using a click chemistry synthetic strategy [29]. The authors started by the preparation of Zn(II) porphyrin complex **P23b** (Scheme 9). For that the 5-(4-carboxyphenyl)-10,15,20-triphenylporphyrin **P22** was converted into the propargyl-amide derivative **P23a** (96% yield) by reaction with an excess of propargylamine after the in situ activation of the carboxyl group of **P22** with DCC and NHS. Compound **P23a** was metalated with the Zn(II) ion affording derivative **P23b** in quantitative yield. The reaction of porphyrin **P23b** and the mono-, di- and tri-substituted azides **6a**–**c** was carried out in the presence of catalytic amounts of CuSO_4_ and sodium ascorbate, 0.1 and 0.5 equiv., respectively. During the optimization of the reactional conditions the authors observed that for compound **P24a** the better conditions involved microwave (MW) irradiation. For compounds **P24b**,**c** the best conditions involved stirring at rt for three to five days. The described conditions allowed to obtain derivatives **P24a**–**c** in yields ranging from 77% to 97%. Photophysical studies with **P24a**–**c** carried out by the same group have revealed that the number of porphyrin units influences their photophysical properties [29].

**Figure 2:**
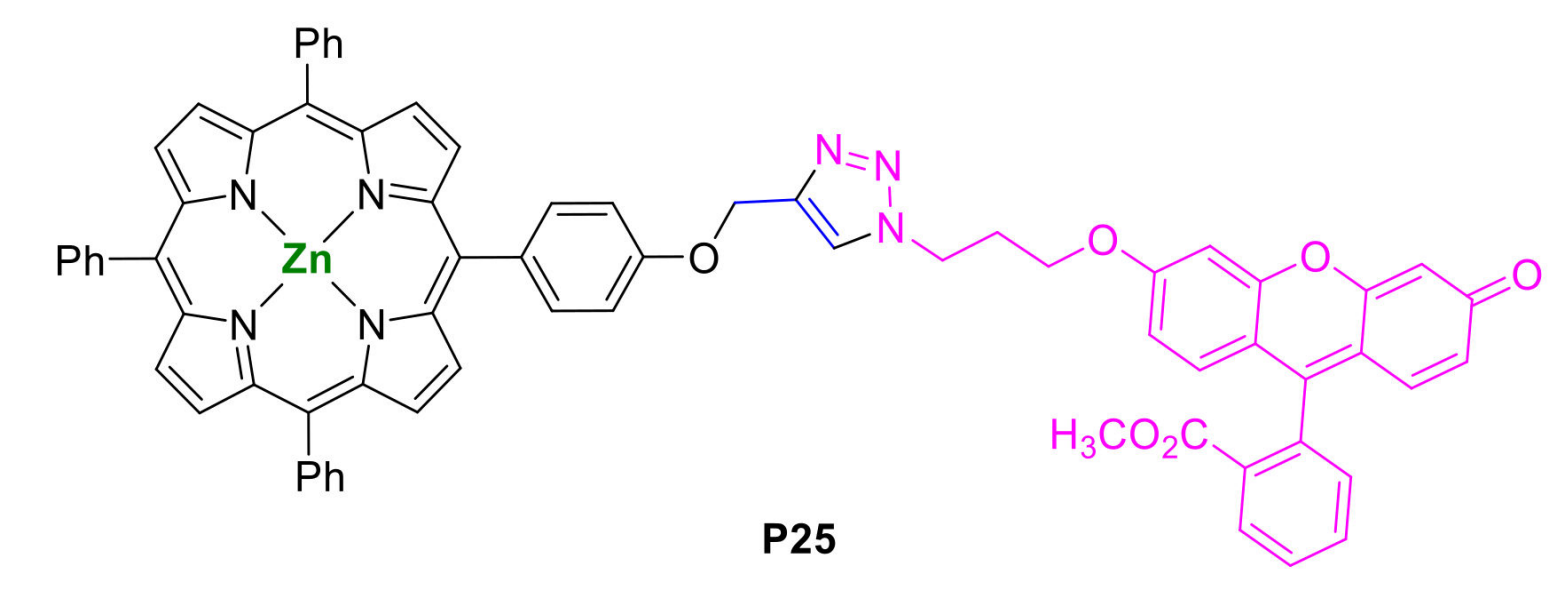
Structure of the porphyrin-fluorescein dyad **P25**.

Leroy-Lhez and co-workers followed a similar synthetic approach to attach a fluorescein-azide moiety to a porphyrin macrocycle bearing a propargyl moiety. The reaction was carried out under CuAAC conditions, affording the porphyrin–fluorescein dyad **P25** at a 91% yield (Figure 2). Photophysical studies supported by density functional theory (DFT) calculations showed strong evidence of efficient energy transfer (~40%) between the porphyrin and fluorescein moieties [30].

**Scheme 10:**
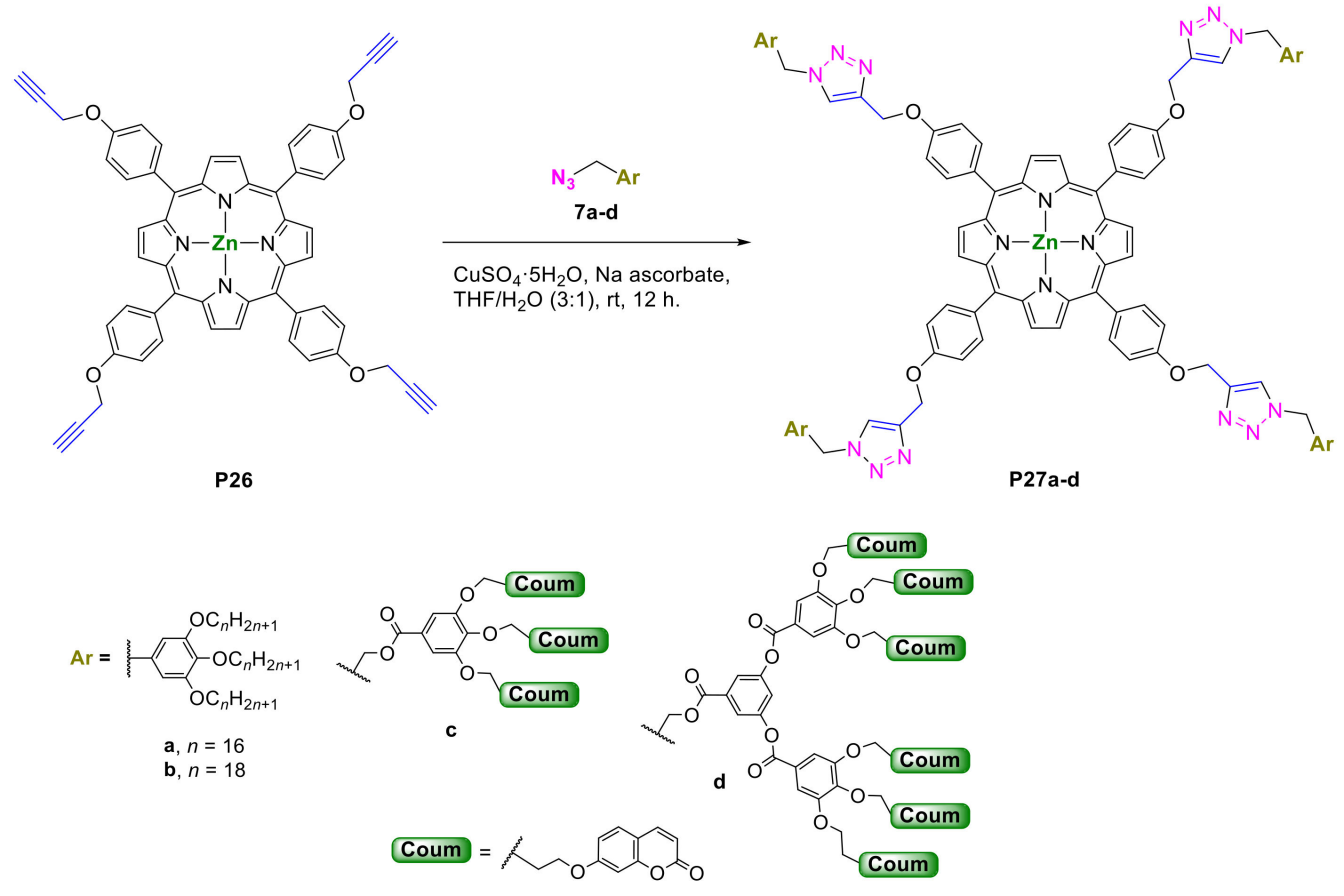
Porphyrin-based dendrimers **P27** with liquid crystal behavior.

The research groups of Cheng [31] and Serrano [32] used the porphyrin-propargyl derivative **P26** to prepare porphyrin-based dendrimers peripherally functionalized with alkyl chains linked by triazolyl rings and exhibiting liquid crystal behavior. Both groups developed synthetic routes using porphyrin **P26** as the starting reagent for the CuAAC reaction with azide derivatives **7a**–**d** (Scheme 10).

Cheng [31] prepared porphyrin derivatives **P27a**,**b** bearing four 1,2,3-triazole moieties with three terminal alkyl chains (*n* = 16 or 18) in each one of the *meso* positions that self-assembled into a columnar hexagonal packaging. When the chemosensorial ability of derivative **P27b** (*n* = 18) was evaluated in solution towards a series of metal(II) cations, that compound showed a selective emission quenching in the presence of Cu^2+^ ions. Based on these results the authors prepared a porphyrin-based organogel in 1,4-dioxane due to the formation of *J*-aggregated entities assembled in a flower-like sphere by conjugation of hydrogen bonds, van der Waals and π-π-interactions. When this gel was explored as a chemosensor towards Cu^2+^ ions, a quenching in the emission was observed due to the interaction of Cu^2+^ ions with triazole moieties, which results in the breakdown of gelator molecules.

The authors explored also the co-assembly properties of compound **P27a**,**b** with C_70_ or 4,7-di-4-pyridyl-2,1,3-benzothiadiazole ligand. All systems obtained showed mesomorphic properties and a sponge-like morphology. However, the films obtained with 4,7-di-4-pyridyl-2,1,3-benzothiadiazole present an unordered morphology, while with C_70_ a uniform alignment of columns in a columnar hexagonal grid was attained that might lead to anisotropic charge carrier transport along the molecular stacking. Due to the properties presented by compound **P27b**, it can be investigated for different applications in organic electronic devices or as a chemosensor [31].

Serrano and co-workers prepared porphyrin-core dendrimer derivatives **P27c**,**d** with three or six alkyl chains with a terminal coumarin moiety at each one of the *meso* positions. These compounds were synthesized by a CuAAC reaction between porphyrin-alkyne **P26** and the appropriate azido dendrons derivatives **7c**,**d** [32]. The tetra-substituted porphyrin-based functionalized **P27c** and second-generation dendrimers **P27d** were isolated in 91% and 71% yields, respectively, and their liquid crystalline behavior explored (Scheme 10). The two derivatives displayed mesogenic properties and the authors found that the coumarin moieties play a key role for the liquid crystal performance with a high contribution to the discotic nematic mesophases showing high hole mobility values when compared with an analogous derivative peripherally functionalized with dodecyloxy alkyl chains. Additionally, the coumarin moieties have an antenna effect, inducing an energy transfer process after excitation, presenting appropriate features to be used in optoelectronic applications [32].

**Scheme 11:**
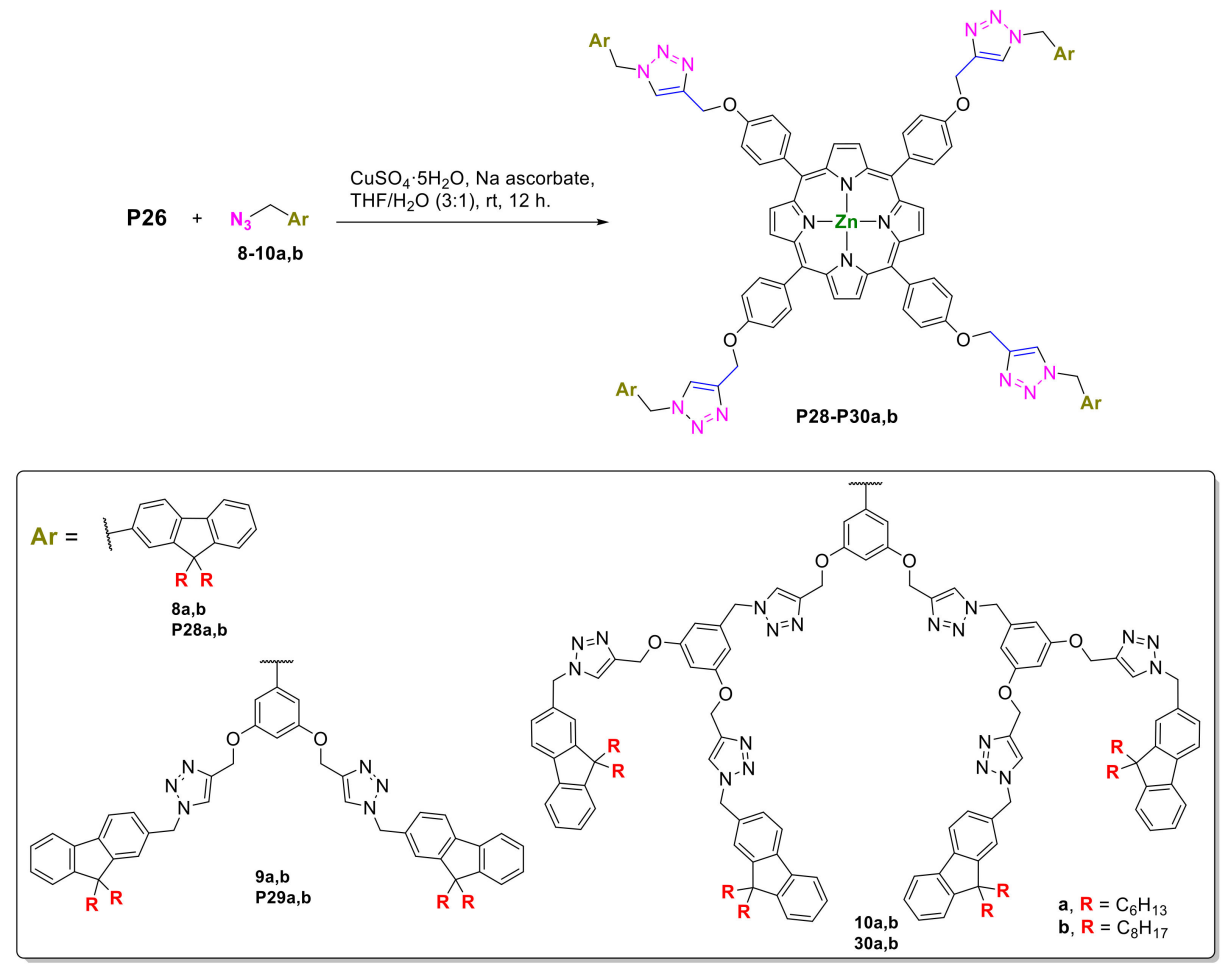
Synthesis of porphyrin–fluorenodendrimers conjugates **P28**–**P30a**,**b**.

Rajakumar described the preparation of porphyrin-based triazole—bridged fluorenodendrimers **P28a**,**b**, **P29a**,**b** and **P30a**,**b** [33]. The porphyrin-fluorenodendrimers conjugates **P28**–**P30a**,**b** were prepared by reaction of the porphyrin-propargyl derivative **P26** with a series of dialkyl-fluorene azido dendrons **8**–**10a**,**b** through a CuAAC reaction. The reaction was carried out in the presence of CuSO_4_·5H_2_O (5 mol%) and sodium ascorbate (10 mol%) in a mixture of THF/H_2_O (3:1) for 12 h at room temperature. Compounds **P28**–**P30a**,**b** were isolated in yields ranging from 71% to 82% (Scheme 11).

An increase in the number of fluorene moieties around the porphyrin core induced the enhancement of the absorption and fluorescence intensities. The antiproliferative activity of porphyrin–fluorenodendrimers against the PA-1 human ovarian teratocarcinoma cell line was accessed; the second-generation porphyrin-based dendrimers **P30a**,**b** showed a more efficient inhibition of cancer cell growth [33].

**Figure 3:**
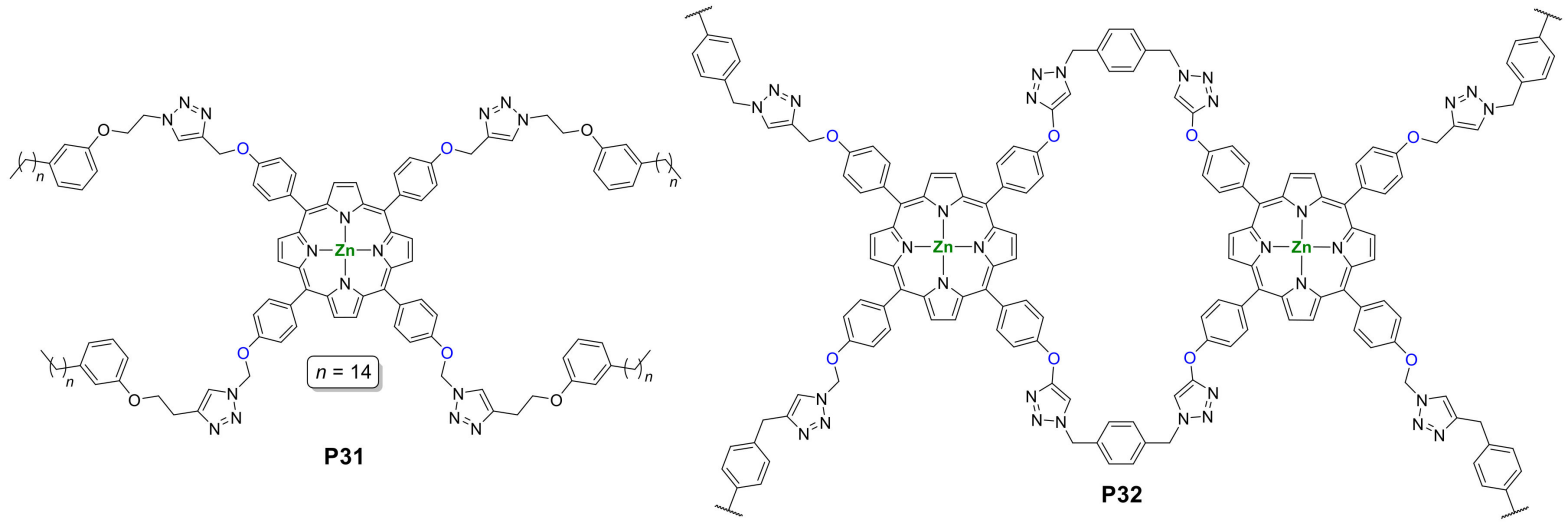
Structures of porphyrin-cardanol hybrid **P31** and porphyrin-based polymer **P32**.

More recently, Prabakaran and co-workers reported the synthesis of porphyrin derivative **P31** bearing four H-cardanol units (Figure 3), a waste by-product of the cashew industry, linked by triazolyl bridges prepared via a CuAAC reaction [34]. *J*-type aggregates formation is induced when compound **P31** was studied in polar protic and aprotic solvents, due to the long alkyl chains of the H-cardanol moieties. However, compound **P31** is soluble in non-polar solvents, namely hexane, due to its fat-like behavior when compared with other analogous compounds with smaller alkyl chains (*n* = 2). This compound showed adequate photophysical properties for a potential medicinal application as a photosensitizer in PDT.

Ye and co-workers prepared porphyrin-based porous polymers by the reaction of porphyrin **P26** with 1,4-bis(azidomethyl)benzene via CuAAC reaction in the presence of *N*,*N´*,*N′*,*N″*,*N″*-pentamethyl-diethylenetriamine (PMEDTA) and CuBr in DMF at 100 °C for 48 h. The obtained porous Zn(II) porphyrin polymer **P32** (Figure 3) is able to interact and to act as protein adsorbent, showing potentiality for protein immobilization and other applications in pharmaceutical or food industries [35].

**Scheme 12:**
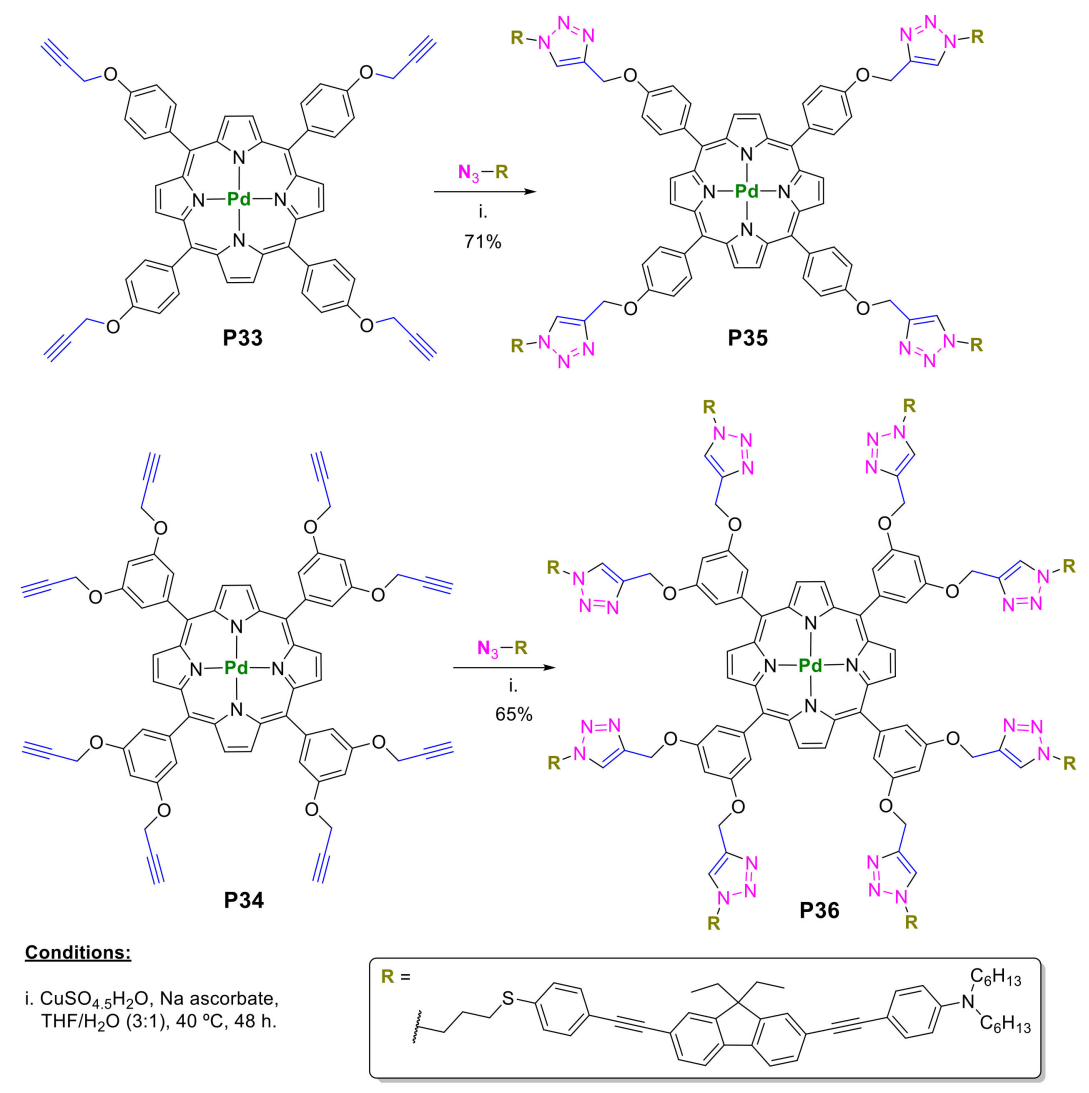
Synthesis of porphyrin derivatives **P35** and **P36** bearing four or eight 9,9-diethylfluorene groups, respectively.

The synthesis of the Pd(II) porphyrin complexes **P35** and **P36** was described by Bretonniére and co-workers [36]. The synthetic strategy involved the preparation of the Pd(II) complexes **P33** and **P34** from the hydroxylated porphyrin precursors, followed by the functionalization of the metalloporphyrins with alkyne moieties by a nucleophilic substitution reaction with propargyl bromide in the presence of K_2_CO_3_. The next step involved the CuAAC reaction of **P33** and the octa-substituted **P34** alkynyl porphyrins with the azido-9,9-diethylfluorene derivative in the presence of CuSO_4_.5H_2_O and sodium ascorbate at 40 °C for 48 h. This allowed the preparation of the porphyrin derivatives **P35** and **P36** at a 71% and 65% yield, respectively (Scheme 12). The 9,9-diethylfluorene moiety grafted to the Pd(II) porphyrin acts as an artificial antenna enhancing the two-photon absorption when excited at 800 nm and induces an energy transfer to the porphyrin core through a Förster Resonance Energy Transfer (FRET) process [36].

**Scheme 13:**
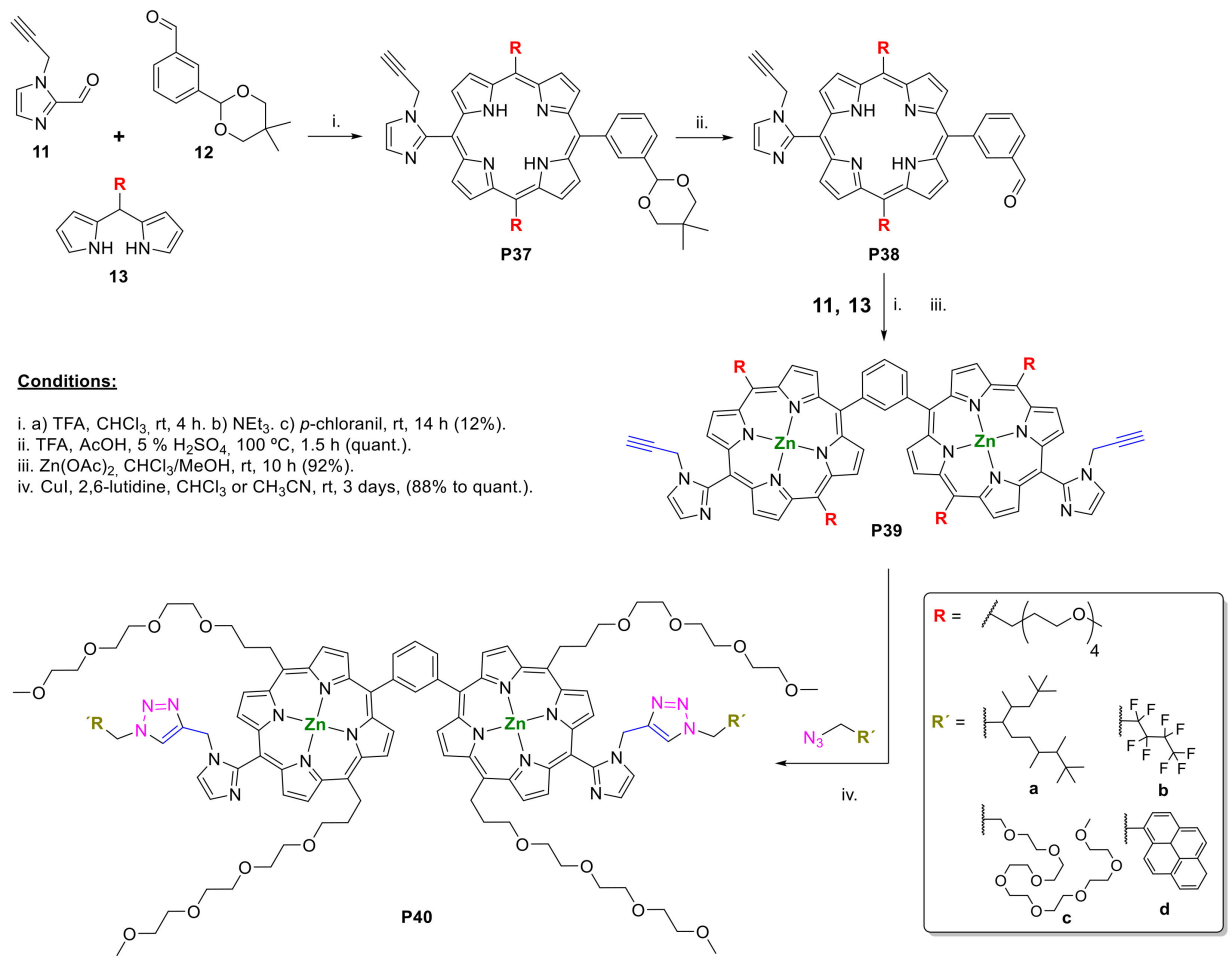
Modification of Gable-type porphyrins via CuAAC reactions.

Satake and co-workers reported the preparation of the Gable-type porphyrin **P39** bearing two imidazole-alkynyl moieties, thus becoming available to be modified via a CuAAC functionalization [37]. The A_2_BC porphyrin **P37** was prepared by the condensation of dipyrromethane **13** with aldehydes **11** and **12** in the presence of trifluoroacetic acid (TFA), followed by acidic deprotection of the acetal protecting group thus affording the corresponding formyl derivative **P38**. Then, compound **P38** was submitted to a further TFA catalyzed condensation with dipyrromethane **13** and aldehyde **11** under the same conditions affording the alkynyl porphyrin **P39**. Its Zn(II) complex was prepared by treatment with Zn(OAc)_2_ salt and functionalized through a CuAAC reaction with an alkyl, nonfluorinated, oligoether group and pyrene derivative moieties by the reaction of the appropriate azide in the presence of CuI and 2,6-lutidine (Scheme 13). Gable-type porphyrins **P40** with a triazolyl bridge were obtained in high yields (>88%) after stirring for three days at room temperature. The authors emphasized the utility of the obtained porphyrin derivatives for the preparation of dynamic combinatorial chemistry leading to supramolecular systems by a self-assembly approach, capable of detecting interactions between a target biomaterial (e.g., proteins or nucleic acids) and a ligand [37].

**Scheme 14:**
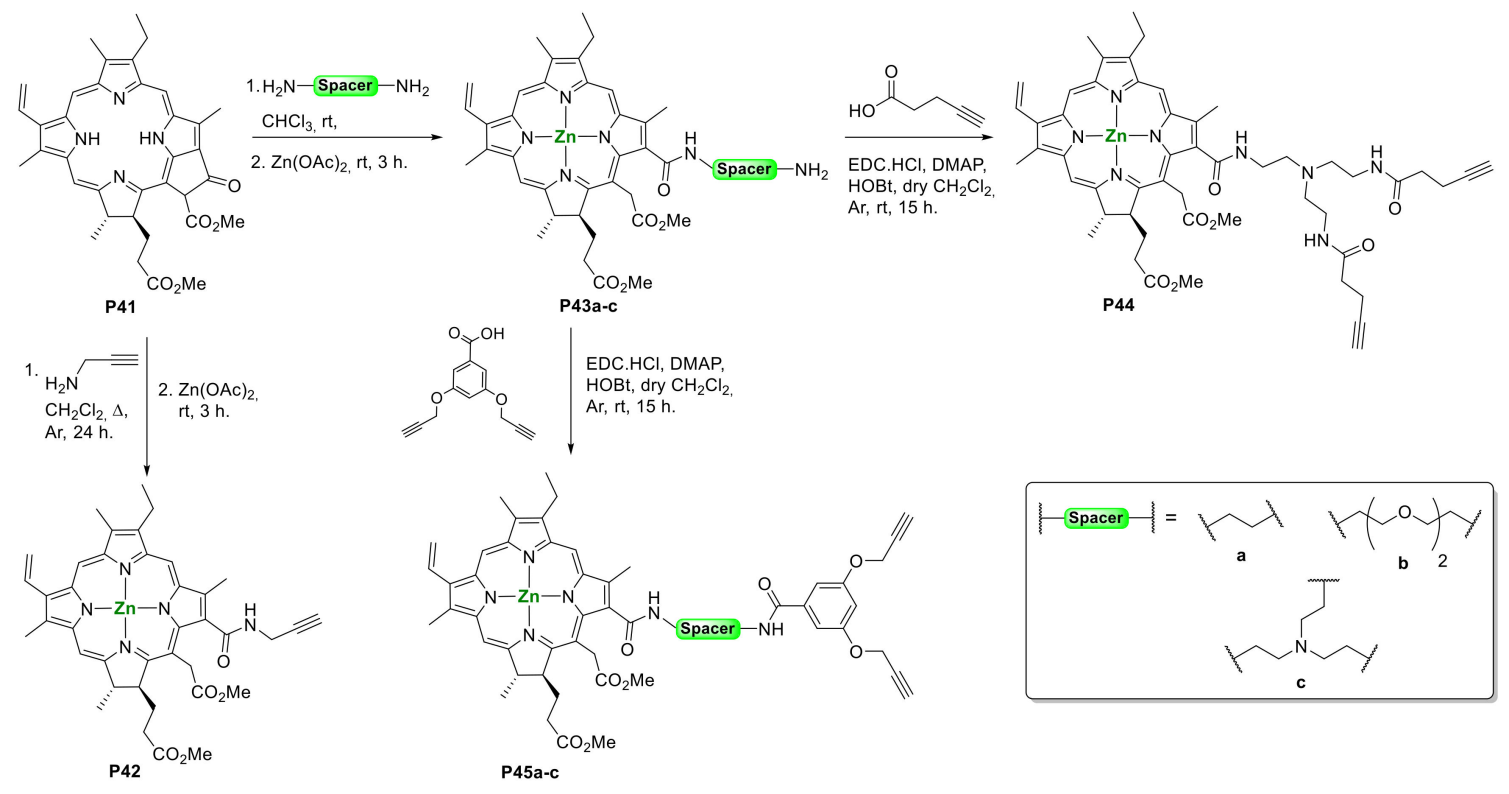
Synthesis of chlorin-amides **P42**, **P44** and **P45a**–**c** bearing alkyne moieties.

Methyl pheophorbide *a* **P41** was used as the precursor for the synthesis of chlorin derivatives bearing one, two or four quinazoline moieties linked by triazolyl rings via CuAAC reactions (Scheme 14). In the first step, methyl pheophorbide *a* **P41** reacted with propargylamine or a series of diamines affording the corresponding chlorin-amide derivatives **P42** and **P43** by exocyclic ring-opening; in the second step the free-base amide derivative was metalated with Zn(II). Finally, the Zn(II) chlorin-amide complexes **P43a**–**c** were submitted to an acylation reaction at the terminal free amino groups under Steligkich conditions that involved a carboxyl group (pent-4-ynoic acid or 3,5-bis(propargyloxy)benzoic acid), EDC, DMAP and HOBt. After 15 h at rt the corresponding amides **P44** and **P45** were obtained in yields ranging from 88% to 93% [38].

**Scheme 15:**
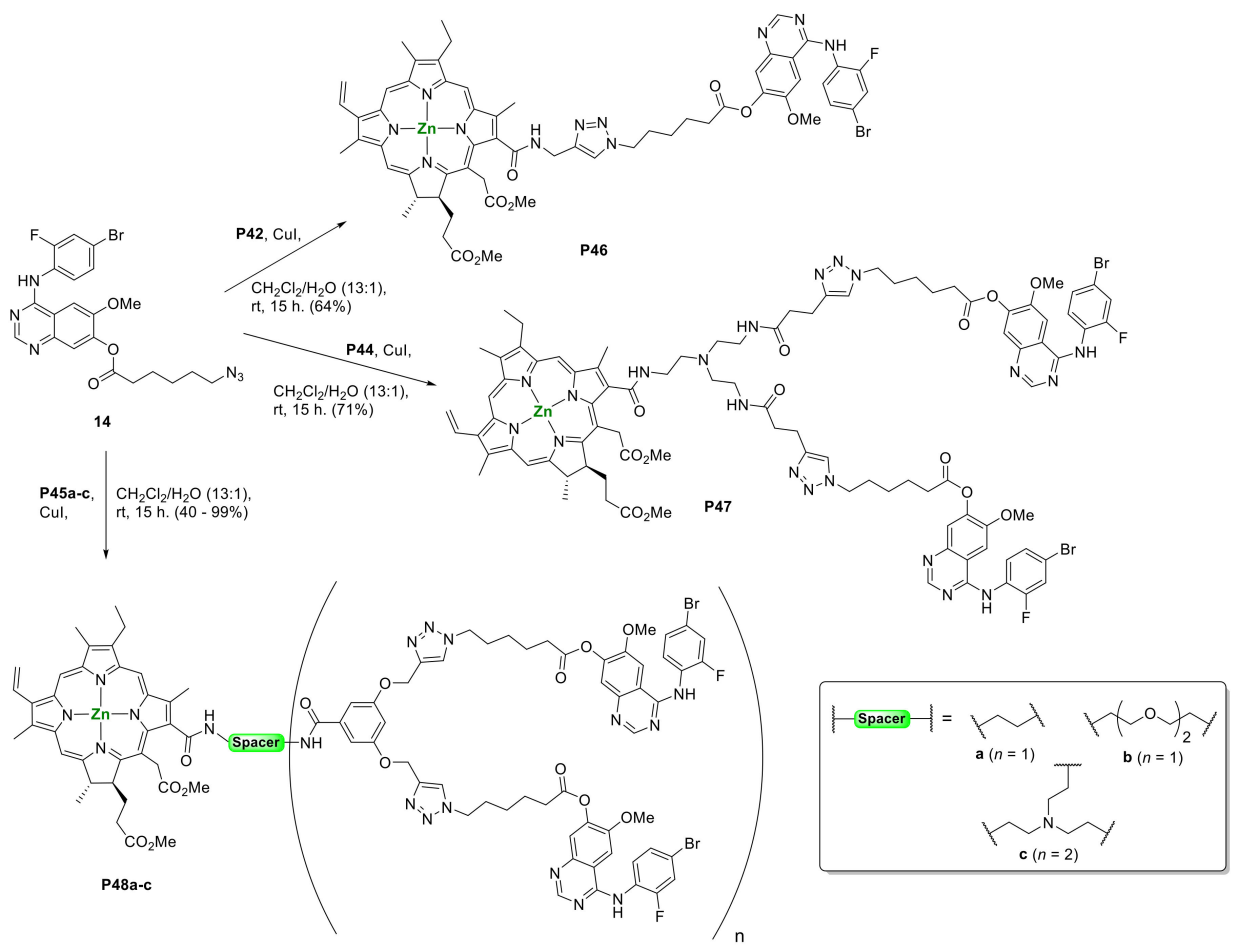
Synthesis of chlorin-triazole-quinazoline conjugates.

The azide-functionalized (arylamino)quinazoline **14** was then connected to the chlorin-alkyne derivatives **P42**, **P44** and **P45a**–**c** through click chemistry cycloaddition reactions in the presence of catalytic amounts of CuI after magnetic stirring for 15 h at room temperature, affording the corresponding chlorin bearing one (**P46**), two (**P47** and **P48a**,**b**) or four (**P48c**) triazole-quinazoline units in yields ranging from 40% to 99% (Scheme 15).

The quinazoline moiety attached to the chlorin derivatives is analogous to the unit present in the structure of Vandetanib, a drug approved for the treatment of metastatic medullary thyroid cancer and is known to be a selective epidermal growth factor receptor and vascular endothelial growth factor receptor ligand. The authors expected to find a synergic effect between the photosensitizer properties of the chlorin unit and the selectively cytotoxic agent against tumor cells due to the quinazoline moiety. The chlorin-triazole-quinazoline conjugates presented suitable photophysical properties for the target application; however, their low water solubility precludes the study of their biological activity [38].

**Figure 4:**
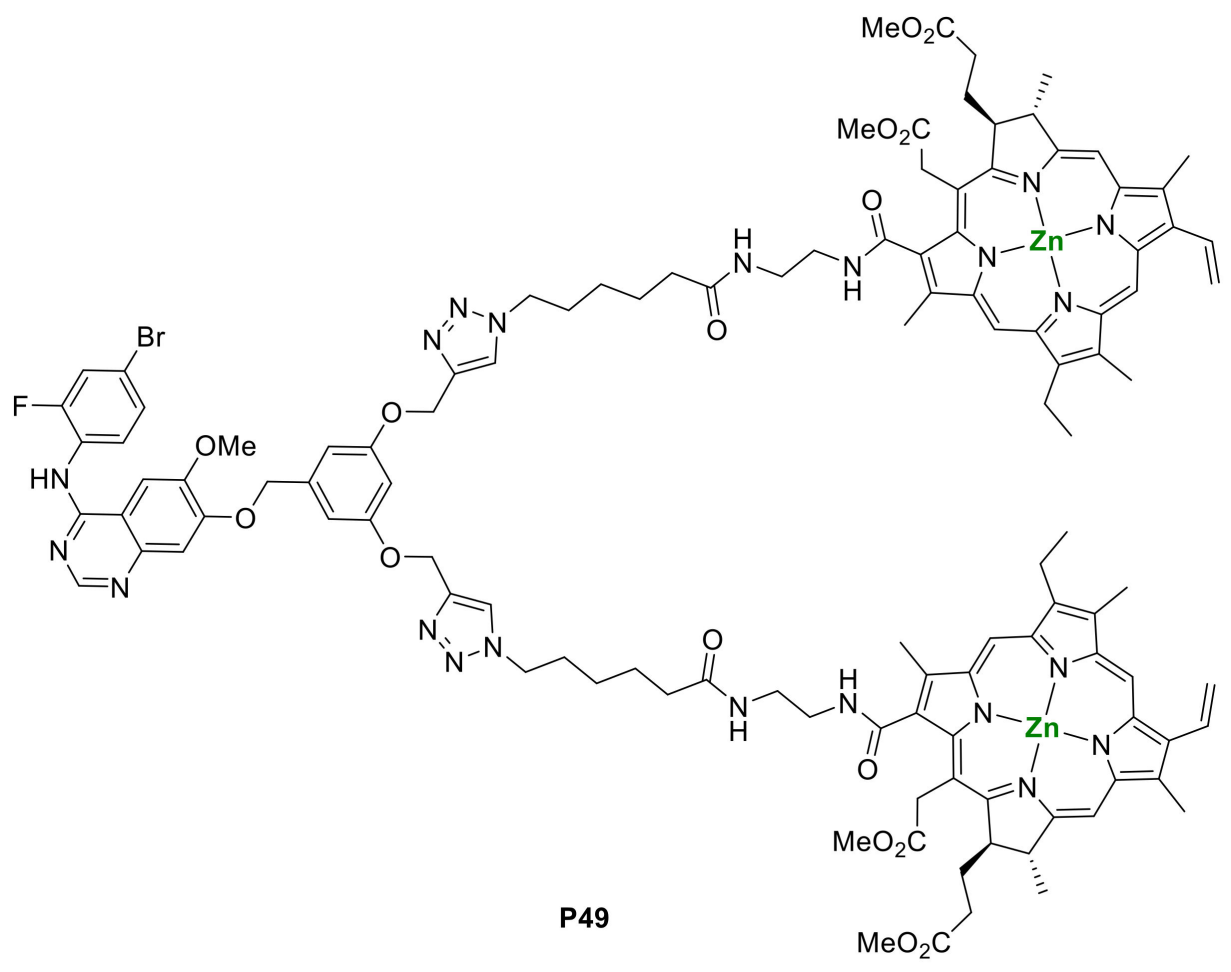
Structure of bis(chlorin)-quinazoline derivative **P49**.

The same group described the synthesis of the bis(chlorin)-quinazoline derivative **P49** (Figure 4, 57%) by reaction of the chlorin-azide derivative, prepared by reaction of chlorin **P43a** with 6-azidohexanoic acid, and a quinazoline di-alkyne derivative in the presence of catalytic amounts of CuI in a DMF/water (40:1) mixture [38].

#### 2.1.2. Carbohydrate-Porphyrin Conjugates

The click approach was also used to prepare porphyrin-carbohydrate conjugates. In most of the strategies reported, the alkynyl function is present in the porphyrin while the azide group appears in the carbohydrate component.

**Scheme 16:**
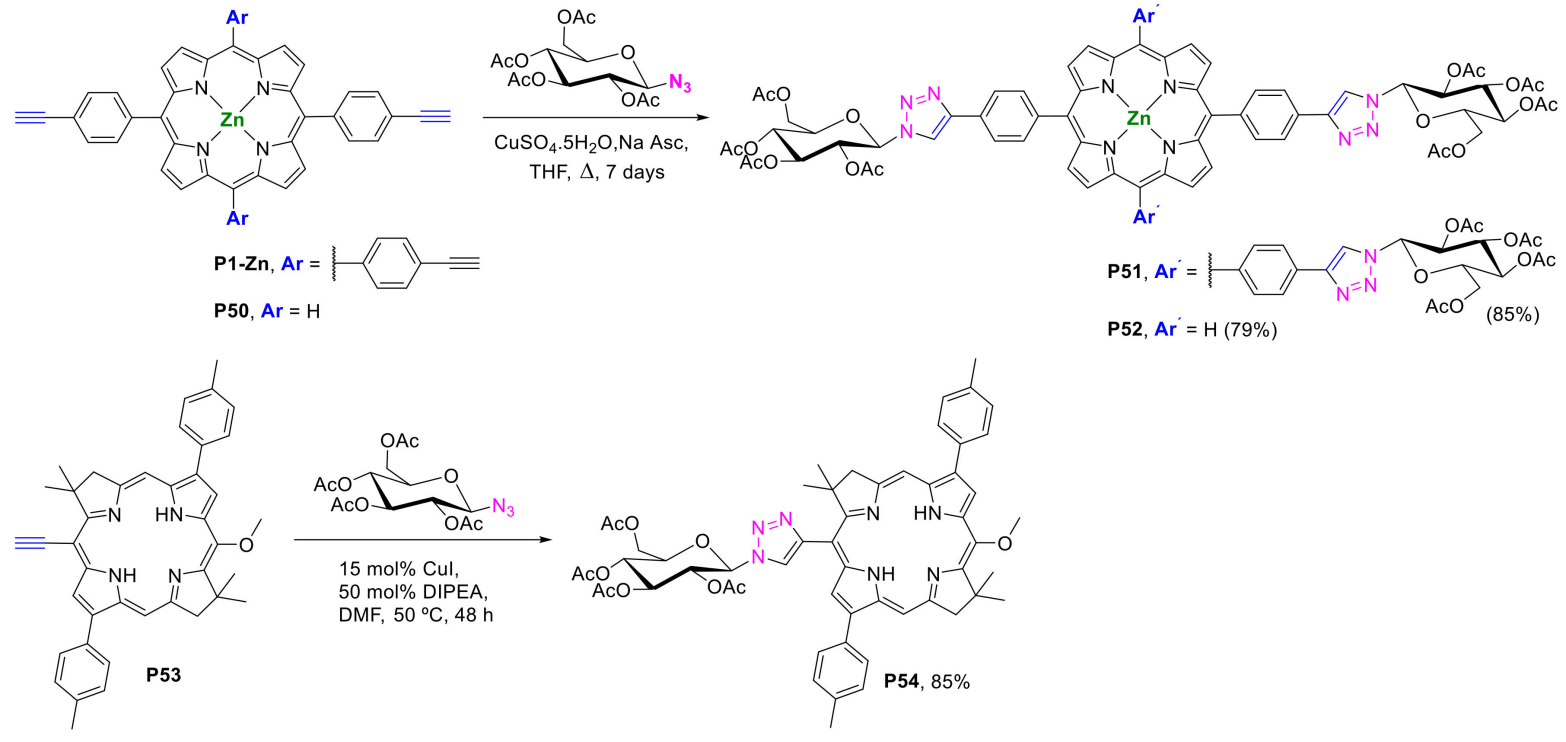
Synthesis of glucosylated porphyrins **P51** and **P52** and glucosylated bacteriochlorin **P54**.

For instance, in 2015, Snyder and co-workers used the click conditions to prepare the glucosylated porphyrins **P51** and **P52** in very good yields by the direct conjugation of 2,3,4,6-tetra-*O*-acetyl-β-d-glucosyl-1-azide with the Zn(II) complexes of the adequate (4-ethynylphenyl)porphyrins **P1**-**Zn** and **P50** (Scheme 16). The authors extended this approach to other per-*O*-acetylated sugar azides (galactose, lactose, and glucosamine) and referred that the use of the Zn(II) complexes was important to avoid the coordination of the porphyrin inner core with the copper ions present in click reactions [39]. In 2019, the same group extended the approach to the 5-(4-ethynylphenyl)porphyrin, 5-(4-ethynylphenyl)-10,15,20-triphenylporphyrin, 5,15-bis(4-ethynylphenyl)-10,20-diphenylporphyrin, 2-ethynyl-5,10,15,20-tetraphenylporphyrin [40]. This group also used click chemistry to prepare three reduced glycosylated macrocycles (e.g., **P54**) by direct conjugation of the per-*O*-acetylated sugar azides (glucose, galactose and lactose) with 5-ethynyl-15-methoxy-7,7,17,17-tetramethyl-2,12-bis(*p*-tolyl)bacteriochlorin (**P53**) as shown in Scheme 16 for **P54**.

**Scheme 17:**
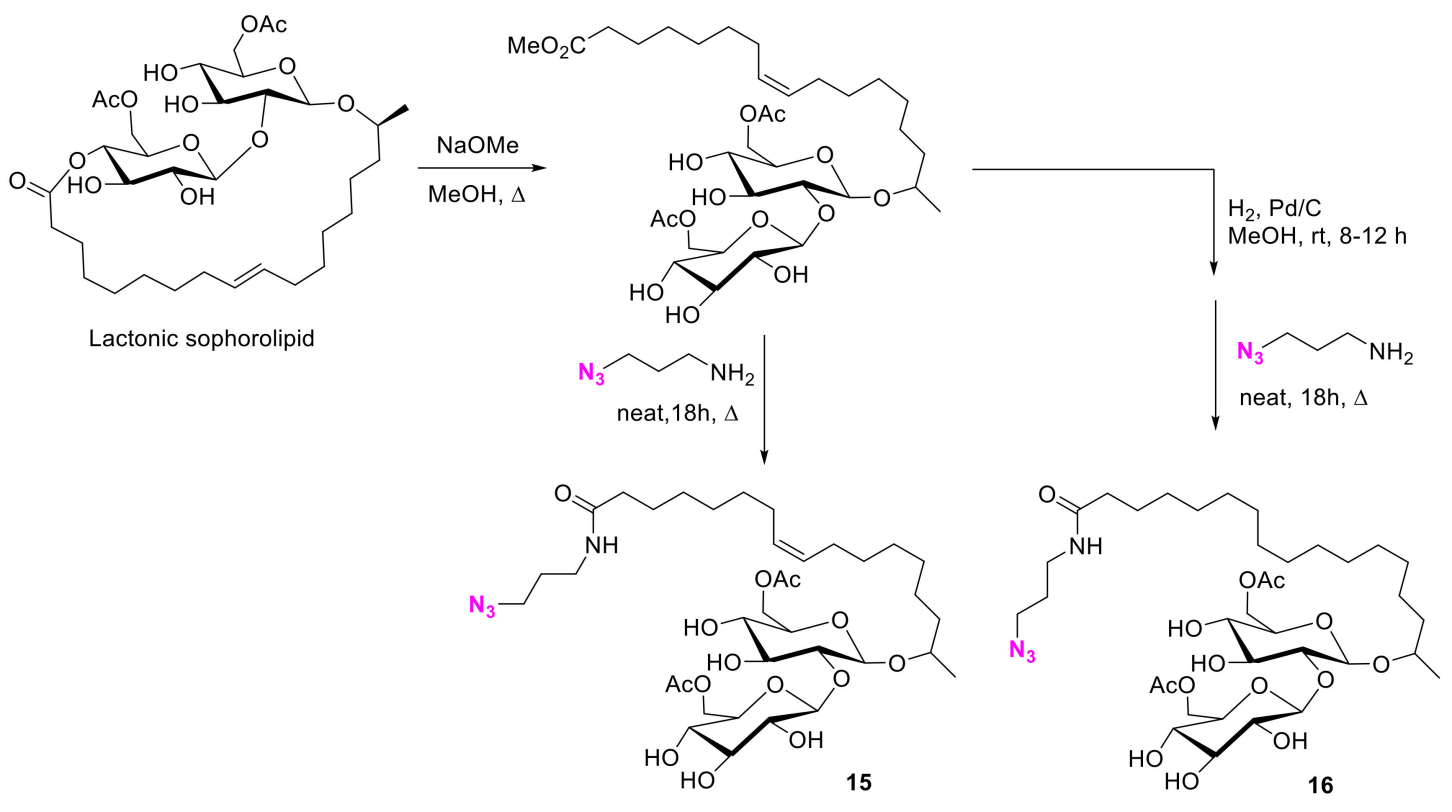
Synthesis of glycolipid surfactants’ moieties **15** and **16**.

Other research groups searching for bio-based molecules for supramolecular self-assembly into functional materials for electro-optical applications were able to conjugate, through the CuAAC approach, glycolipid surfactants’ moieties such as **15** and **16** with 5,10,15,20-tetrakis(4-ethynylphenyl)porphyrin **P1**-**Zn**. These azide glycolipid surfactants (**15** and **16**) were prepared by ring-opening of lactonic sophorolipids followed by reaction with 3-azidopropylamine under neat reaction conditions (**15**) (Scheme 17). Since the double bond confers a certain degree of rigidity that influences the self-assembly of the corresponding functional material, the catalytic hydrogenation step was performed before the reaction with 3-azidopropylamine affording derivative **16** with a good yield (>60%) [41].

**Scheme 18:**
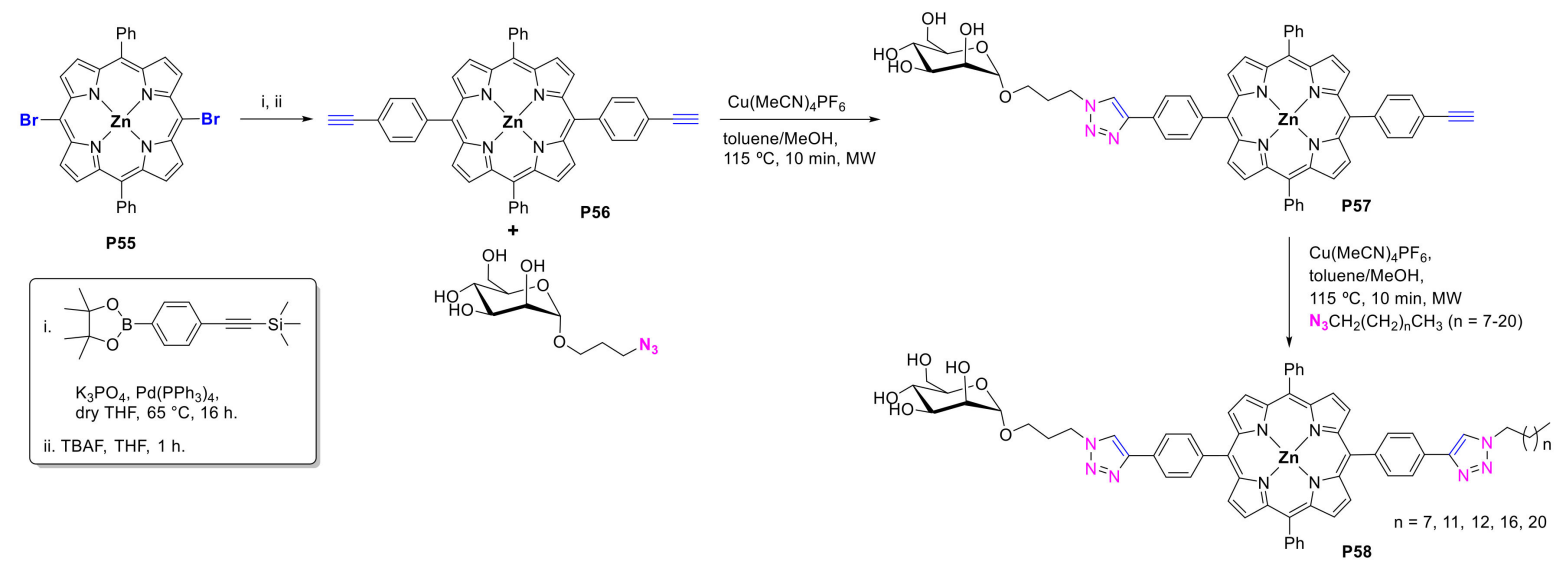
Synthetic approach to prepare the amphiphilic porphyrin derivatives **P58** to be used in PDT.

Senge and co-workers used the bis(4-ethynylphenyl)porphyrin derivative **P56** to prepare amphiphilic target molecules **P58** to be used in PDT (Scheme 18) [42]. In this study, the authors used the 3-azidopropyl α-d-mannopyranoside as the carbohydrate component. Starting from 5,15-dibromo-10,20-diphenylporphyrin **P55**, the ethynyl derivative **P56** was prepared with a 72% yield via a Suzuki cross-coupling reaction using 4-[(trimethylsilyl)ethynyl]phenylboronic acid pinacol ester, K_3_PO_4_, Pd(PPh_3_)_4_ in THF at 65 °C for 16 h (Scheme 18). The click reaction between a large excess of the ethynyl porphyrin **P56** and the azide sugar compound afforded the monosubstituted derivative **P57** after 10 min at 115 °C under microwave irradiation. Following a similar approach, the second ethynyl group was then grafted with different azidoalkyl chains N_3_CH_2_(CH_2_)_n_CH_3_ (*n* = 7–20). Two of the prepared compound **P58** (*n* = 7 and 16) exhibited singlet oxygen production similar to Foscan.

**Figure 5:**
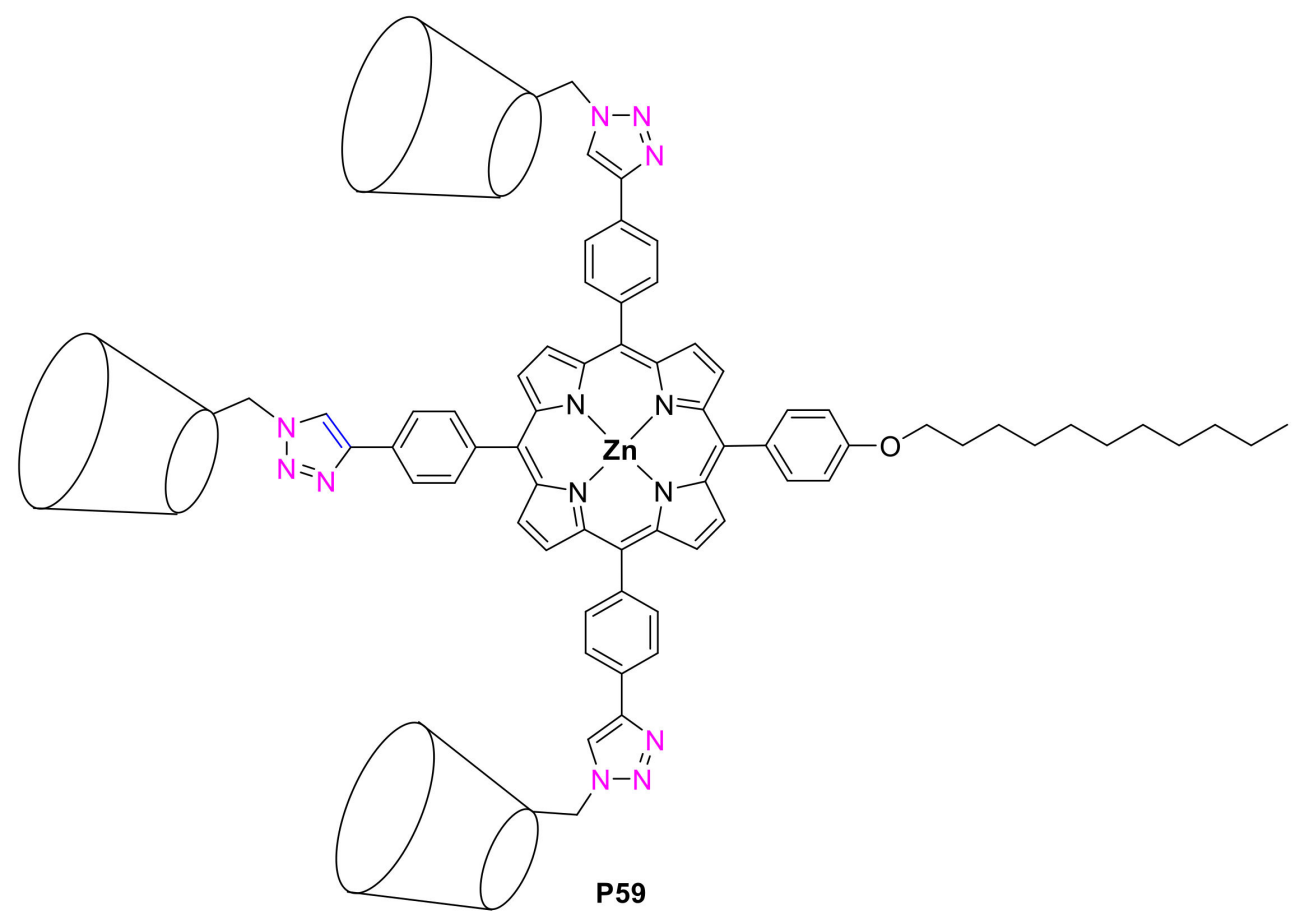
Structure of the amphiphilic porphyrin-cyclodextrin conjugate **P59**.

The CuAAC approach also allowed the efficient preparation (90% yield) of other amphiphilic porphyrin conjugates such as the porphyrin-cyclodextrin conjugate **P59** (Figure 5) [43]. The conjugation between the 6-deoxy-6-azidopermethyl-β-cyclodextrin and the *meso*-tris(ethynylphenyl)porphyrin derivative was performed in THF under stirring in the presence of copper(II) sulfate and sodium ascorbate in water for 48 h at 60 °C under an argon atmosphere. The obtained amphiphilic porphyrin-cyclodextrin conjugate **P59** was used to prepare supramolecular nanoarchitectures to control drug delivery.

**Figure 6:**
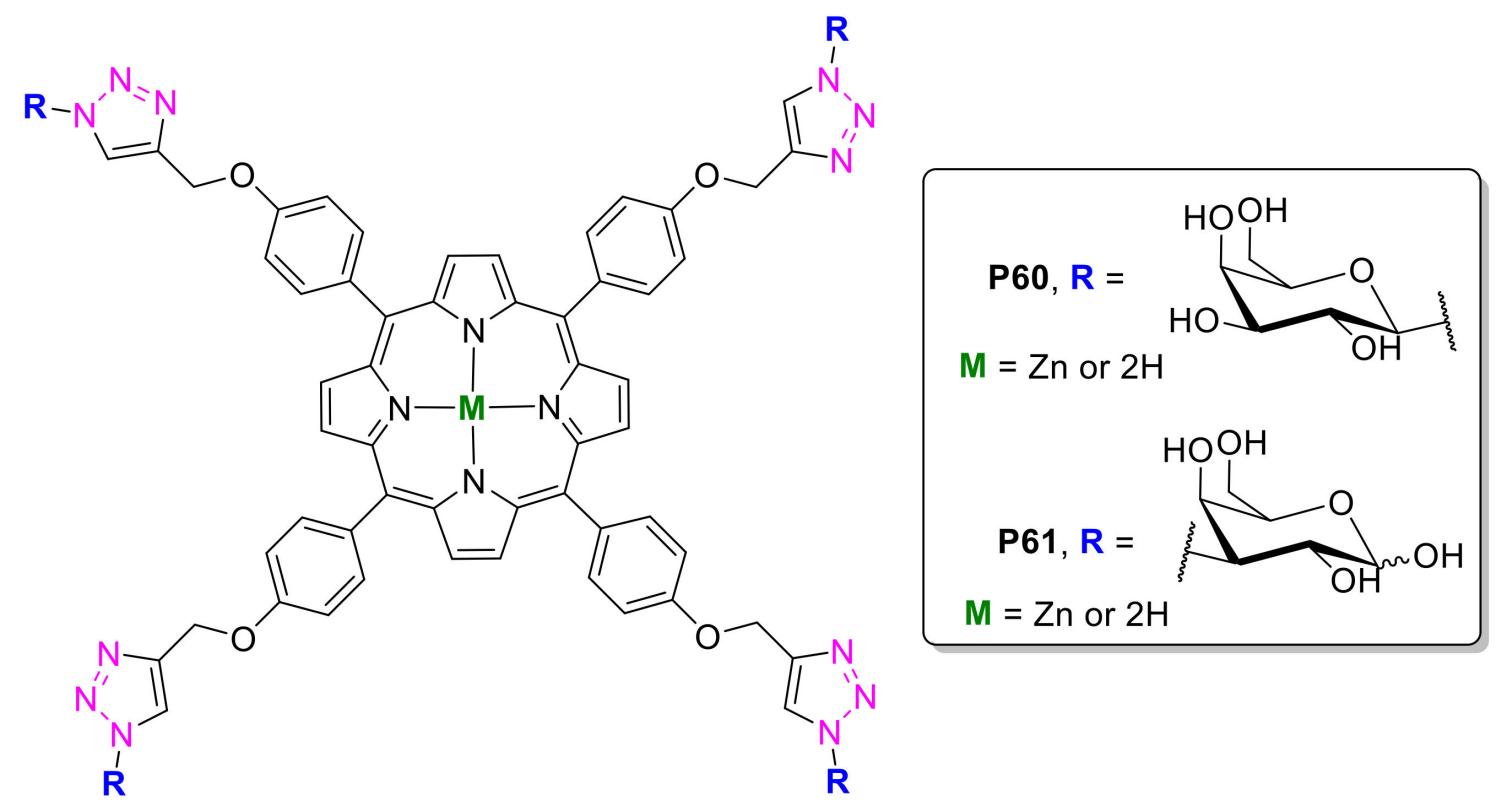
Structures of porphyrin–carbohydrate conjugates **P60** and **P61**.

Drain and co-workers also reported the synthesis of several porphyrin derivatives bearing carbohydrate moieties [44]. These authors prepared carbon-1 and carbon-3-galactosyl porphyrin conjugates **P60** and **P61** (M = 2H or Zn) under CuAAC conditions and tested them towards four tumoral cell lines (monolayers or spheroids) containing high expression of galactin-1 protein (Figure 6). The results pointed out that the uptake of the cabon-3 galactose porphyrin derivatives (**P61**) is higher than the carbon-1 galactose porphyrin derivatives (**P60**) probably due to the higher hydrophilicity and the binding recognition domain of galectin-1 protein by the available hydroxyl group at carbon-1 [44].

Using the same alkynyl porphyrinic precursor **P26**, Rajakumar and co-workers [45] and Rivera and co-workers [46] prepared different amphiphilic dendrimers by a convergent synthetic strategy also using click chemistry. The antitumoral activity of some dendrimers was evaluated towards the MIA PaCa-2 cell line and showed that cell growth inhibition increased along the dendritic generation [45]. Other dendrimers exhibited ability to form *J*-aggregates by increasing the polarity of the solvent but such aggregation can be destroyed by enhancing the temperature [46].

**Scheme 19:**
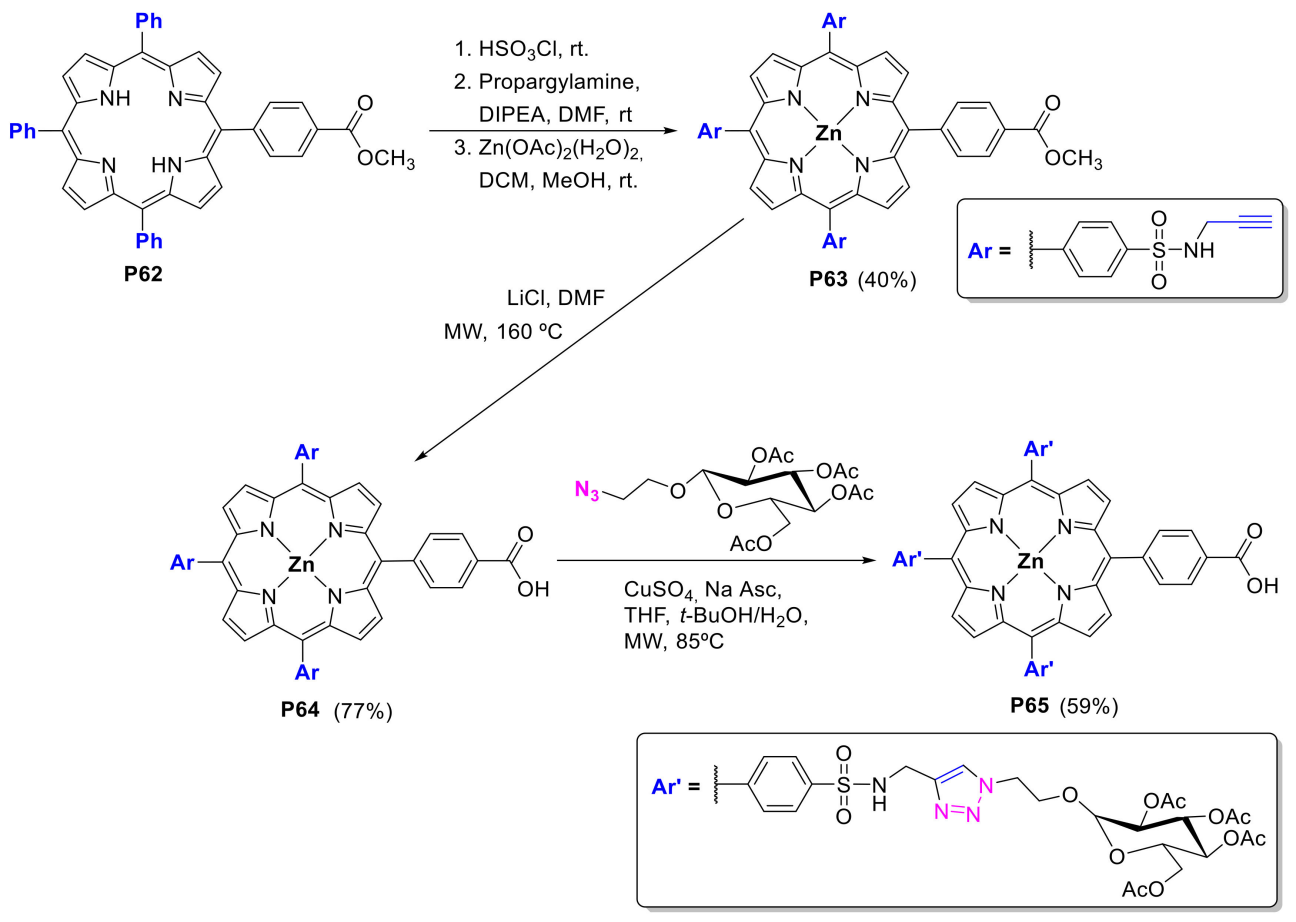
Synthetic strategy used in the preparation of **P65**.

Nilsson and co-workers selected the strategy shown in Scheme 19 to prepare the tri-glucosylated-porphyrin conjugate **P65** and analogues (the click step was extended also to galactose and *N*-acetyl glucosamine azides) to be used as theranostic agents [47,48]. For that, the asymmetric porphyrin precursor **P62** was treated successively with chlorosulfonic acid, propargylamine in the presence of *N*,*N*-diisopropylethylamine (DIPEA) and Zn(II) acetate in order to gain porphyrin **P63** at a total yield of 40%. The click reaction occurred under microwave irradiation after 5 min at 85 °C in a sealed vessel at a 59% yield. After the transformation of the chlorosulfonic groups into the corresponding alkynyl sulfonamide function a new click approach was used to react the product with 2-azidoethyl 2-fluoro-2-deoxy-β-glucopyranoside (structure not shown) to prepare a multimodal imaging and PDT agent. All glycosylated derivatives were deacetylated yielding the hydrophilic glycosyl-porphyrin conjugates in very good yields after treatment with sodium hydroxide, methanol and water at room temperature. The target product has demonstrated significant selectivity towards melanoma cells [48].

#### 2.1.3. Immobilization of Porphyrins in Different Matrixes

Due to the interesting photophysical and biological properties displayed by porphyrin derivatives, they have been incorporated/embedded in or covalently linked to several organic and inorganic supports. Among several coupling approaches, the CuAAC reaction conditions were used successfully to link natural or synthetic porphyrin derivatives to cellulose fibers, dextran, mesoporous organosilicas, SiO_2_-magnetic nanoparticles or functionalized glass.

**Scheme 20:**
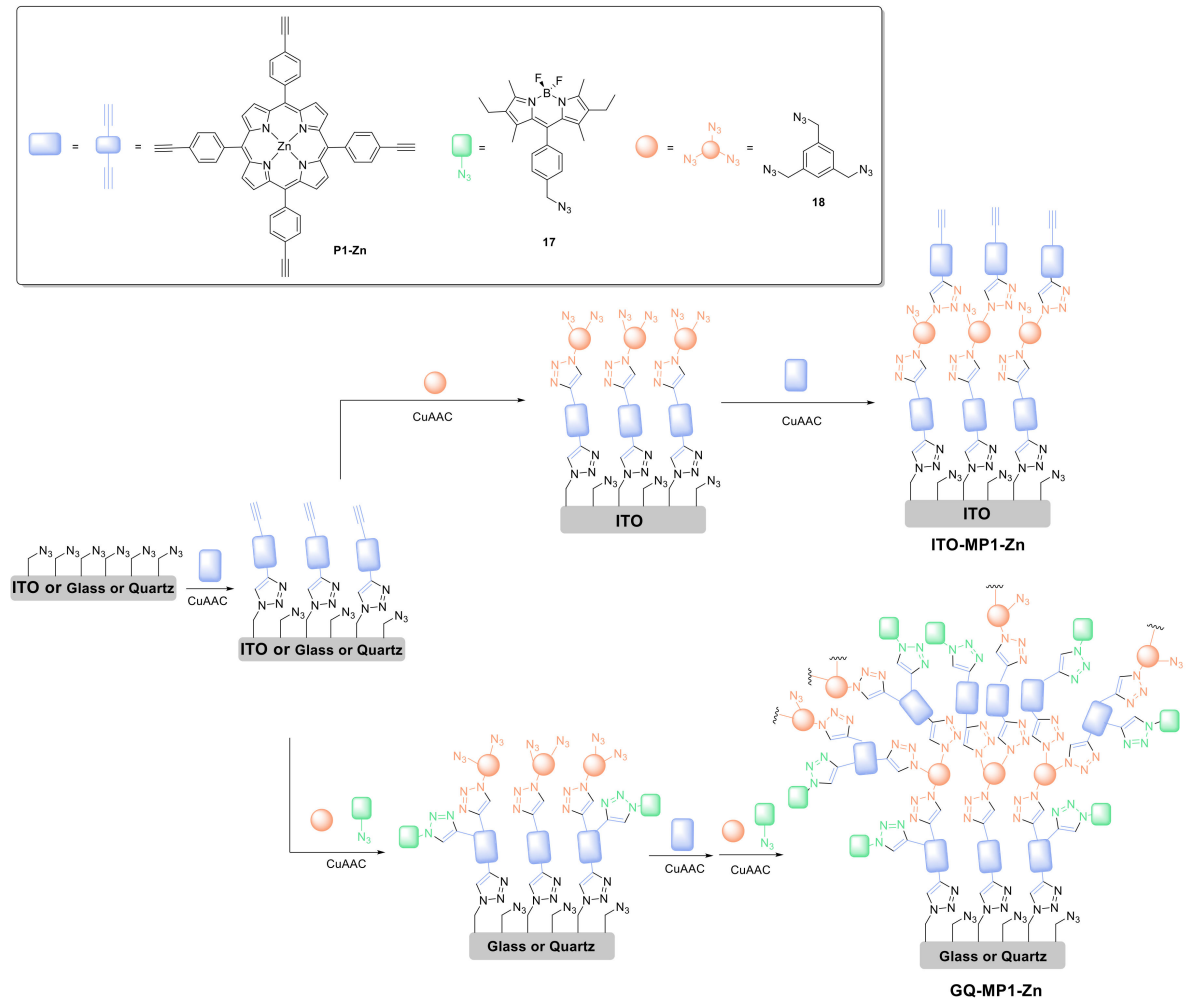
Strategy used to immobilize **P1-Zn**, **17** and/or **18** in glass, quartz or ITO surface by LbL.

For instance, in 2015 Topka and Dinolfo explored the CuAAC approach in combination with the layer-by-layer (LbL) process to assemble over an azide-rich functionalized glass or quartz the alkynyl porphyrin **P1-Zn**, the azide-BODIPY **17** and the tris-azido linker **18** to obtain the multilayer film **GQ-MP1-Zn** (Scheme 20) [49]. The LbL process started with the conjugation of the ethynyl **P1-Zn** with the azide functionalized glass or quartz surface (prepared by anchoring 11-azidoundecyltrimethoxysilane to glass/quartz slides surface exposed to a piranha solution) followed by reaction of the remaining ethynyl moieties with a mixture of azide-BODIPY **17** and the tris-azido linker **18** in order to allow the formation of another porphyrinic layer. In this way, it was possible to add multiple layers and consequently to modulate the optical density through their thickness. In fact, this strategy is very convenient to generate multichromogenic (donor-acceptor) energy transfer assemblies to develop full spectral light harvesting materials for DSSC devices [49]. The same research group, by following the LbL process and through a click approach, prepared other porphyrin-based molecular multilayer films (**ITO-MP1-Zn**) on indium tin oxide (**ITO**) electrodes for photovoltaic and photogalvanic devices [50].

**Scheme 21:**
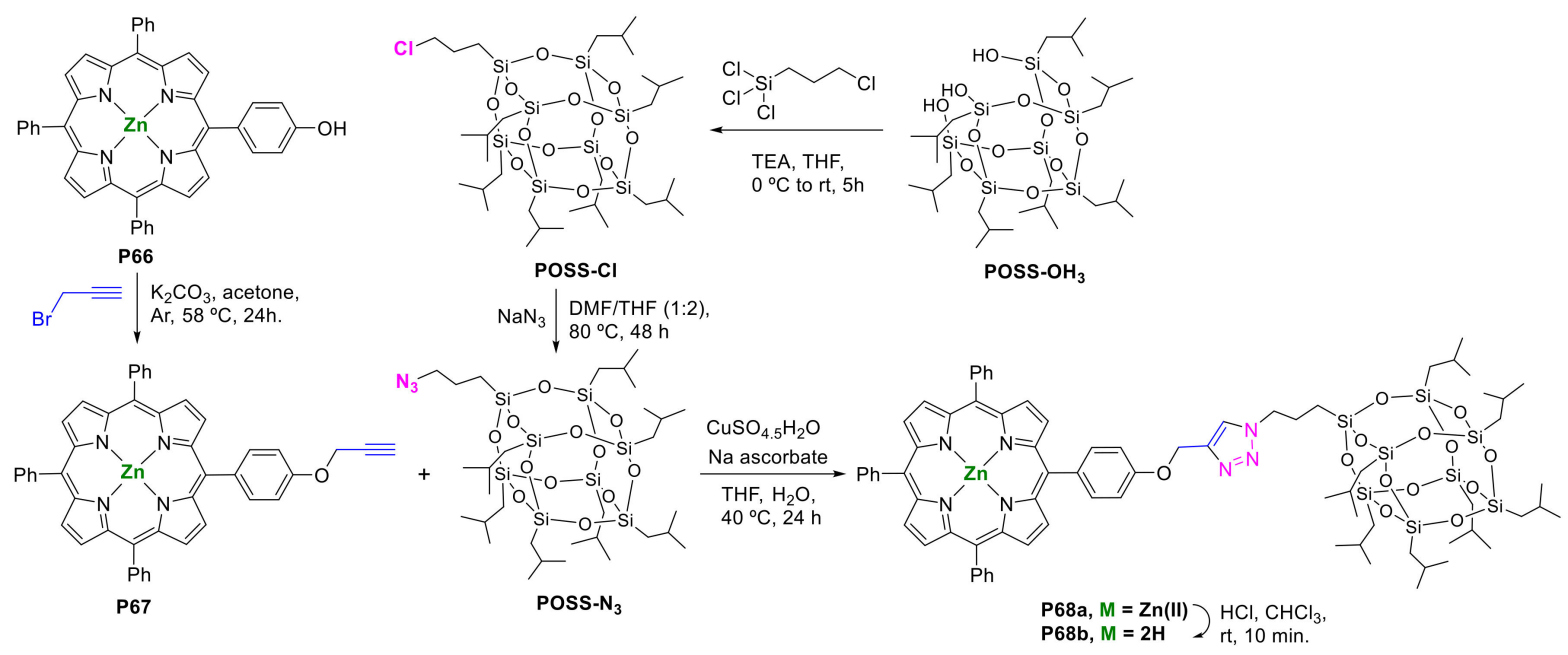
Preparation of organic-inorganic porphyrin-POSS hybrids **P68**.

Zhang and co-workers described the preparation of organic-inorganic hybrid porphyrin derivatives with a polyhedral oligomeric silsesquioxane (POSS) [51,52]. The authors followed two different synthetic approaches. Firstly, POSS functionalized with an azido group (POSS-N_3_) [53] was directly attached to the porphyrin-alkyne Zn(II) complex **P67,** under classical click chemistry conditions, with the formation of the expected triazole ring. Zn(II) porphyrin-POSS hybrid **P68a** was obtained at a 49% yield after 24 h at 40 °C (Scheme 21) [51].

The corresponding free-base derivative **P68b** (96% yield) was obtained after the treatment of hybrid **34a** with conc. HCl in chloroform and their self-assembly behavior was explored. The authors found that different morphologies, such as core-shell spherical micelles and ordered square sheets, can be obtained depending on the polarity of the solvent or solvent mixtures used [51].

**Figure 7:**
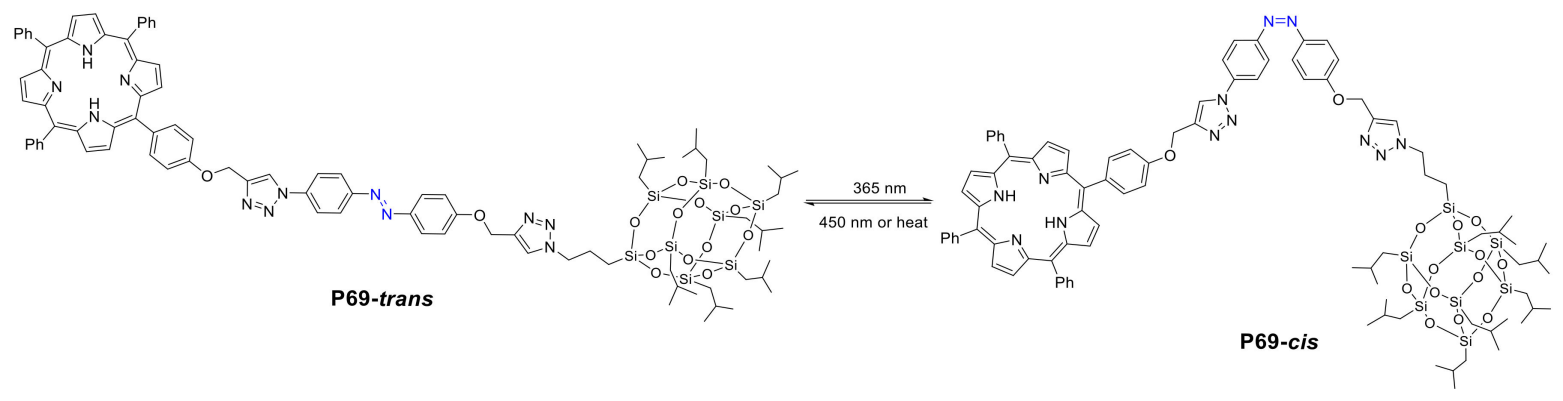
*trans* and *cis* configurations of **P69** switching induced by light or heat.

The same group prepared the photosensitive organic-inorganic porphyrin-POSS hybrid **P69** with an azobenzene bridge (Figure 7) [52]. The synthetic route involved the reaction of porphyrin derivative **P67** with 4-azido-4´-hydroxyazobenzene affording the formation of the porphyrin-azobenzene derivative linked by 1,2,3-triazole rings (50% yield). Through a substitution reaction with 3-bromoprop-1-yne in the presence of potassium carbonate, this compound afforded the corresponding alkyne derivative that was submitted to a second click chemistry reaction with POSS-N_3_ to obtain the Zn(II) complex of **P69-*trans*** at a 51% yield. The treatment with HCl afforded the free-base derivative **P69-*trans*** in quantitative yield [52].

The azo bridge allowed the change from a *trans* to a *cis* configuration and, in such way, there is a significant reduction in the molecule size. This was performed by irradiation with UV light (350 nm), with visible light (450 nm) or with heating (Figure 7). The supramolecular self-assembly of derivative **P69-*trans***, as observed for compound **P68**, has a strong solvent dependence but additionally it also depends on the configuration. The possibility to modulate the structure by using light presents potentialities for applications in bio-imaging, drug release and catalysis [52].

**Scheme 22:**
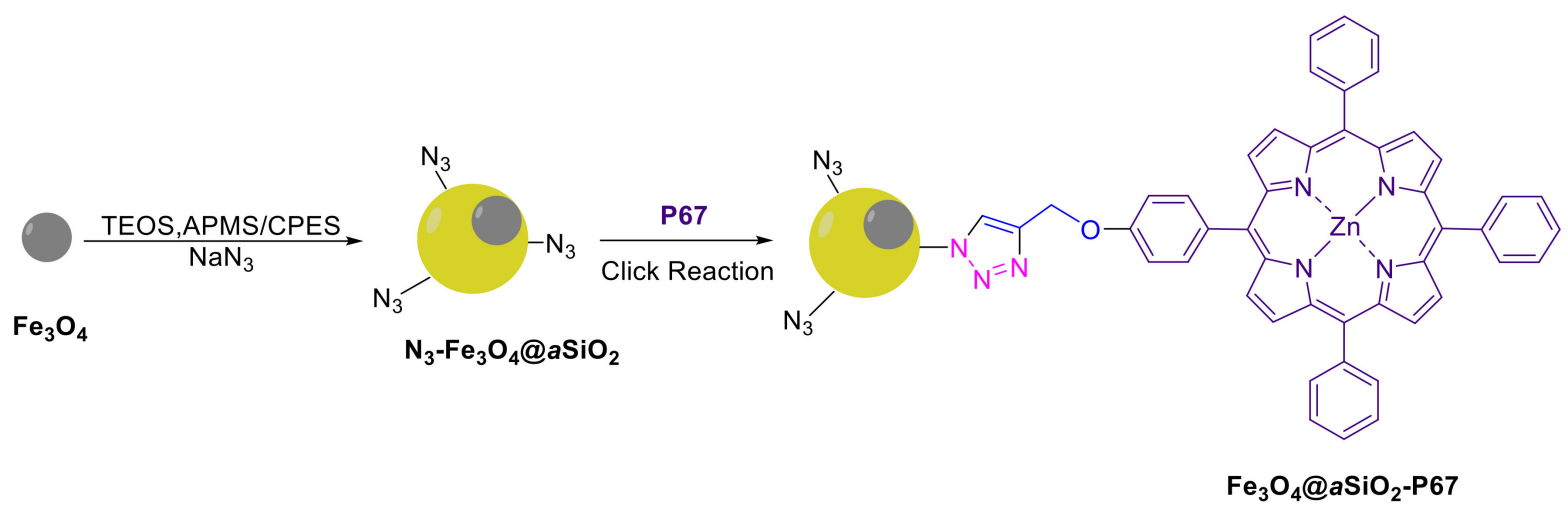
**P67** immobilized in magnetic nanoparticles with the magnetic off-centered core.

Aiming to obtain nanocatalysts able to regulate polymerization kinetics in response to dual external stimuli (light and magnetism), Cai and co-workers have reported the covalent anchorage of the alkynyl Zn(II) complex **P67** into the Fe_3_O_4_@*a*SiO_2_ Janus type nanoparticles (magnetic nanoparticles with the magnetic off-centered core) bearing azide units (Scheme 22). The coupling was performed in the presence of PMEDTA, Cu(II) acetate and sodium ascorbate in DMF at 50 °C for 12 h under an argon atmosphere [54]. The catalyst efficacy of Fe_3_O_4_@*a*SiO_2_-P67 in photo-induced electron transfer reversible addition-fragmentation chain transfer (PET-RAFT) processes was tested using vinylic monomers such as styrene, acrylates or acrylamides and led to the preparation of several homopolymers after green light activation (520 nm, 5 W, 1.3 mW cm^−2^). The results showed that the nanocatalyst was regenerated without significant photodegradation.

**Figure 8:**
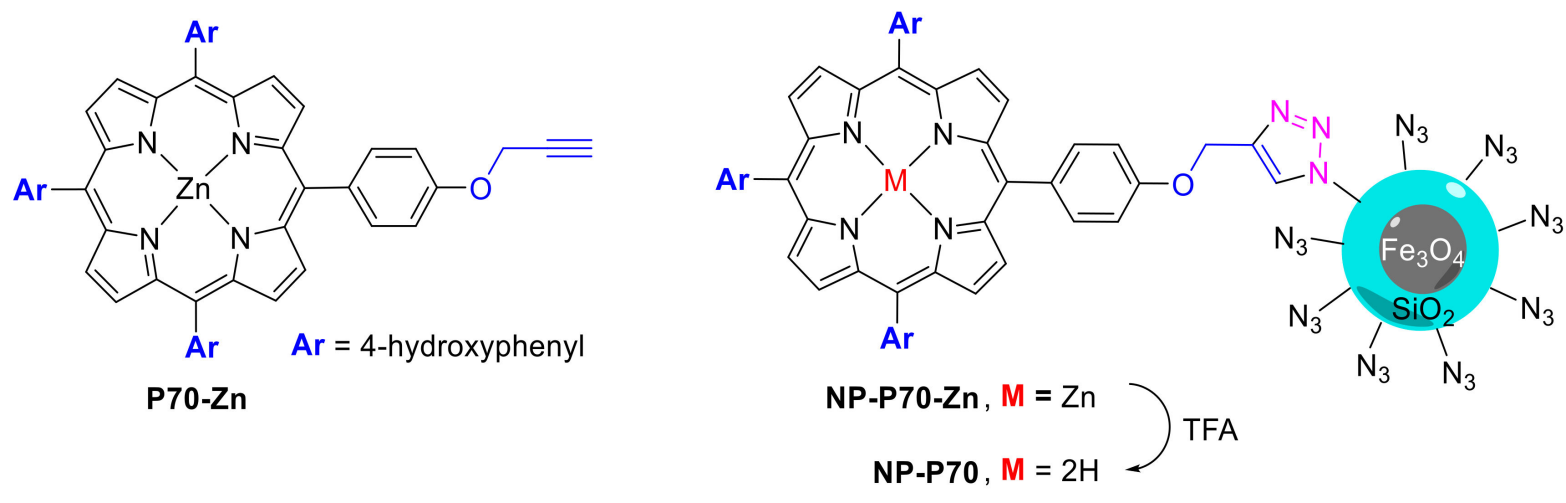
Magnetic nanoparticle **NP-P70** prepared via click approach from porphyrin **P70-Zn**.

Russo and colleagues used a similar approach to covalently immobilize metalloporphyrin **P70-Zn** on the surface of superparamagnetic silica nanoparticles (Fe_3_O_4_@SiO_2_) functionalized with the azide component (Figure 8) [55]. The coupling was conducted in degassed toluene in the presence of CuBr and PMEDTA for 24 h at 40 °C; the obtained **NP-P70-Zn**, after acid treatment with TFA, afforded the nanoparticles **NP-P70** bearing free-base porphyrin units.

**Scheme 23:**
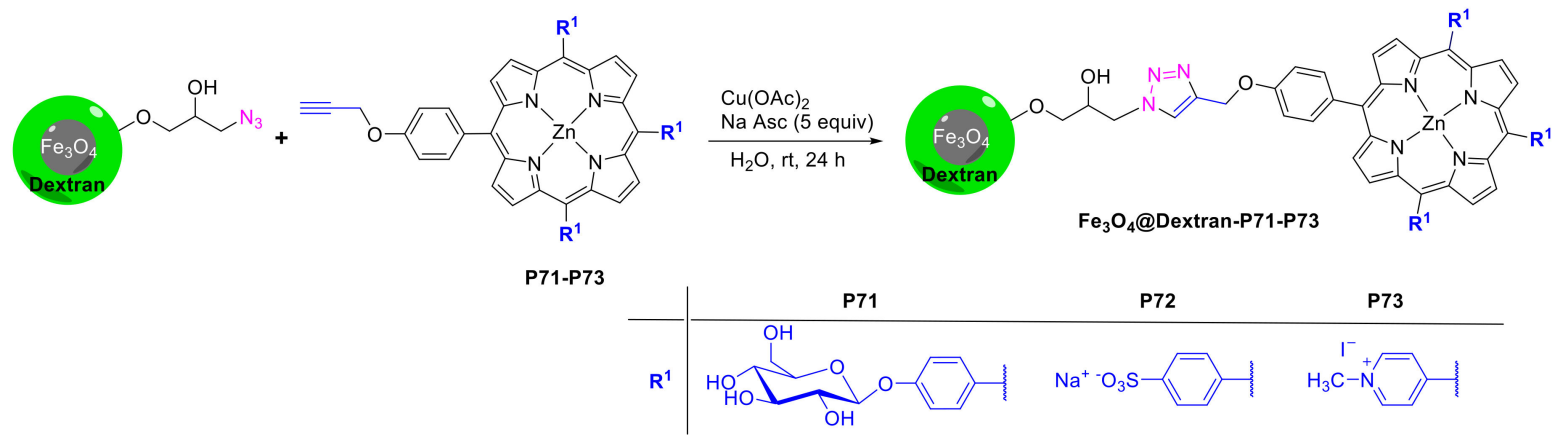
Magnetic nanoparticle coated with native dextran and linked to water-soluble photosensitizers.

The Fe_3_O_4_ magnetic core can also be capped with other shell types such as the polysaccharide dextran, a biocompatible polymer, with high affinity to iron oxide. Sol and co-workers selected Fe_3_O_4_@Dextran nanoparticles to prepare nanoplatforms for delivering photosensitizers to tumoral cells for the PDT approach [56]. The synthesis of the conjugates involved first the functionalization of the dextran surface with epichlorohydrin followed by reaction with sodium azide, in order to functionalize the nanoparticle surface with the required azido functions (Scheme 23). The conjugation with the water-soluble alkynyl porphyrin derivatives **P71**–**P73** took place in aqueous media at room temperature for 24 h in the presence of Cu(II) acetate and sodium ascorbate; the water solubility was conferred by the presence of glucosyl, sulfonate, or pyridinium groups. The graft yields obtained after dialysis (72 h against pure water) varied between 24% and 49%, the best values being found for the anionic and cationic derivatives **P72** (43%) and **P73** (49%). The results showed that the cationic conjugate **Fe_3_O_4_@Dextran-P73** displayed magnetic properties to be used not only as contrast agent for MRI and drug delivery but also in hyperthermia therapy [56].

**Figure 9:**
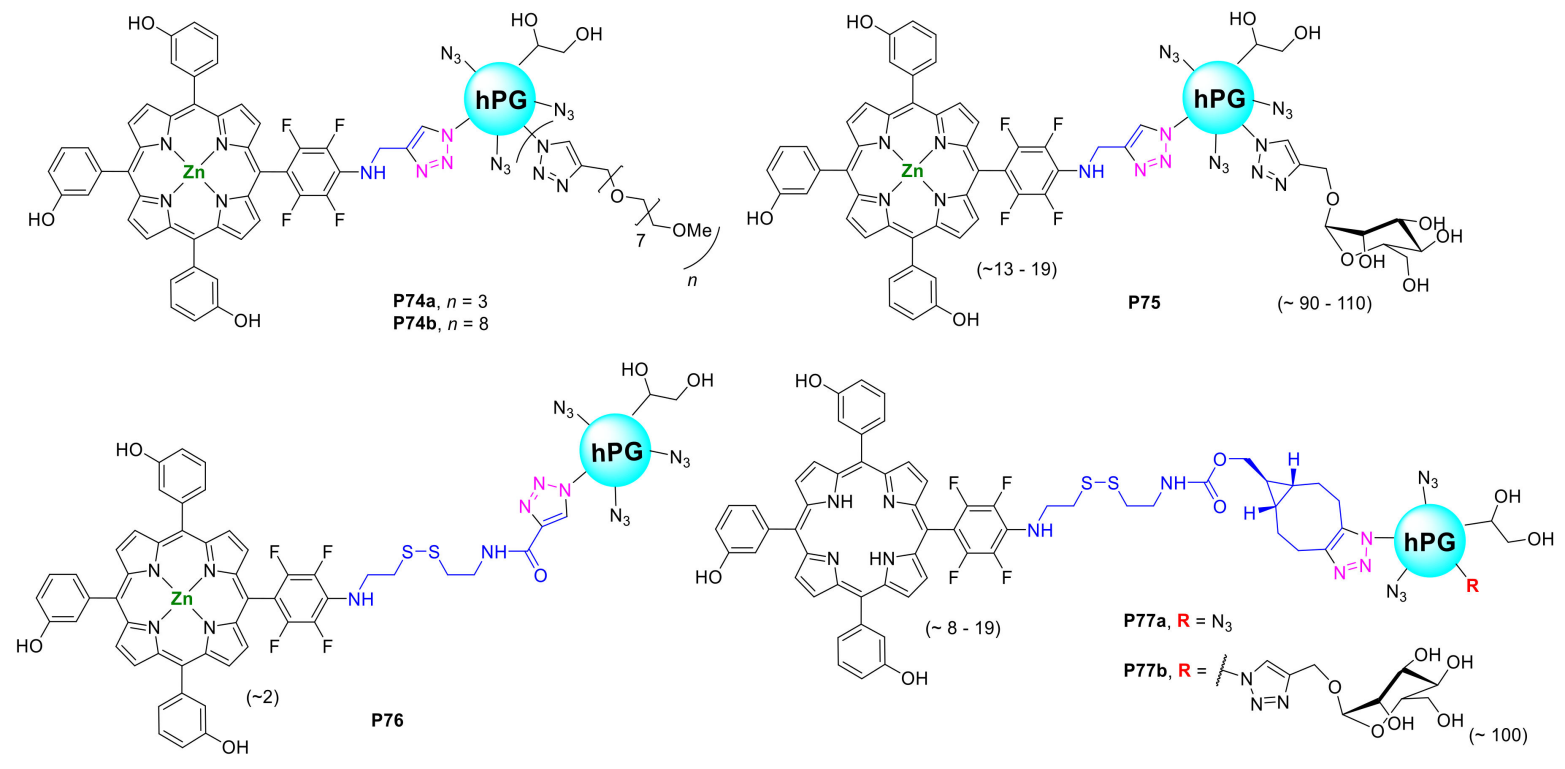
Structure of porphyrin-hPG-PEG conjugates **P74a**,**b**, **P75**, **P76** and **P77**. Numbers in brackets give the approximate loading per polymer with porphyrin molecules and mannose groups, respectively.

The alkynyl-porphyrin derivative **P4** was immobilized in hyperbranched polyglycerol (hPG) functionalized with azide units affording the corresponding porphyrin-hPG conjugates under CuAAC conditions. To improve the solubility in water, some of the azide units at the hPG core were then functionalized with methoxy poly(ethylene glycol) (mPEG)-propargyl affording the porphyrin-hPG-PEG conjugates **P74a**,**b** (Figure 9) as biocompatible drug nanocarrier systems [20].

The authors extended this approach to obtain porphyrin-hPG conjugates **P75**, **P76** and **P77** with different linkers and mannose content and demonstrated that some of them exhibit strong phototoxicity against *S. aureus* and several tumor cell lines at micromolar concentration range [57,58].

**Scheme 24:**
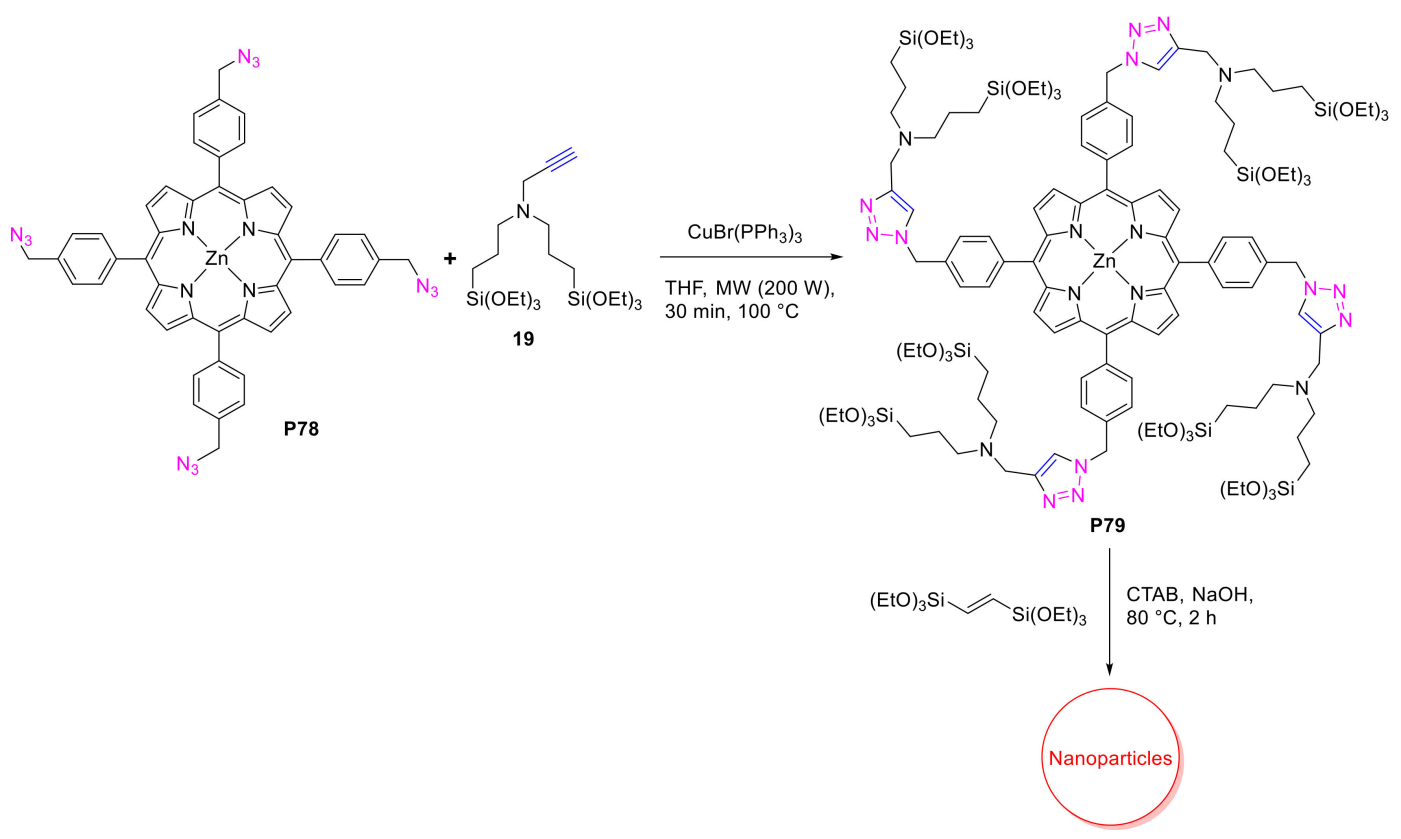
Synthesis of mesoporous organosilica nanoparticles containing Zn(II) porphyrin moieties.

Durand and co-workers also used porphyrin **P78** to synthesize the derivative **P79**, which was used to produce mesoporous organosilica nanoparticles containing Zn(II) porphyrin moieties [59]. The authors obtained the sol-gel precursor **P79**, featuring eight triethoxysilyl groups, through the CuAAC reaction of **P78** with *N*,*N*-bis(3-triethoxysilylpropyl)prop-2-ynylamine (**19**) in dry THF in the presence of CuBr(PPh_3_)_3_ and under MW irradiation (Scheme 24). The nanoparticles were prepared by co-condensation of porphyrin **P79** with bis(triethoxysilyl)ethene in water at 80 °C, under basic catalysis, and using cetyltrimethylammonium bromide as the structure-directing agent. The nanoparticles revealed to be two-photon-sensitive and their two-photon imaging capacity was assessed in MCF-7 breast cancer cells. Also, owing to their high porosity, the nanoparticles were applied as drug nanocarriers. Nanoparticles were loaded with doxorubicin (drug loading of 20 wt%) and it was observed that the release of doxorubicin was pH dependent. The authors concluded that these doxorubicin-loaded organosilica nanoparticles were very efficient at inducing MCF-7 breast cancer cell killing, showing promising potential for theranostic applications.

**Scheme 25:**
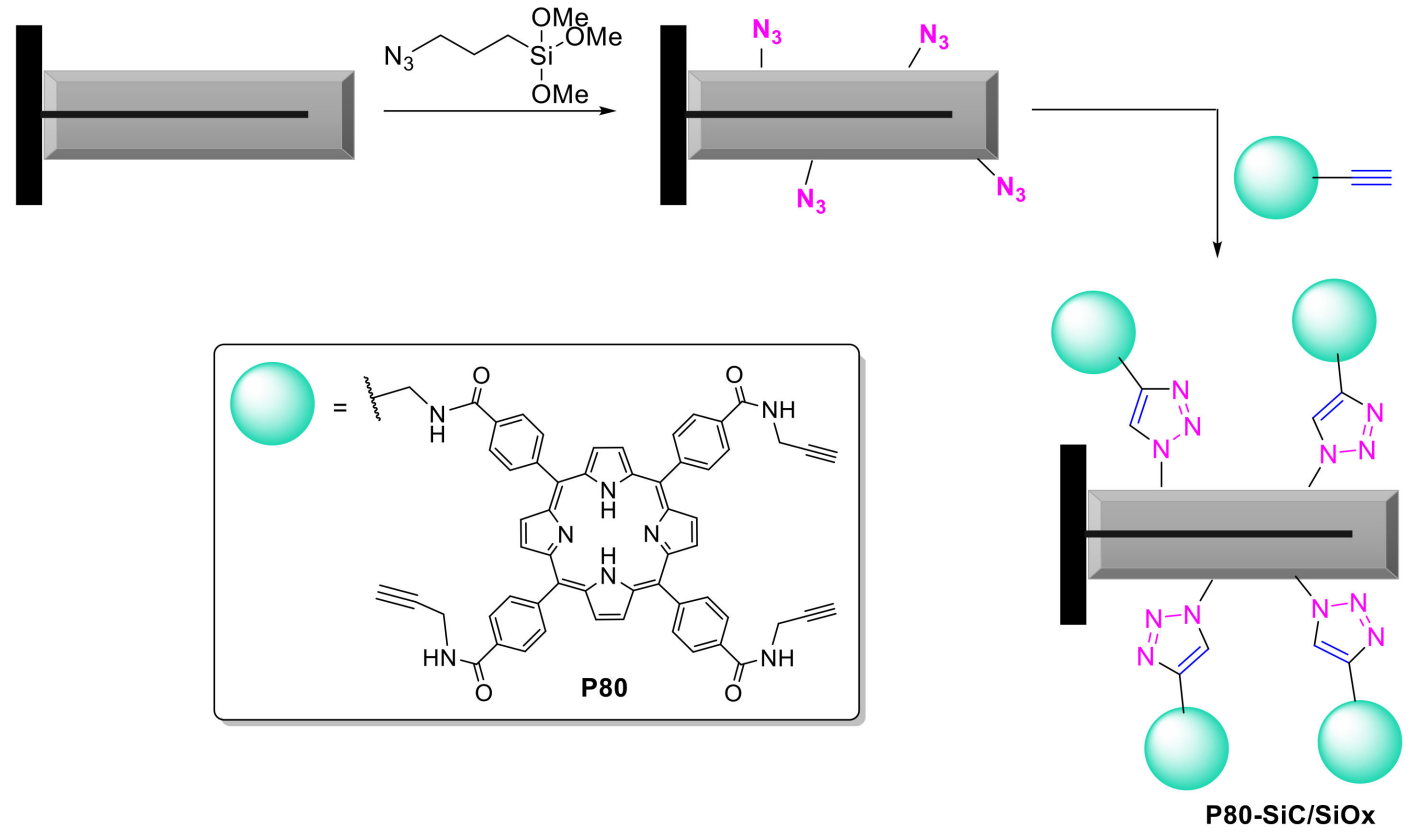
Preparation of **P80-SiC/SiO_x_** nanowires via click chemistry.

Salvatini and co-workers also selected the click approach to link covalently the alkynyl porphyrin **P80** with SiC/SiO_x_ nanowires functionalized with azide units (Scheme 25) [60]. The experimental design was based on the condensation of the nanowires with 3-azidopropyltrimethoxysilane to provide the required azido groups (N_3_-SiC/SiO_x_) at their surface. The alkynyl derivative **P80** was prepared at a 60% yield by reacting 5,10,15,20-tetrakis(4-carboxyphenyl)porphyrin with propargylamine in the presence of EDC and HOBt in DMF for 24 h (Scheme 25). In order to avoid the coordination of the free-base porphyrin with copper(II), the cycloaddition was performed in DMSO using thermal conditions (160 °C for 24 h). The **P80-SiC/SiO_x_** nanowires linked covalently to the porphyrin produced singlet oxygen efficiently after a low dose (0.4-2 Gy) of X-ray irradiation; also, a reduction in 75% of lung adenocarcinoma cells viability was observed after 12 days.

**Scheme 26:**
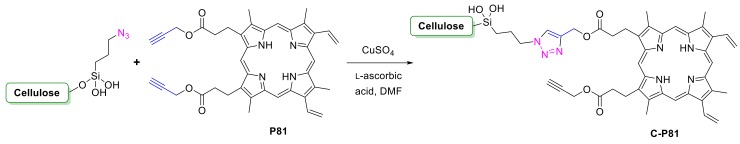
Immobilization of alkynyl-derived protoporphyrin IX **P81** on modified cellulose II matrix.

In 2019, lyocell fibers (a cellulose II matrix), after being submitted to a silanization process with (3-azidopropyl)triethoxysilane, were used to immobilize the alkynyl-derived protoporphyrin IX (**P81**) in order to produce photo-bactericidal materials **C-P81** [61]. The coupling was performed in DMF in the presence of copper(II) sulphate and ascorbic acid for 72 h at room temperature (Scheme 26). The photobactericidal properties of the new materials were evaluated towards two Gram-positive bacterial strains (*S. aureus* and *B. subtilis*) and have demonstrated significant activity against these two strains upon light activation [61].

**Scheme 27:**
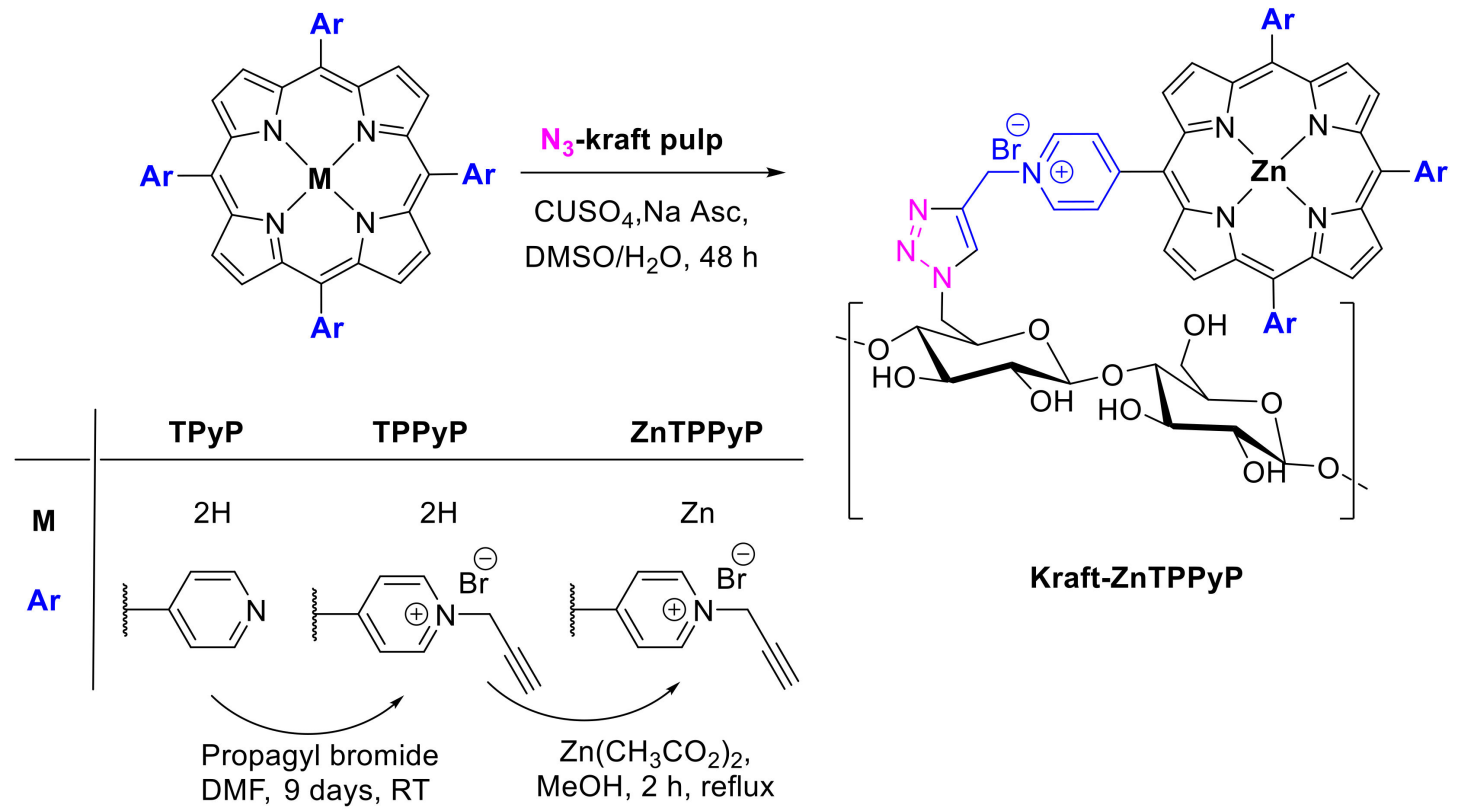
Cationic porphyrin derivative **Kraft-ZnTPPyP** and its immobilization into the kraft pulp material.

Other azide cellulose-derived fibers (kraft pulp) were used to covalently link cationic porphyrin derivatives to be tested as photobactericidal materials (Scheme 27) [62]. It is well-known that cationic porphyrin derivatives exhibit a great antibacterial activity in Gram-positive and Gram-negative bacteria strains independently of their antibiotic resistance and without development of resistance [63,64,65,66]. Thus, pulp kraft was previously tosylated by reaction with an excess of tosyl chloride in triethylamine at room temperature for 96 h; the obtained material was then treated with sodium azide in DMF for 96 h at 50 °C, thus being transformed into the azide kraft pulp (N_3_-kraft pulp) derivative [62]. The propargylated porphyrin **ZnTPPyP** was obtained by treating the neutral **TPyP** with an excess of propargyl bromide at room temperature for nine days, followed by the complexation step with Zn(II). The new material **Kraft-ZnTPPyP** was used to photoinactivate the Gram-positive bacterium *S. aureus* and the Gram-negative strains *E. coli* and *Pseudomonas aeruginosa*. The **Kraft-ZnTPPyP** showed bactericidal activity in the dark as well as photobactericidal effect under white light. However, it did not provide any bactericidal effect on Gram-negative strains even under white light irradiation.

### 2.2. Porphyrins Bearing Azido Groups

#### 2.2.1. *meso*-(*p*-Azidophenyl)porphyrins

**Scheme 28:**
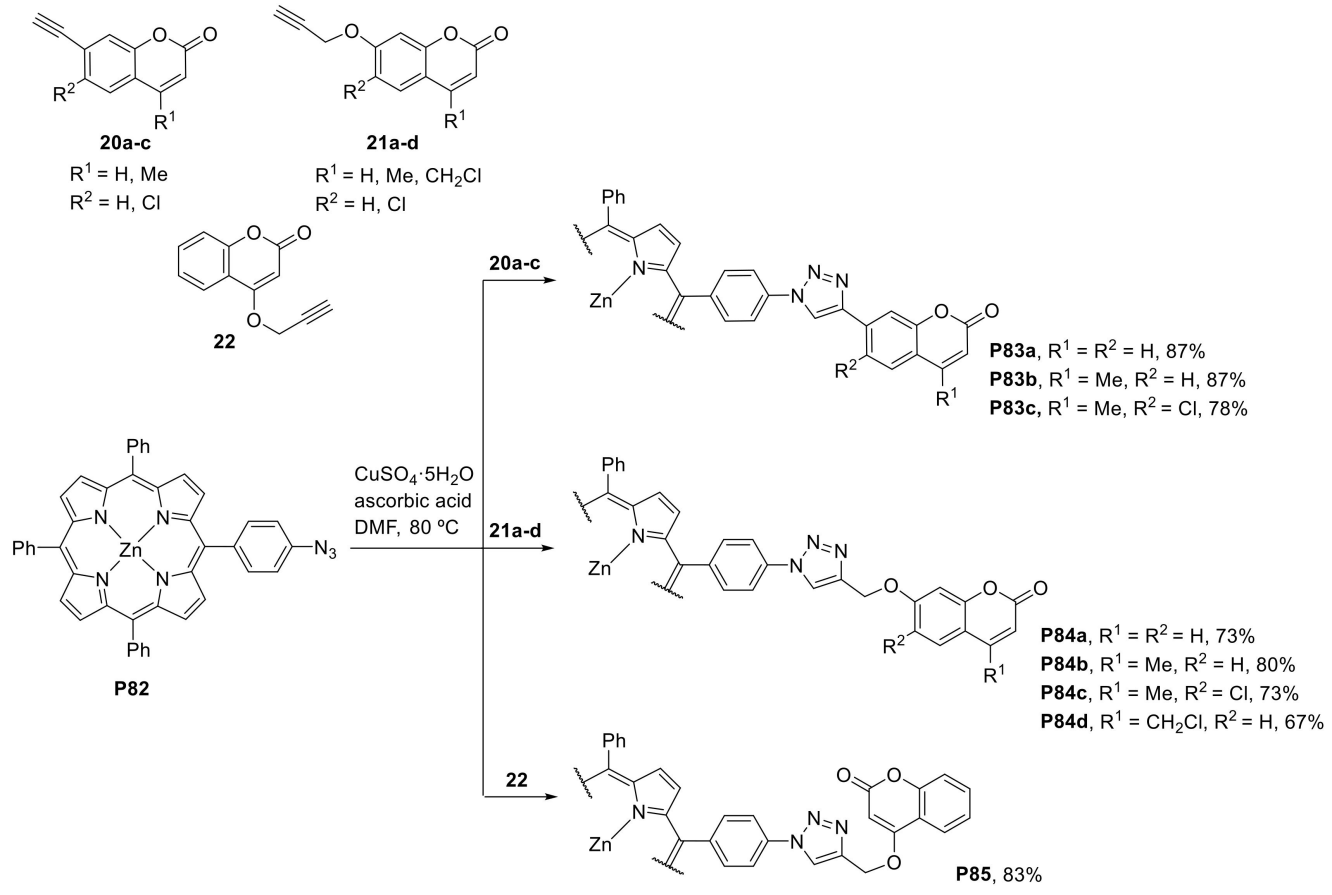
Synthesis of Zn(II) *meso*-phenyl-triazole bridged coumarin-porphyrin dyads.

In recent years, several papers reported the copper(I)-catalyzed 1,3-dipolar cycloaddition reaction of 5-(4-azidophenyl)porphyrins with a diversity of acetylene derivatives. An example involved the reaction of Zn(II) 5-(4-azidophenyl)-10,15,20-triphenylporphyrin (**P82**) with the alkyne-substituted coumarins **20**–**22** to give the Zn(II) *meso*-phenyl-triazole bridged coumarin-porphyrin conjugates **P83**–**P85** (Scheme 28) [67]. The corresponding free-bases, obtained by treatment of the dyads with concentrated HCl, were converted into the nickel(II) complexes by reaction with nickel(II) acetate in chloroform-acetic acid. Photophysical studies of the Zn(II) conjugates revealed a significant intramolecular energy transfer between both units.

**Scheme 29:**
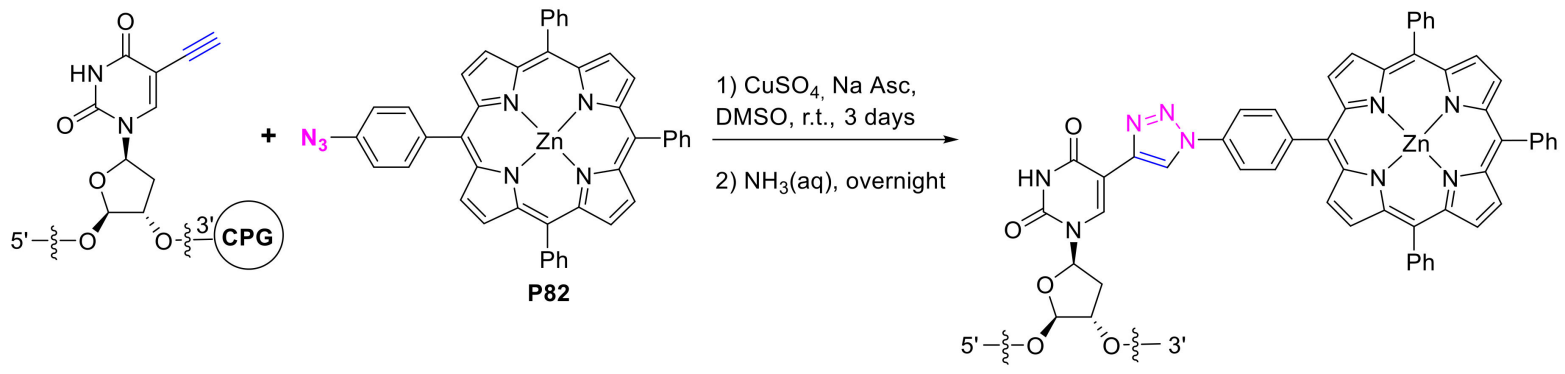
Synthesis of porphyrin-DNA conjugates.

The 5-(4-azidophenyl)porphyrin **P82** was also used to form porphyrin-DNA conjugates [68]. The conjugates were prepared by solid-phase click reaction between the azide **P82** and oligodeoxynucleotides with an ethynyl group on controlled pore glass support (Scheme 29). Porphyrin-DNA duplexes and a dimer of porphyrin were created by hybridization with the appropriate complementary DNA strands. Porphyrin-DNA modified electrodes constructed by a self-assembled DNA monolayer with the porphyrin units on the gold electrode showed a photocurrent response upon light irradiation.

**Scheme 30:**
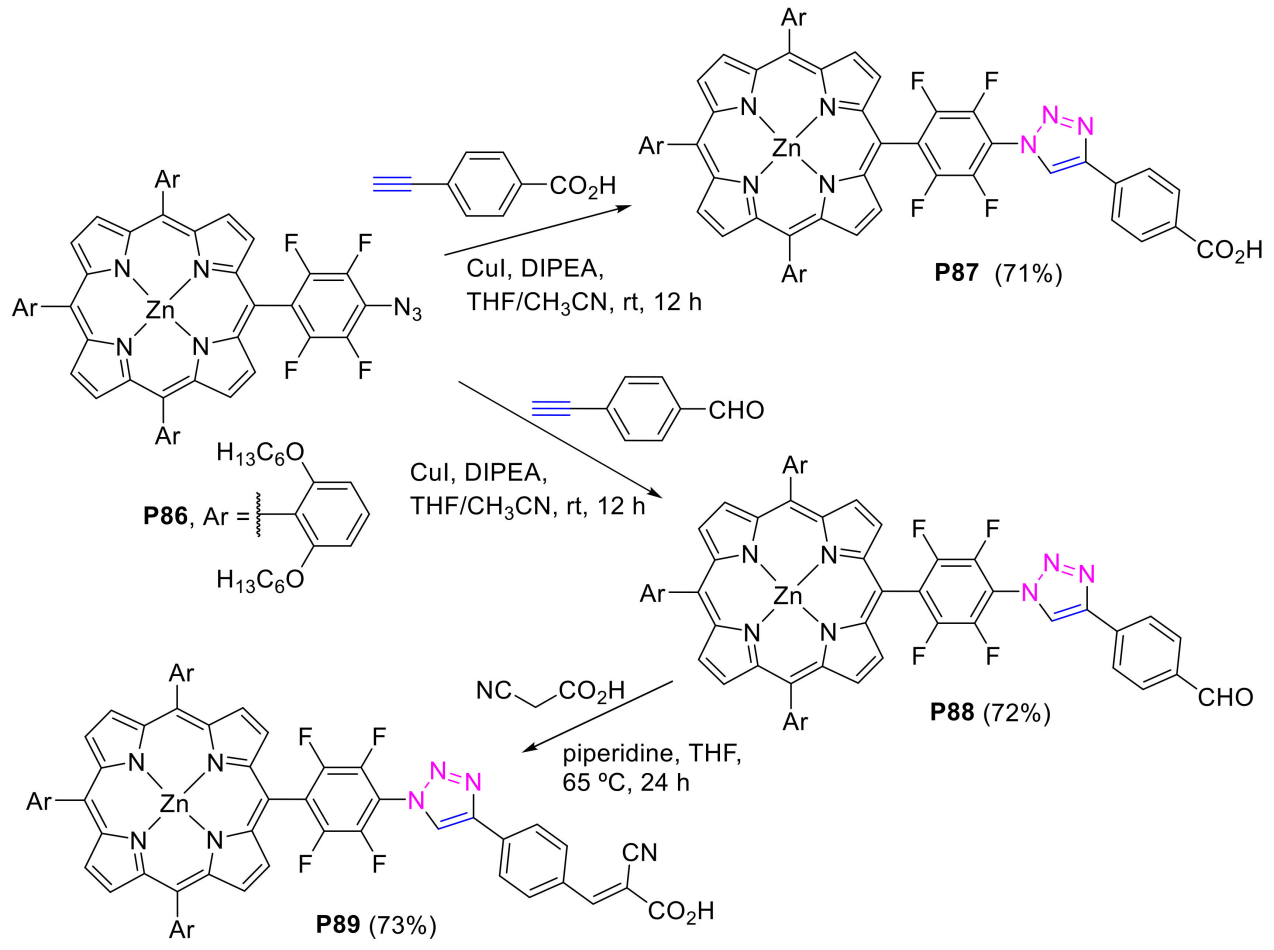
Synthesis of porphyrin–triazole conjugates incorporating carboxyl groups.

Other examples of click chemistry using 5-(4-azidophenyl)porphyrins to synthesize new sensitizers for dye-sensitized solar cells (DSSCs) include the synthesis of the porphyrin-triazole conjugates **P87** and **P89** that incorporate carboxyl groups as anchoring groups (Scheme 30) [69]. DSSCs fabricated with dyes **P87** and **P89** showed better efficiency (eight and four times better, respectively) when compared to reference compounds without the triazole ring. It was concluded that the carboxylate unit, for this type of compounds, is a more efficient anchoring group than the cyanoacrylate unit.

**Scheme 31:**
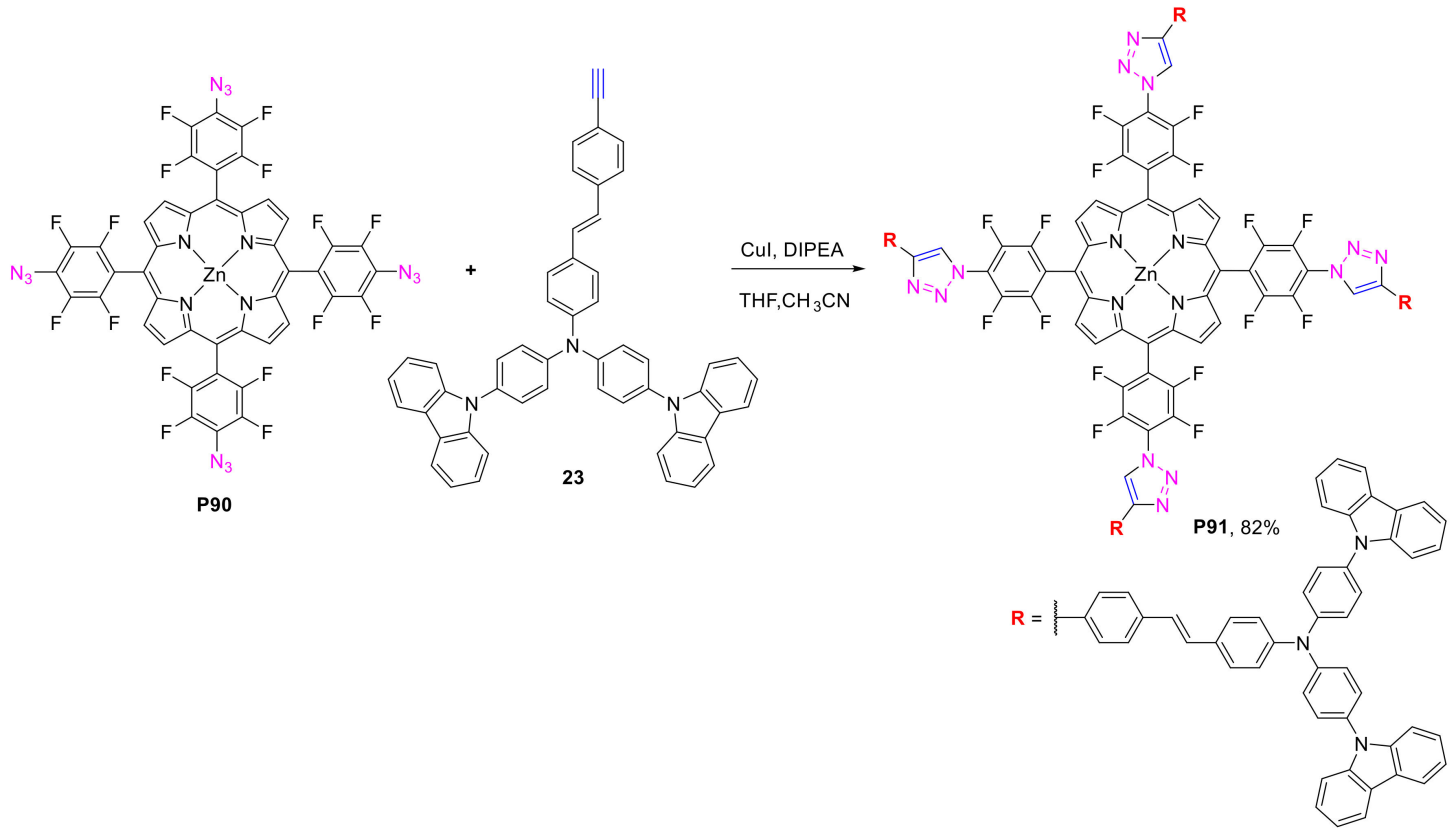
Synthesis of porphyrin-triazole conjugates **P91** incorporating bis(carbazolyl)triphenylamine groups.

Durantini and co-workers reported the synthesis of a cruciform dendrimer, which bears a nucleus of Zn(II) porphyrin substituted at the *meso* positions by four bis(carbazolyl)triphenylamine units linked by triazole rings [70]. The new compound **P91** was obtained from the reaction of the *meso*-tetrakis(azidophenyl)porphyrin **P90** and the acetylenic dendron **23** using the CuAAC method (Scheme 31). The electrochemical oxidation of the carbazole groups in **P91** was used to obtain stable and reproducible fully π-conjugate photoactive polymeric films that are highly stable to photobleaching and produce singlet oxygen in both DMF and water. These films revealed to be very efficient in the photodynamic inactivation of *Staphylococcus aureus* and *Escherichia coli* in both planktonic and biofilm forms.

**Scheme 32:**
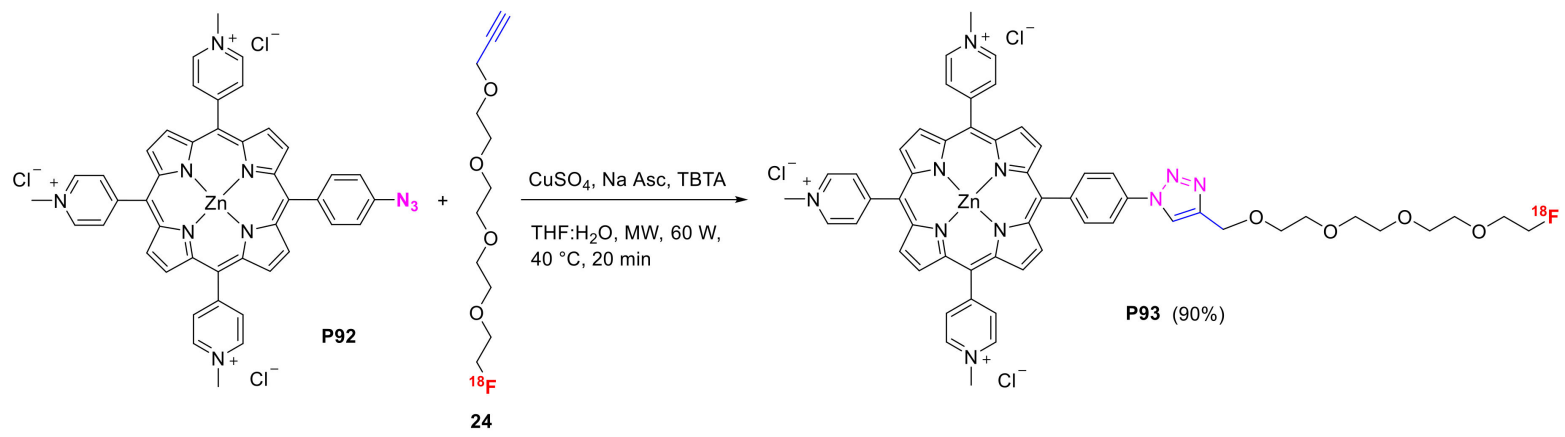
Synthesis of a water-soluble porphyrin radiolabeled with fluorine-18.

Boyle and co-workers reported the synthesis of the first water-soluble porphyrin radiolabeled with fluorine-18 [71]. The tricationic porphyrin **P93** was obtained through click conjugation of the cationic azidophenylporphyrin **P92** with the tetraethoxy PEG derivative **24** (Scheme 32). Biological studies revealed that this porphyrin derivative showed cellular uptake, good photocytotoxicity, and minimal dark toxicity in human adenocarcinoma (HT-29) cells and demonstrated potential properties as a radiotracer in vivo. The new theranostic agent **P93** may be considered a clinically relevant theranostic agent since it integrates the therapeutic selectivity of PDT with the imaging efficacy of positron emission tomography (PET).

#### 2.2.2. *meso*-(*p*-Azidomethylphenyl)porphyrins

**Scheme 33:**
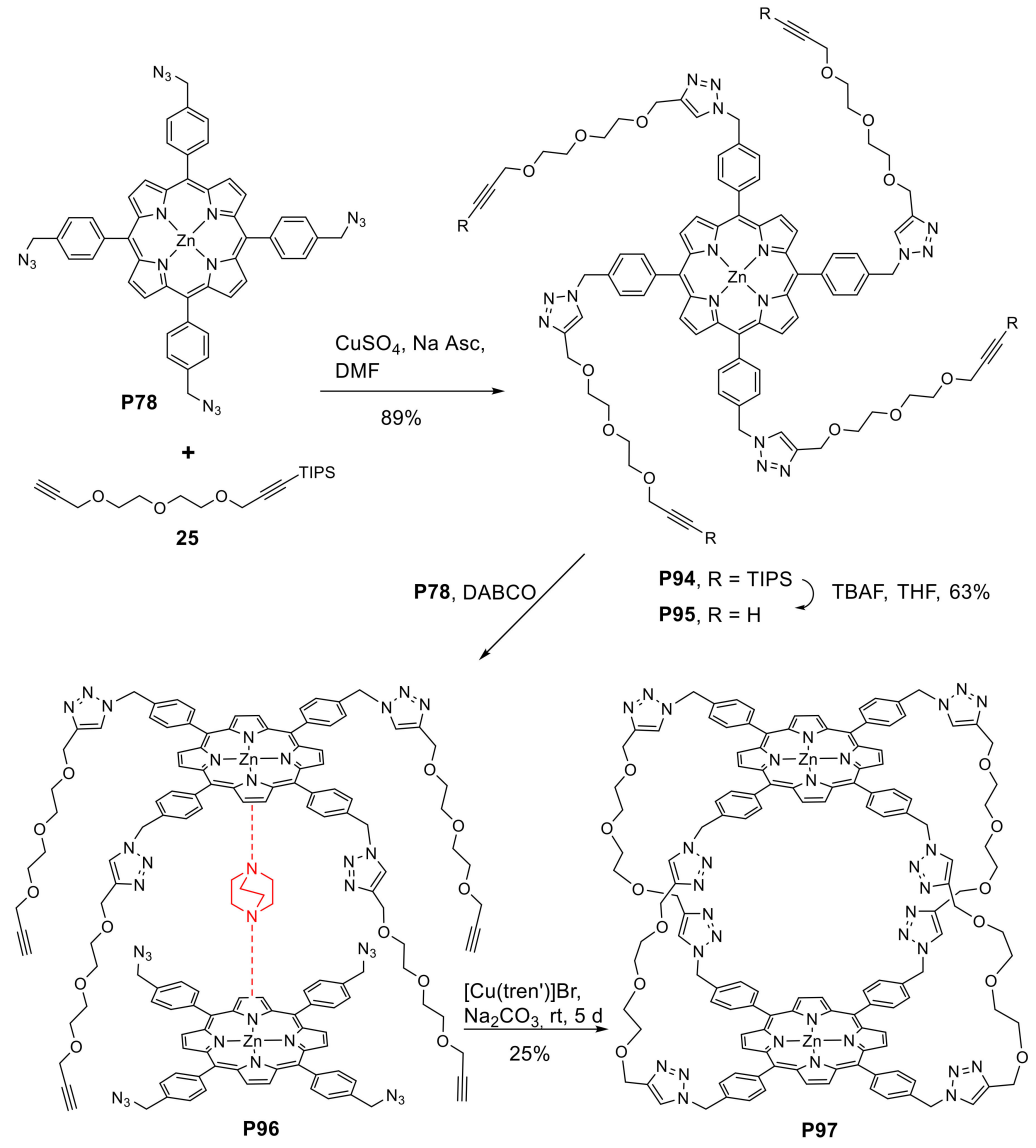
Synthesis of a porphyrin cage.

The covalent cage **P97** consisting of two porphyrins connected by four flexible spacers each incorporating two 1,2,3-triazolyl ligands has been synthesized starting from the *meso*-tetrakis(4-azidomethylphenyl)porphyrin **P78** and the TIPS-monoprotected alkyne **25** (Scheme 33) [72]. The first CuAAC reaction gave rise to compound **P94** at a 89% yield. Deprotection of the TIPS-protected alkyne using TBAF led to porphyrin **P95**, the second precursor of the cage. The final step involved the formation of the DABCO-templated hetero dimer **P96** (in a dynamic equilibrium mixture with two homo dimers) and the construction of the last four triazolyl groups. The last cyclisation reaction was performed in CH_2_Cl_2_ at room temperature using [Cu(tren’)]Br as catalyst and Na_2_CO_3_ as a base. The reaction was completed after five days and the cage **P97** was obtained at a 25% yield. Due to the flexibility of the ether spacers between the triazole moieties this cage adopts a compact conformation in solution stabilized by π-π interactions. However, the coordination of the triazole moieties with four Ag(I) ions locks the porphyrin macrocycles in a face-to-face disposition, leading to the formation of a rigid cage.

**Scheme 34:**
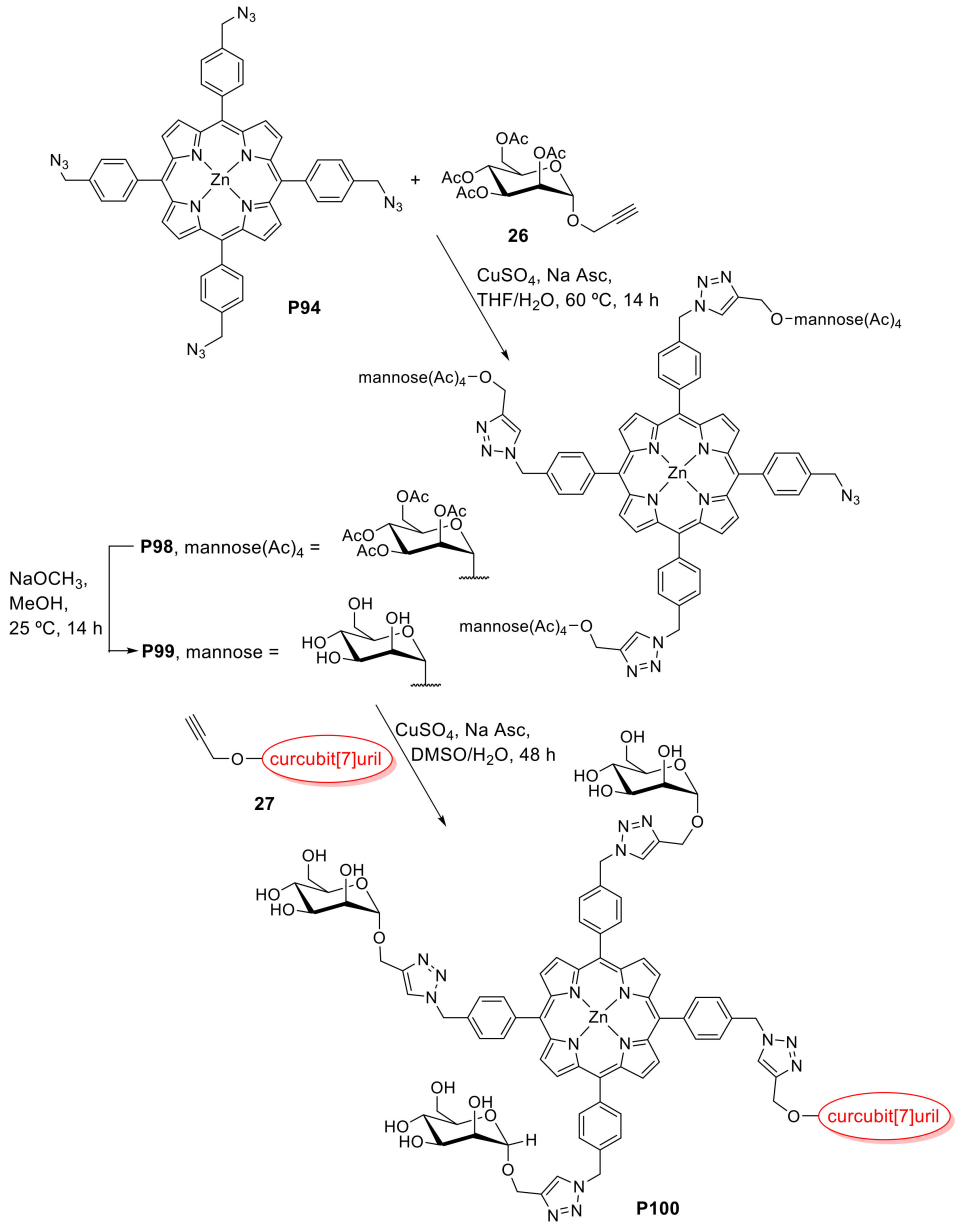
Synthesis of a glycosylated porphyrin–cucurbituril conjugate.

Tuncel and co-workers reported the synthesis of a photoactive glycosylated porphyrin covalently attached to a monofunctionalized cucurbit[7]uril unit [73]. The synthesis of the target compound involved an initial CuAAC reaction involving the tetraazidoporphyrin **P78** with the propargylated tetraacetylmannose **26** (Scheme 34). The resulting monoazidoporphyrin **P98** was then deacetylated using sodium methoxide in methanol and, finally, a new CuAAC reaction between azide **P99** and the monopropargyloxycucurbit[7]uril **27** afforded the multifunctional porphyrin derivative **P100**. The host–guest chemistry of conjugate **P100** was investigated by ^1^H NMR experiments using a bisimidazolium guest and the formation of an inclusion complex with the cucurbit[7]uril was observed. It was also found that the ^1^O_2_ generation efficiency of the synthesized compound was significantly higher than that of the unfunctionalized porphyrin, indicating that this supramolecular assembly can serve as an efficient photosensitizer for biological applications.

In 2019, Tuncel and co-workers reported the use of the glycosylated porphyrin-cucurbituril conjugate **P100** as a photosensitizer in photodynamic antibacterial and cancer therapy and as a drug carrier [74]. The results of the study showed that this photosensitizer efficiently inactivated both Gram-negative (*Escherichia coli*) and Gram-positive bacteria (*Bacillus subtilis*) when exposed to white light for 1 min (20 mW/cm^2^). The in vitro citotoxicity and photocytotoxicity of conjugate **P100** on the MCF7 breast cancer cell line revealed that it did not show any cytotoxic effect in the absence of light but caused a significant decrease in the viability of the cancer cells when exposed to white light for 5 min (20 mW/cm^2^). It was also demonstrated that conjugate **P100** served as a doxorubicin carrier for chemo-photodynamic dual cancer therapy.

**Scheme 35:**
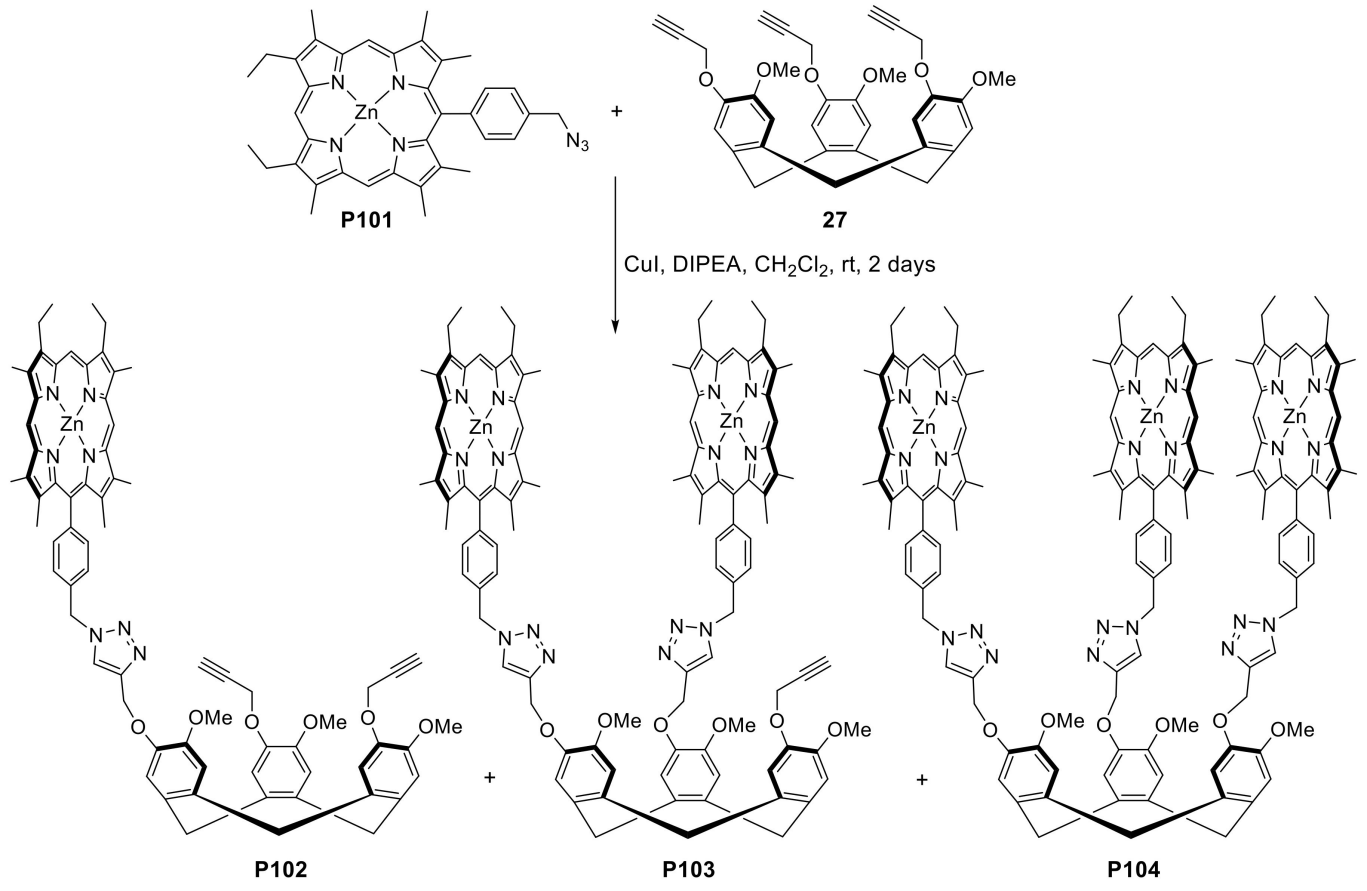
Synthesis of cyclotriveratrylene derivatives covalently bonded to 1, 2 or 3 Zn(II) porphyrin units.

**Scheme 36:**
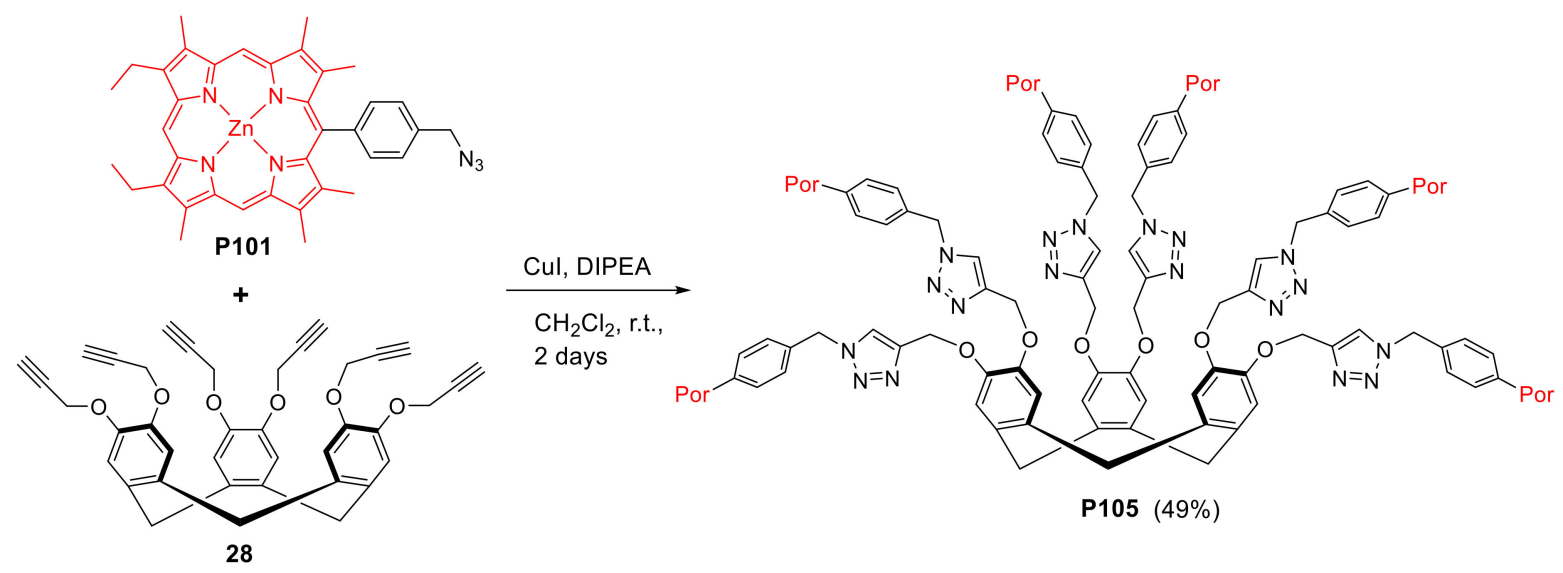
Synthesis of a cyclotriveratrylene derivative covalently bonded to six Zn(II) porphyrin units.

Gros, Harvey and co-workers reported the synthesis of cyclotriveratrylene derivatives covalently bonded to 1, 2, 3, or 6 Zn(II) porphyrin units via click chemistry (Scheme 35 and Scheme 36) [75]. The new compounds were used as hosts for C_60_ but the binding constants were considered to be not good (*K*_a_ between 400 and 4000 M^−1^). The formation of host-guest assemblies with C_60_ was studied using absorption and fluorescence spectroscopy, including fluorescence quenching by C_60_. Surprisingly, the largest *K*_a_ value was obtained for the monoporphyrinyl derivative **P102**, which exhibits the least sterically demanding structure. Computer modelling studies revealed that the flexibility of the anchoring chains between the cyclotriveratrylene bowl and the porphyrin units prevented favorable conformation to capture C_60_. Instead, pincer conformations were found to be responsible for the host-guest associations.

#### 2.2.3. 2-Azido- or 2-Azidomethylporphyrins

**Scheme 37:**
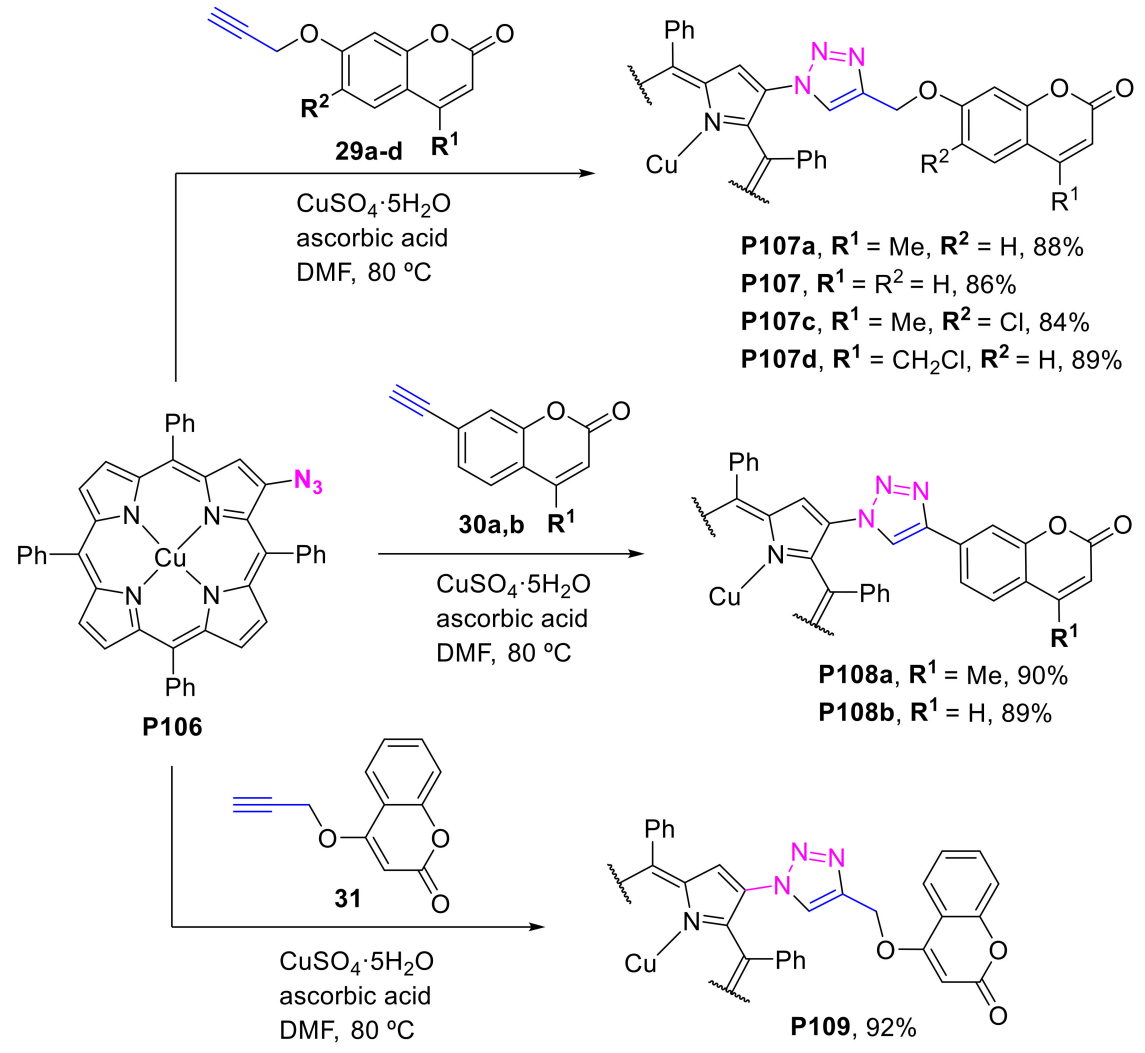
Synthesis of β-triazole bridged coumarin–Cu(II)porphyrin conjugates.

Singh and Nath reported the synthesis of β-triazole bridged coumarin-porphyrin conjugates by the CuAAC reaction between 2-azido-5,10,15,20-tetraphenylporphyrinatoCu(II) (**P106**) with various alkyne-substituted coumarins (**29**–**31**) (Scheme 37) [76]. The corresponding free-bases were obtained in a good yield (71%–80%) by demetalation of the Cu(II) derivatives. Metalation of the free-bases with Zn(II) acetate afforded the corresponding Zn(II) complexes. The photophysical characterization of these conjugates revealed, for some of them, a considerable electronic communication between both units. Additionally, in some of these dyads, a significant intramolecular energy transfer between both chromophores was observed.

**Scheme 38:**
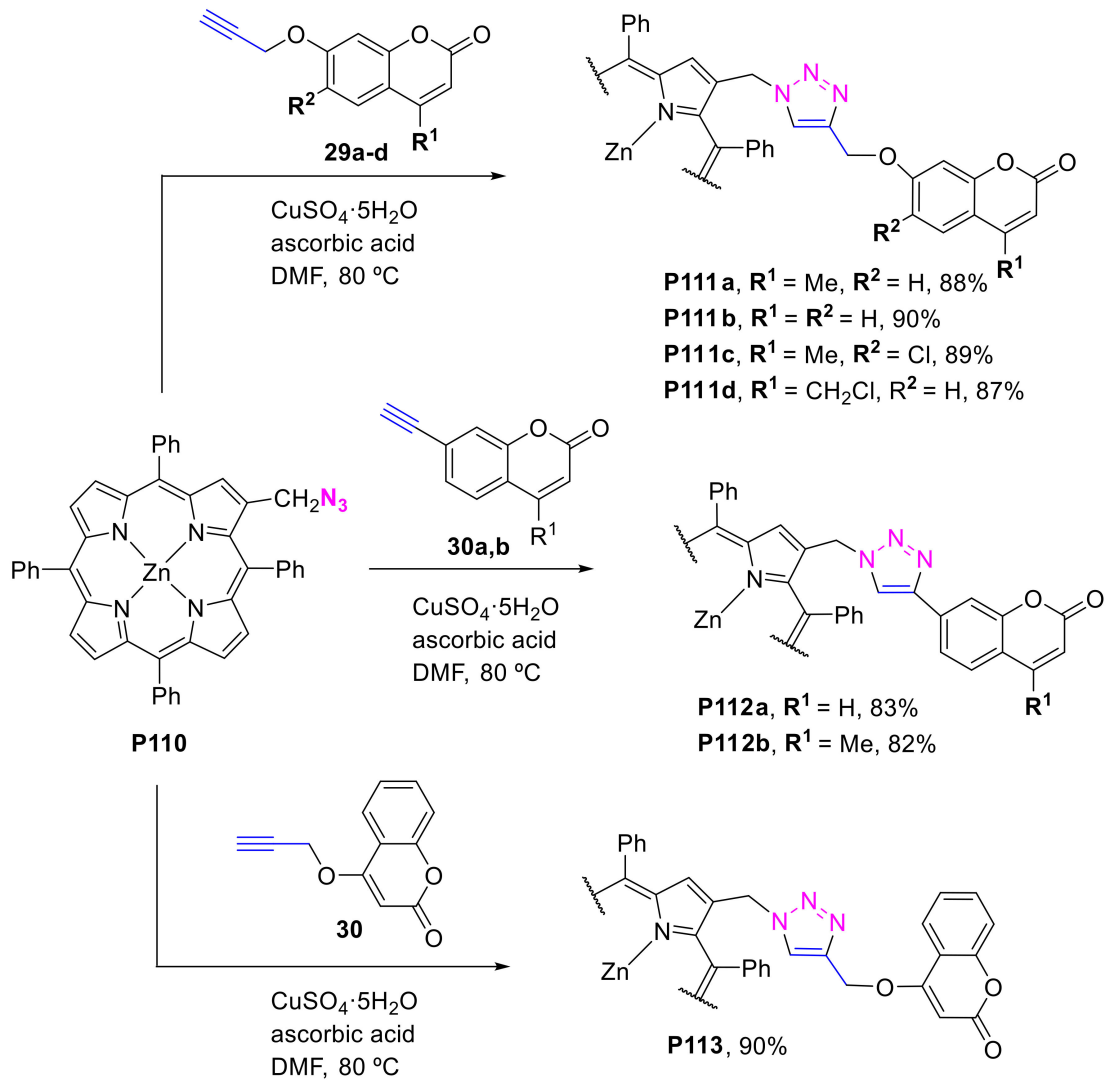
Synthesis of Zn(II) β-triazolylmethyl-bridged coumarin–porphyrin dyads.

Using a similar strategy, the same authors also synthesized the Zn(II) β-triazolylmethyl-bridged coumarin–porphyrin dyads **P111**–**P113** (Scheme 38) [77]. Their synthesis involved the 1,3-dipolar cycloaddition between the 2-azidomethyl-5,10,15,20-tetraphenylporphyrinatozinc(II) (**P110**) and the alkyne-substituted coumarins **29**–**31**. The reported yields for the dyads are in the range of 84%–92%. Demetalation of the Zn(II) complexes afforded the corresponding dyads with free-base porphyrins. The photophysical characterization of the new compounds showed the occurrence of an intramolecular energy transfer between the units of some conjugates.

**Scheme 39:**
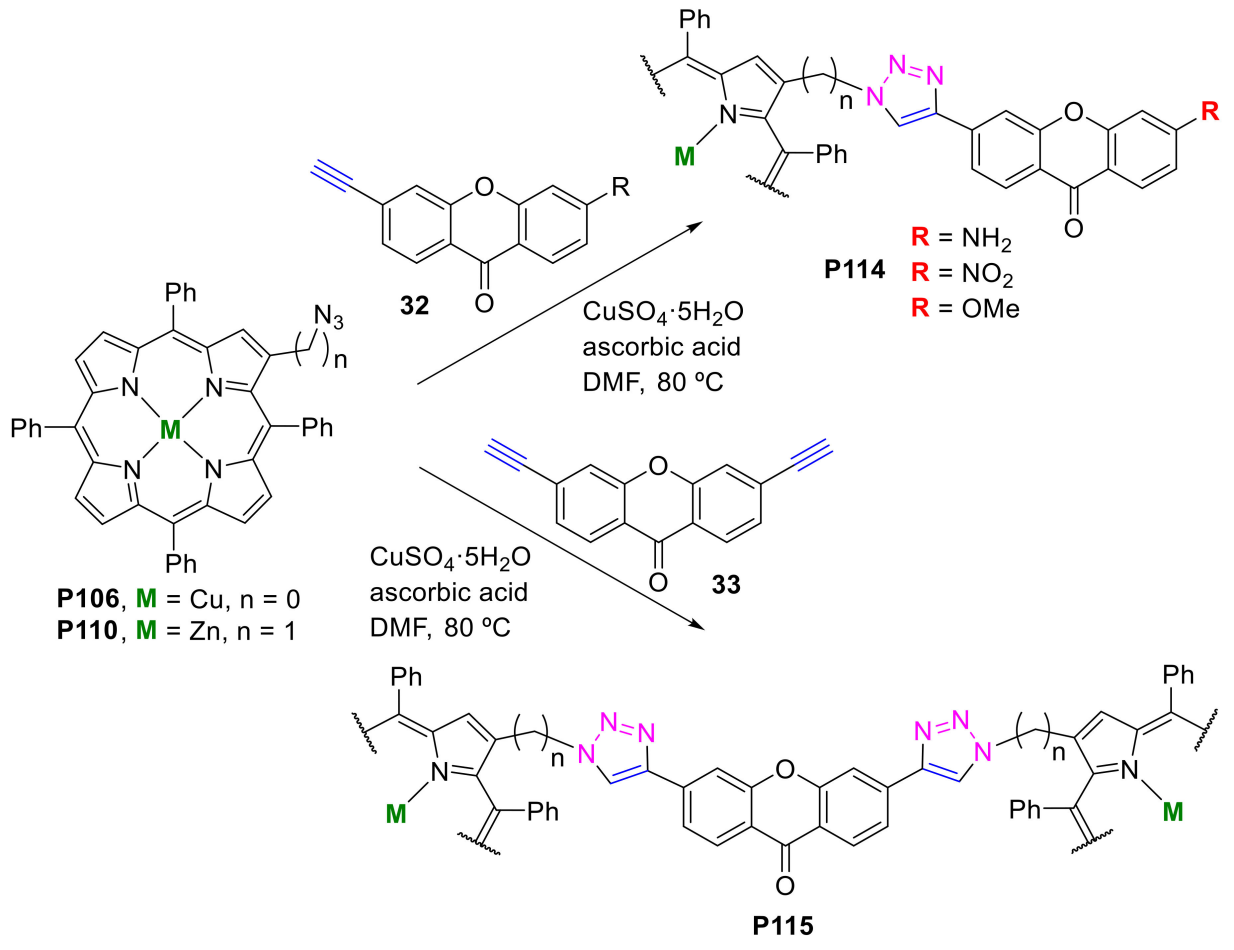
Synthesis of porphyrin-xanthone conjugates.

The same authors also reported the use of porphyrins **P106** and **P110** for the synthesis of β-triazolyl- and β-triazolylmethyl-bridged porphyrin-xanthone conjugates [78]. For both porphyrin derivatives, 1:1 (**P114**) and 2:1 (**P115**) porphyrin-xanthone conjugates were prepared (Scheme 39). The photophysical evaluation of these conjugates revealed a bathochromic shift in their electronic absorption and fluorescence spectra when compared to the spectra of the *meso*-tetraarylporphyrins.

#### 2.2.4. Other Azido-Substituted Porphyrins

**Scheme 40:**
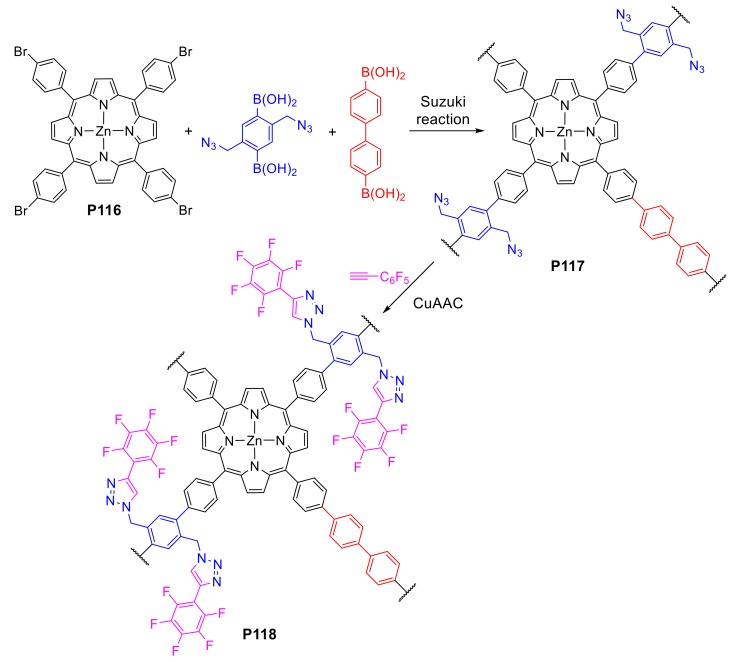
Synthesis of fluoride functionalized conjugated microporous polymers.

Xu and co-workers reported the synthesis of a series of fluoride functionalized conjugated microporous polymers (CMPs) based on Zn(II) porphyrin building blocks [79]. The synthetic route involved the Suzuki-coupling reaction of *meso*-tetrakis(*p*-bromophenyl)porphyrinatozinc(II) (**P116**) with a mixture of 4,4’-biphenyldiboronic acid and (2,5-bis(azidomethyl)-1,4-phenylene)diboronic acid at varying molar ratios (Scheme 40). The azide groups incorporated within the pores of the resulting CMPs **P117** were then subjected to a CuAAC reaction with pentafluorophenylethyne. The resulting porous frameworks **P118** exhibited enhanced CO_2_ sorption properties.

**Figure 10:**
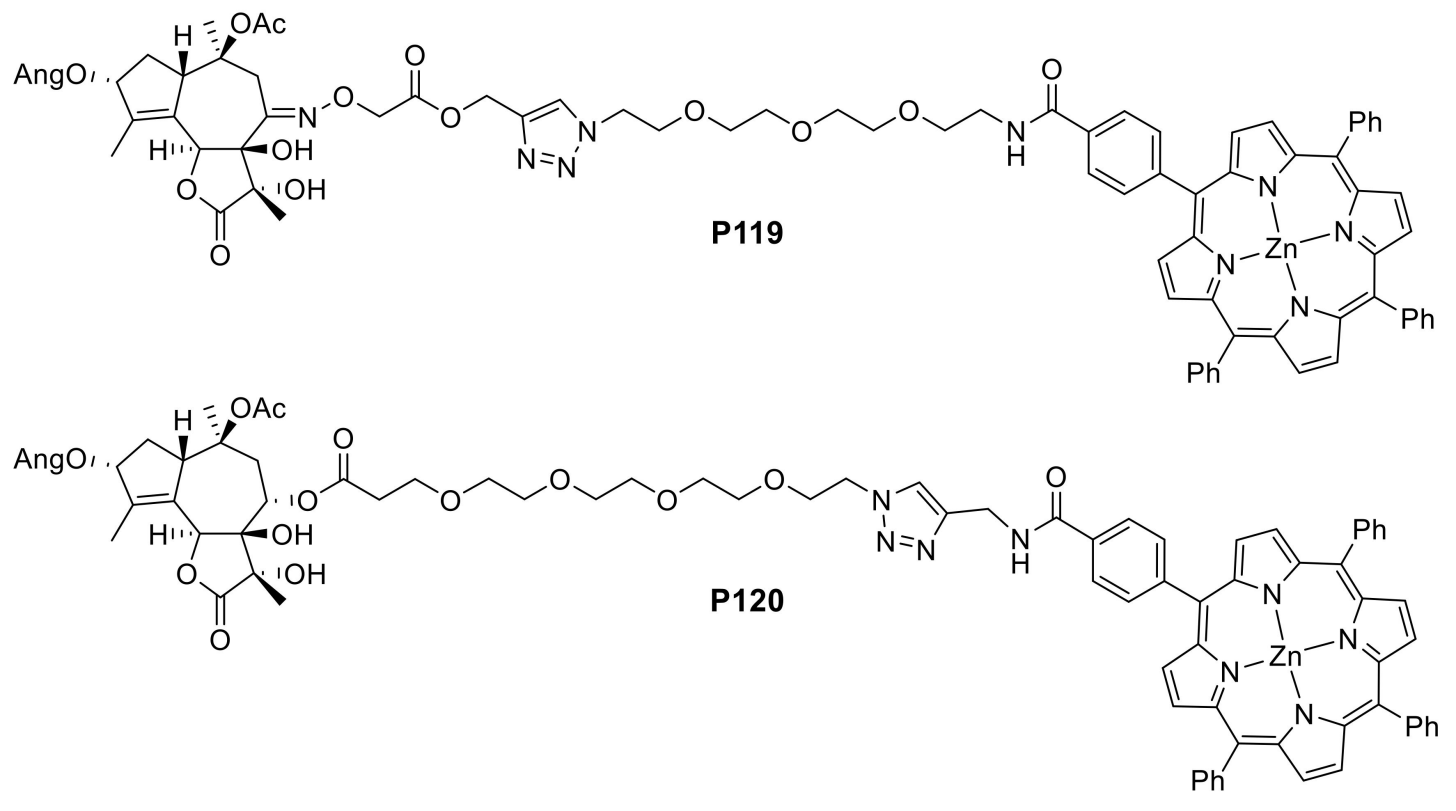
Structures of trilobolide-porphyrin conjugates.

Drašar and co-workers reported the synthesis of the two trilobolide-porphyrin conjugates **P119** and **P120** (Figure 10) [80]. Trilobolide is a natural sesquiterpene lactone isolated from horse caraway (Laser trilobum, L. Borkh) that exerts significant pharmacological properties. The trilobolide unit was linked to porphyrin derivatives via CuAAC reactions and the in vitro cytotoxicity of the resulting conjugates was tested against rat peritoneal cells and four human tumor cell lines: LNCaP (prostate carcinoma), U-2 OS (osteosarcoma), MCF-7 (breast carcinoma), and MiaPaCa-2 (pancreatic carcinoma). The intracellular localization of the conjugates was investigated using live-cell fluorescence microscopy. Both conjugates were localized in mitochondria and lysosomes of HeLa and LNCaP cells at 5.0 µM concentration after 2 h.

**Scheme 41:**
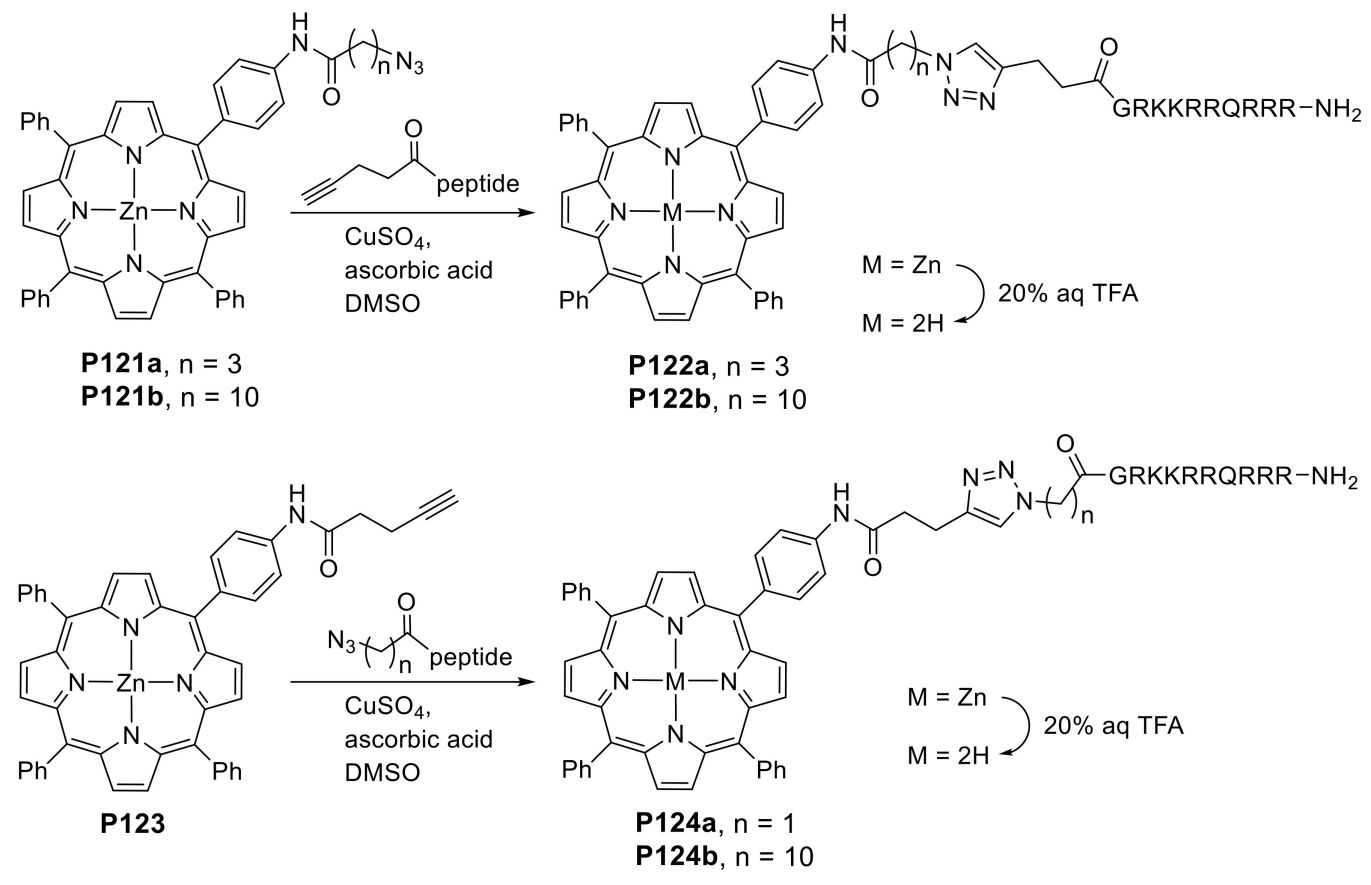
Synthesis of cell-penetrating peptide-porphyrin conjugates.

Eggleston and co-workers reported the synthesis of several cell-penetrating peptide-porphyrin conjugates using a diversity of bioconjugation reactions, including the CuAAC approach [81]. That work aimed to convert classical hydrophobic PDT agents into amphiphilic conjugates suitable for targeted photochemical internalization and PDT. Examples of the synthesized conjugates are illustrated in Scheme 41.

**Scheme 42:**
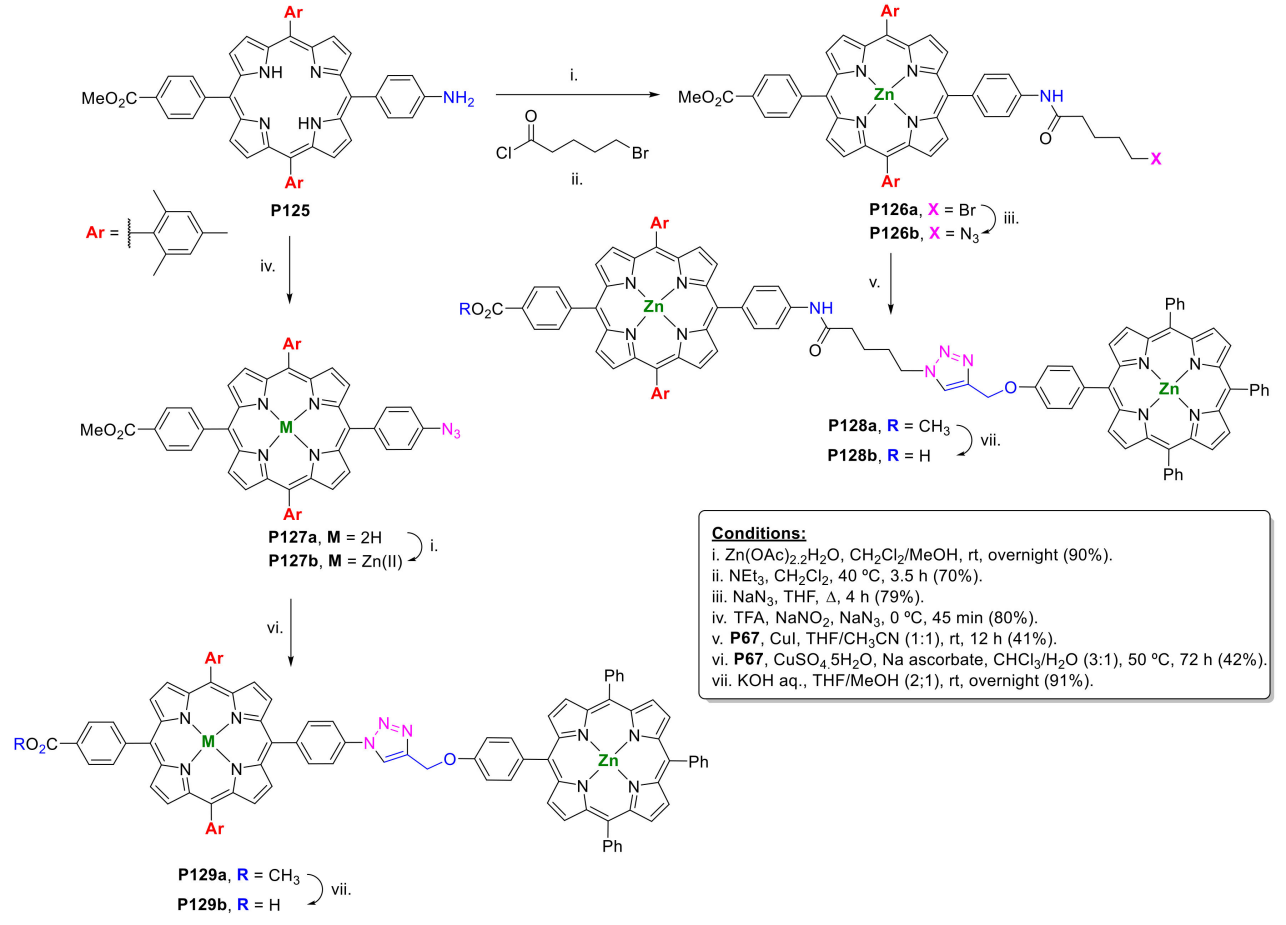
Synthetic approach to porphyrin-based dyads **P128** and **P129** bearing triazolyl bridge.

Coutsolelos and co-workers explored the potential application as sensitizers for DSSCs of two porphyrin-based dyads linked by a variable-length triazole bridge [82]. The A_2_BC porphyrin derivative **P125** bearing an ester and an amino group at *para* positions of two opposite *meso*-phenyl rings was functionalized with an azide group by following two different synthetic pathways. One of them involved the insertion of an alkyl chain as a spacer between the *meso*-phenyl and the azido group by reaction of the amino-porphyrin **P125** with 5-bromopentanoyl chloride, which yielded the corresponding bromoalkyl porphyrin amide derivative **P126a**, this was then converted into the azide derivative **P126b** by treatment with NaN_3_. A second strategy involved the treatment of porphyrin **P125** with NaNO_2_ and NaN_3_ in acidic medium allowing the preparation of derivative **P127** with the azide group directly linked at the *meso*-phenyl group (Scheme 42).

Porphyrin-azide adduct **P126b** was reacted with porphyrin-alkyne **P67** via a click CuAAC reaction in the presence of CuSO_4_/ascorbate catalytic system affording the triazolyl bridged porphyrin dyad **P128a** at a 42% yield. Dyad **P129a** was synthesized (41% yield) by following an analogous synthetic approach; however, since the azide is directly linked to a phenyl ring, the replacement of the CuSO_4_/ascorbate catalytic system by catalytic amounts of CuI in the presence of DIPEA was required [82,83]. Basic hydrolysis of the methyl ester groups of precursors **P128a** and **P109a** gave rise to target porphyrinic dyads **P128b** and **P129b** at a 91% yield. Compound **P129b**, with a smaller triazolyl bridge, displayed a higher photovoltaic performance than the analogous derivative **P128b** (5.1% vs. 3.8%) when attached to titanium oxide via the carboxyl group [82].

**Scheme 43:**
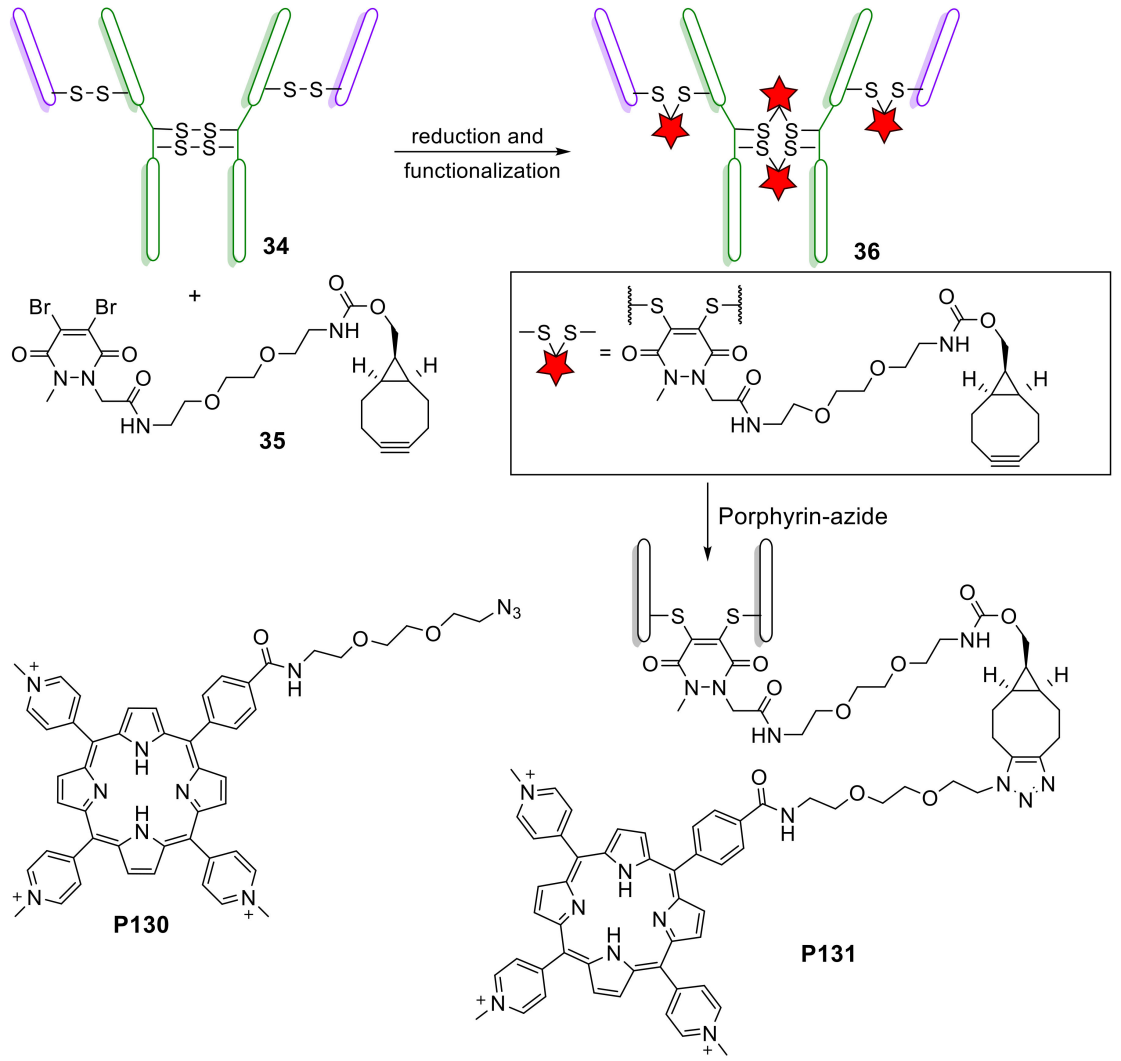
Synthesis of a trastuzumab-porphyrin conjugate.

Boyle, Chudasama and co-workers developed a method for the site-selective modification of a full antibody to provide a defined multi-porphyrin antibody conjugate, which showed to be active in vitro [84]. The experimental procedure involved the synthesis of the dibromopyridazinedione-strained alkyne **35** that was used for the functional re-bridging of the disulfides of trastuzumab (**34**, a clinically approved antibody for the treatment of breast cancer) (Scheme 43). The insertion of the dibromopyridazinedione **35** into the disulfide bonds was carried out by adding tris(2-carboxyethyl)phosphine (a reducing agent) to a solution of trastuzumab in borate buffered saline solution (pH 8.0), containing an excess of **35**, and the mixture was incubated at 4 °C for 16 h. The resulting conjugate **36** was then coupled with the water-soluble porphyrin azide **P130** to afford the trastuzumab–porphyrin conjugate **P131**. This conjugate **P131** was formed in near quantitative yield in the reaction of conjugate **36** with five equivalents of the porphyrin azide for 4 h at 37 °C. Conjugate **P131** exhibited remarkable abilities to eradicate HER2+ cells (*ca.* 90% kill) while, at the same concentration, HER2–cells were unaffected. In contrast, trastuzumab alone showed, on irradiation, no cytotoxicity and minimal cytotoxicity was observed for the unconjugated porphyrin **P130** with both cell lines.

**Scheme 44:**
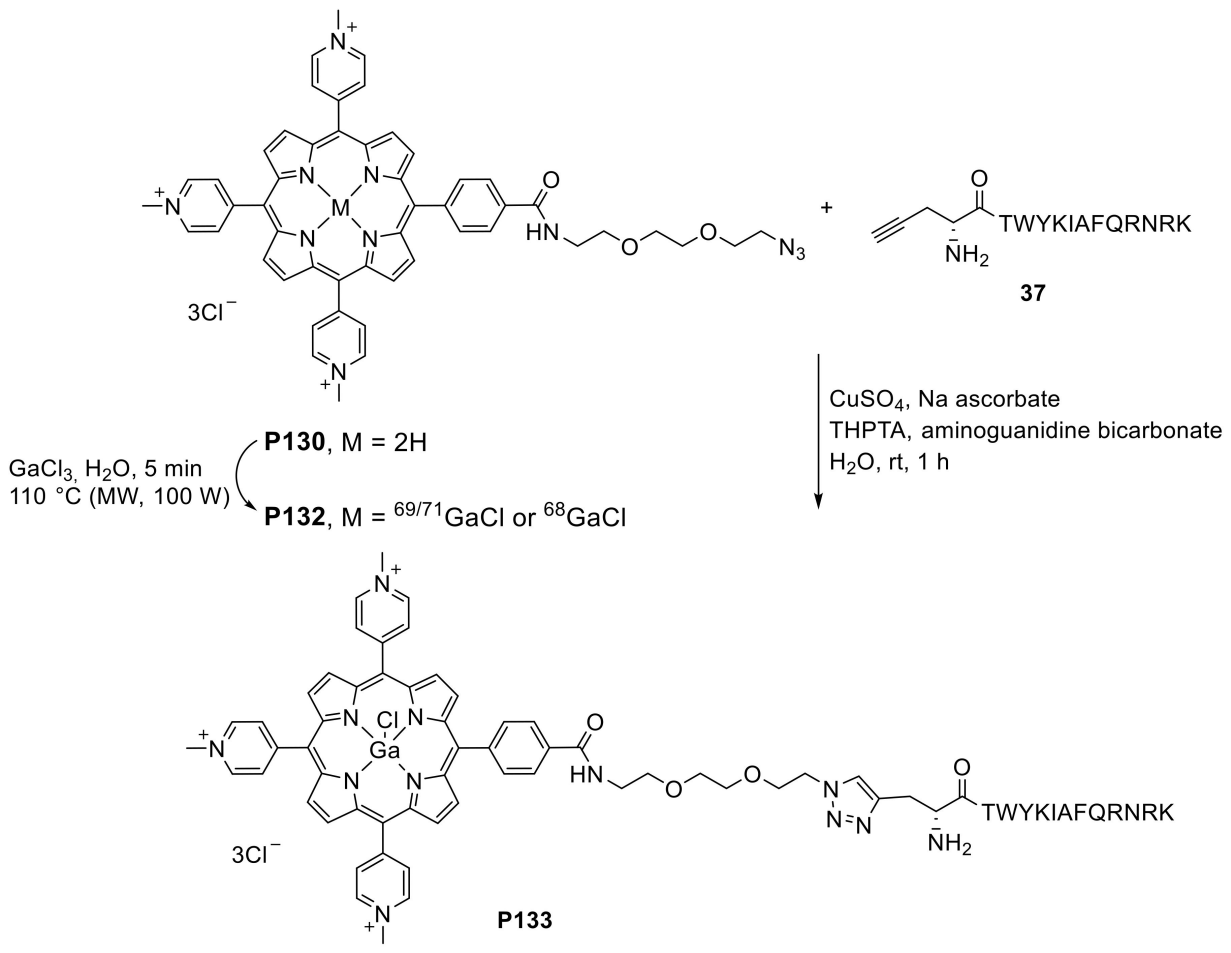
Synthesis of a radiolabeled peptide-porphyrin derivative.

Boyle and co-workers also used the tricationic porphyrin **P130** to produce a molecular theranostic agent suitable for use as a PET radiotracer and as a photosensitizer for PDT [85]. These authors developed a procedure to prepare ^69/71^Ga and ^68^Ga radiolabeled azide-functionalized porphyrins **P132,** and the metalated porphyrins were then bioconjugated to the alkyne-functionalized dodecapeptide TWYKIAFQRNRK **37** (Scheme 44). This peptide exhibits a good affinity for the α6β1-integrin, which is involved in cellular migration and is also upregulated in multiple cancers, including breast carcinomas and glioblastomas. The radiolabeled peptide-porphyrin conjugate **P133** exhibited excellent ability to eradicate the high integrin expressing HeLa cell line (*ca.* 80% kill), while at the same concentration showed considerably lower cell killing in a cell line displaying minimal integrin expression (U87).

**Scheme 45:**
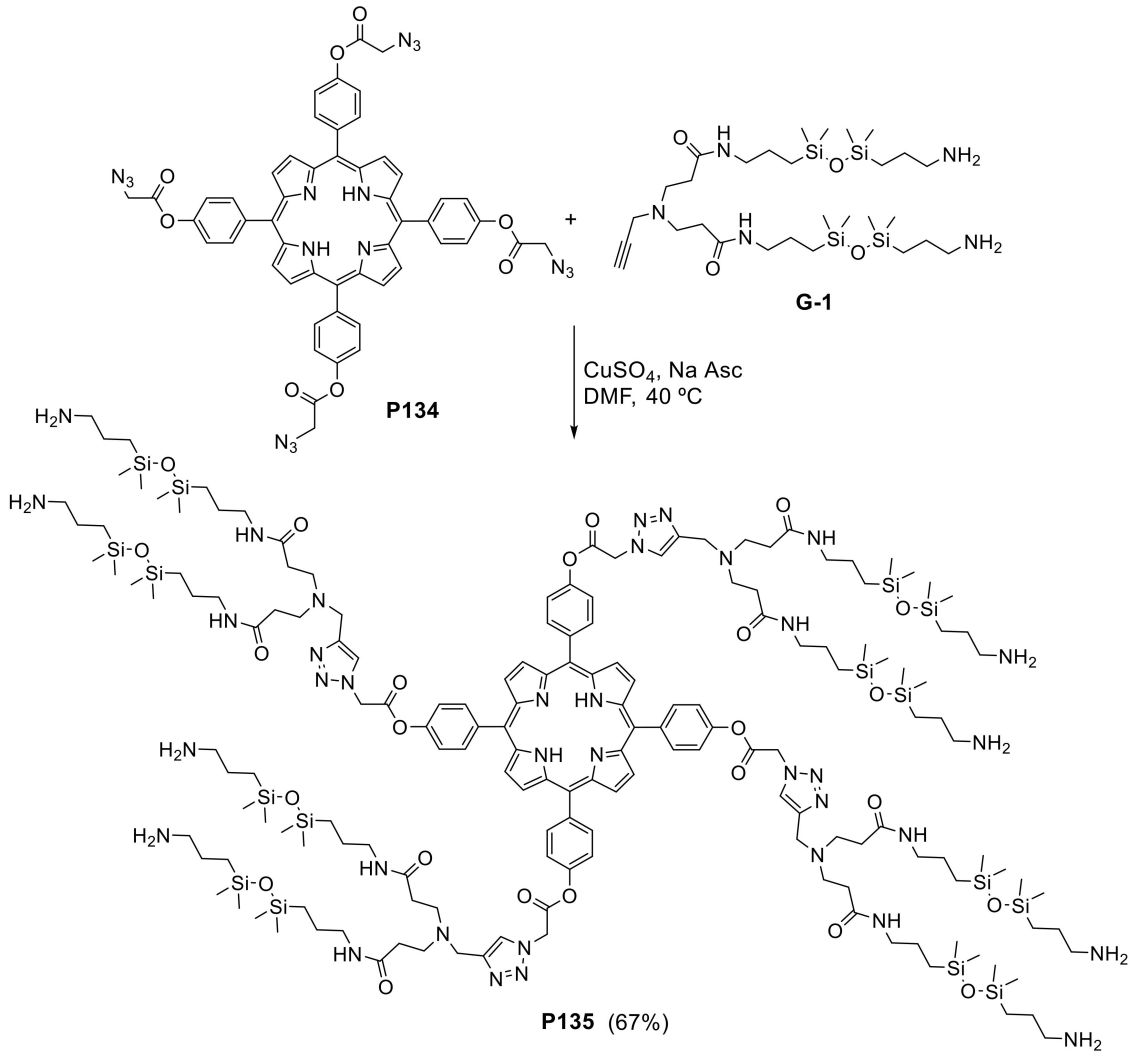
Synthesis of porphyrin-cored dendrimers containing siloxane-poly(amidoamine) dendron-like arms (shown G-1 only).

The CuAAC reaction was used to synthesize different generations (from 1 to 3) of porphyrin-cored dendrimers consisting of siloxane-poly(amidoamine) dendron-like arms (Si-PAMAM) as illustrated in Scheme 45 for G-1 [86]. The encapsulation of the porphyrin unit inside the dendritic Si-PAMAM shell (**P135**) allowed to increase the solubility of porphyrins and to prevent their aggregation in aqueous solutions. The porphyrin-cored dendrimers showed strong fluorescence emission and the intensity increased significantly as the generation is growing. The dendrimers also showed to be pH-responsive in fluorescence emission, indicating that they may be able to act as pH-responsive probes suitable for biomedical imaging, diagnosis and treatment.

More recently, the same research group, and using the same approach, reported the synthesis of glycosylated porphyrin-cored Si-PAMAM dendrimers [87]. The new glycoporphyrin dendrimers exhibited high fluorescence quantum yields and singlet oxygen production efficiency, but also showed specific recognition of lectin and temperature-responsive property (20–80 °C).

**Figure 11:**
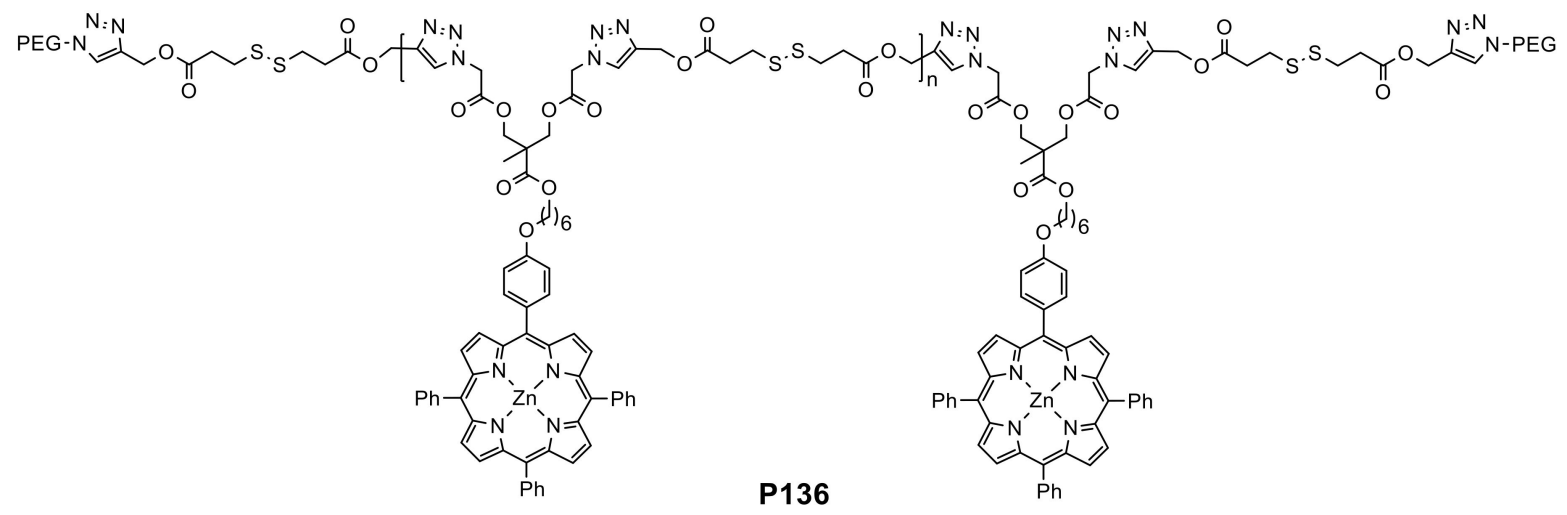
Structure of the PEG-terminated ZnTPPC_6_-based polymer.

In 2019, Zhang and co-workers reported the synthesis of an amphiphilic polymer **P136** (Figure 11) that can be self-assembled into micelles with excellent stability, ultra-fast sensitivity of redox-triggered porphyrin release, and significant photodynamic anticancer performance [88]. The in vitro biological results revealed that these micelles can effectively enhance the cellular uptake and cellular internalization of porphyrin, have an extremely low dark toxicity, and are efficient towards A549 cells upon light irradiation with visible LED lamp.

**Scheme 46:**
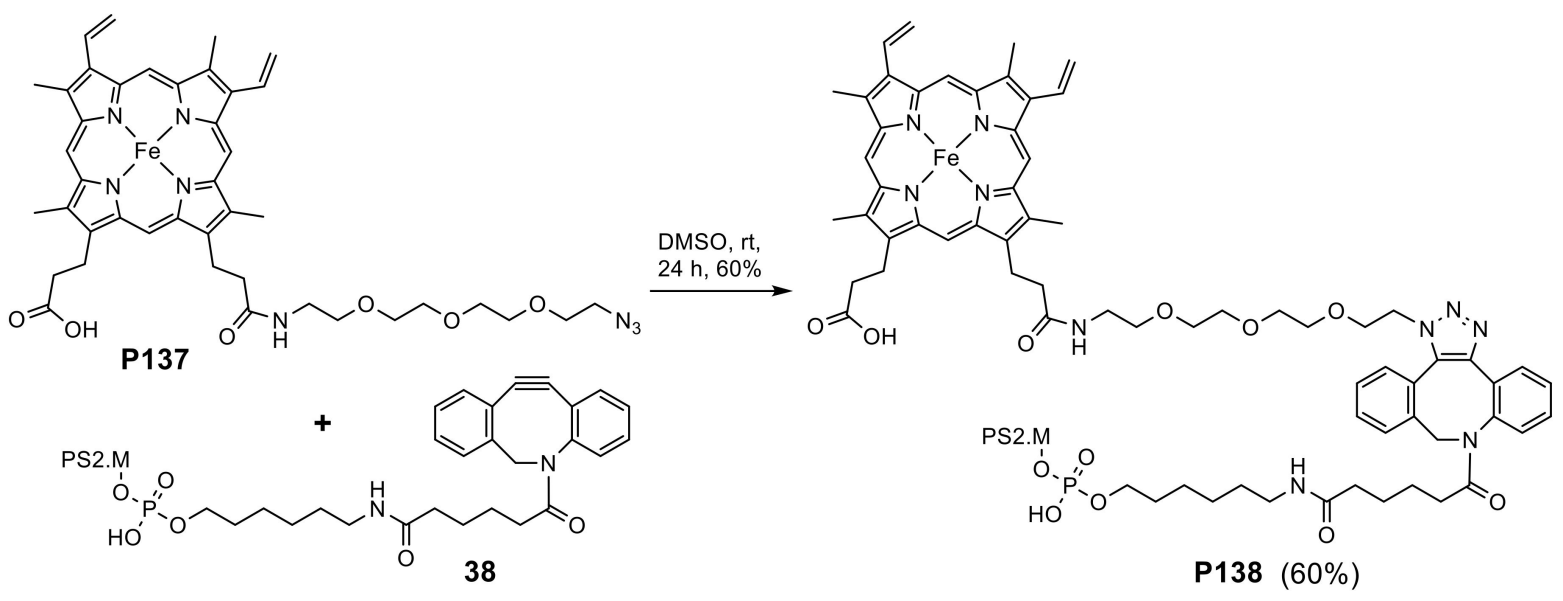
Synthesis of a PS2.M-hemin conjugate.

A successful conjugation of hemin to the G-quadruplex DNA was reported in 2017 [89]. The G-quadruplex-hemin conjugate **P138** was prepared from the reaction of the azide-modified hemin **P137** with a modified PS2.M oligonucleotide (5’GTG GGT AGG GCG GGT TGG3’) **38** with dibenzoazacyclooctynyl groups using the Cu-free strain-promoted alkyne-azide cycloaddition approach (Scheme 46). The PS2.M-hemin conjugate exhibits peroxidase activity and catalyzes the oxidation of fluorogenic substrate 4-(1-methylhydrazineyl)-7-nitrobenzo[c][1,2,5]oxadiazole but with slightly lower efficiency when compared to PS2.M/hemin complex.

**Figure 12:**
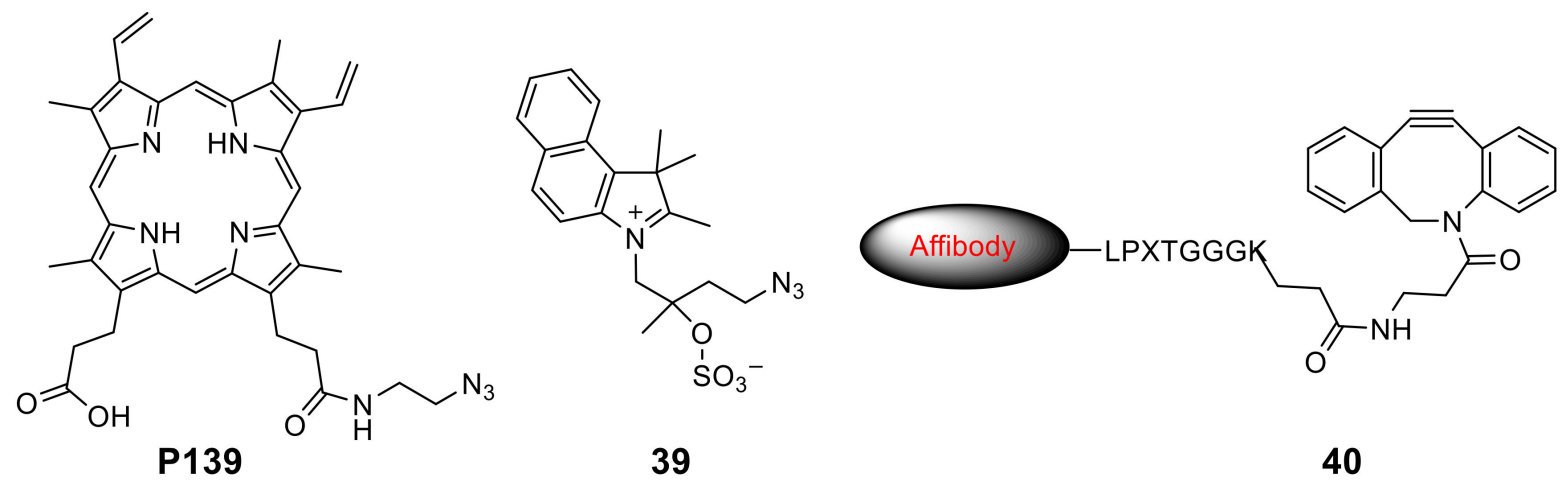
Structures of the azide-dyes and alkyne-affibody used for the preparation of affibody-conjugated nanoclusters.

In 2018, Tsourkas and co-workers reported a new approach for the site-specific labeling of dye-stabilized nanoemulsions with affibodies for cellular targeting [90]. The method involved the preparation of dye-stabilized nanoemulsions bearing azide-handles on the surface. The azide groups were then conjugated with site-specific affibodies labeled at the *C*-terminus with a constrained alkyne, namely dibenzoazacyclooctyne. The affibody-conjugated nanoclusters were prepared by the Cu-free strain-promoted alkyne-azide cycloaddition approach by reacting the azide-functionalized nanoclusters (based on **P139** and **39**) with alkyne-functionalized affibody **40** in PBS for 12 h at room temperature with shaking (Figure 12).

**Scheme 47:**
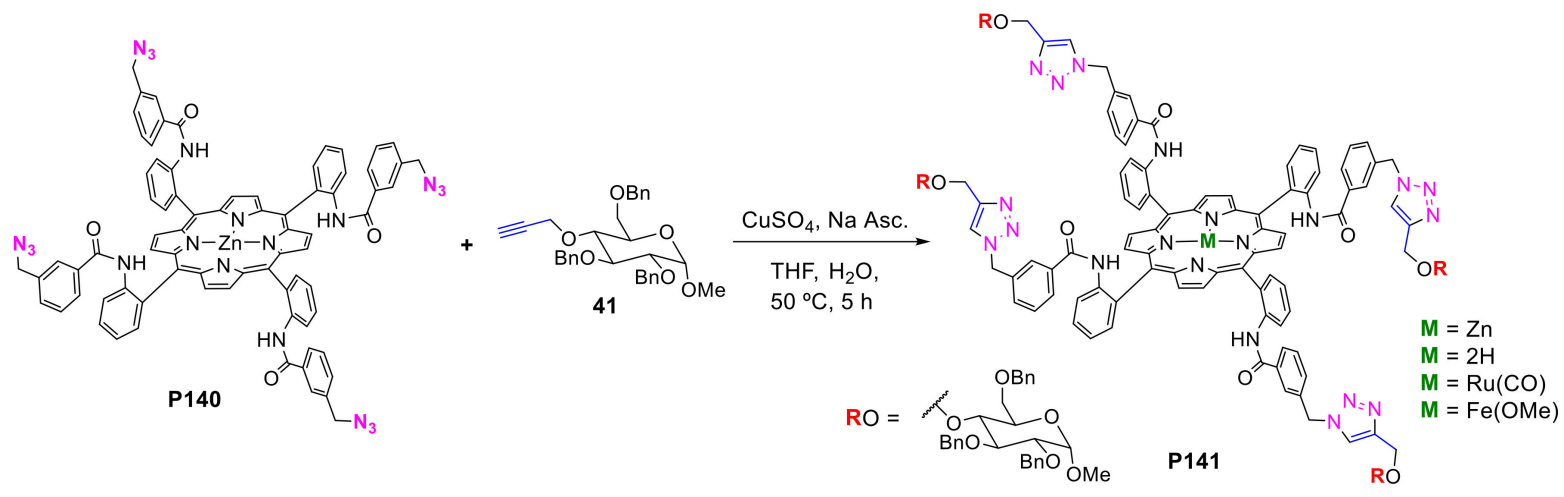
Synthesis of glycoporphyrins used as catalysts in cyclopropanation and aziridination reactions.

Lay, Gallo and co-workers report the synthesis of iron and ruthenium glycoporphyrins using the CuAAC approach [91]. The new derivatives were used as catalysts in cyclopropanation and aziridination reactions using diazo compounds and aryl azides as carbene and nitrene precursors, respectively (see Section 3.). As illustrated in Scheme 47, glycoporphyrins **P141** were obtained from the reaction of tetraazidoporphyrin **P140** and the methyl 4-*O*-propargyl-α-d-glucopyranoside **41**.

### 2.3. Other Reactions Involving Azides

**Scheme 48:**
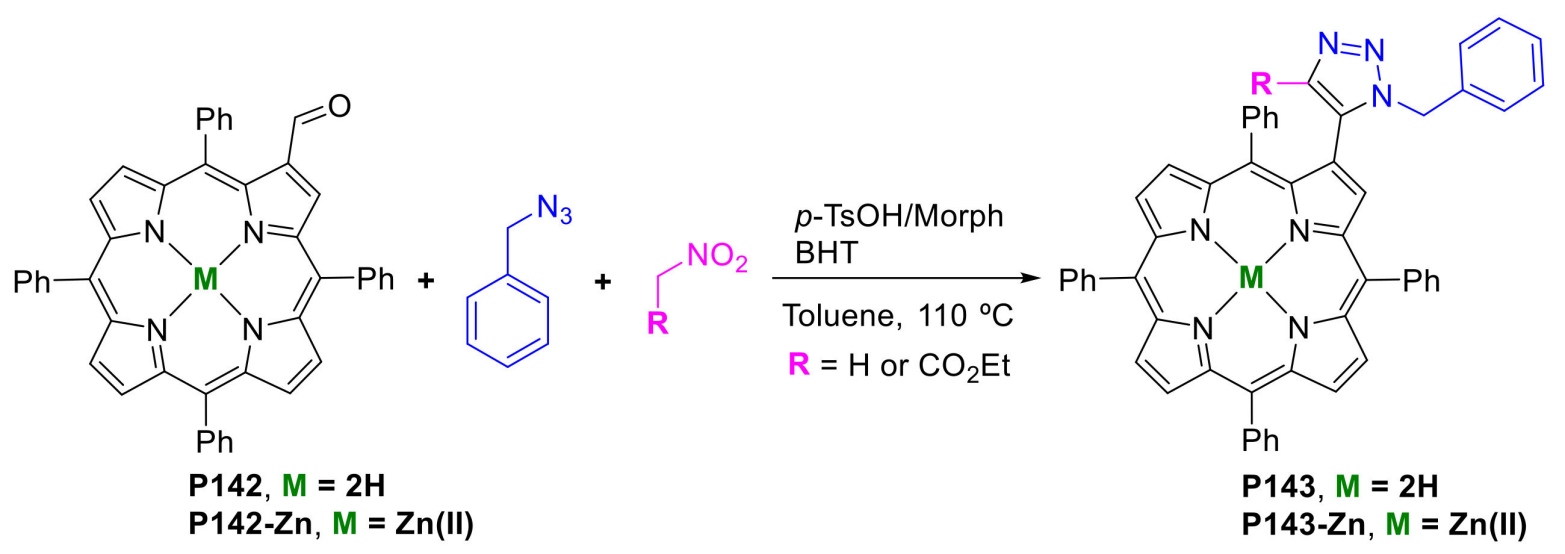
One-pot synthesis of 2-(1,4,5-trisubstituted-1,2,3-triazolyl)porphyrin derivative **P143**.

The reactivity of porphyrin derivatives with azide compounds was also explored in other synthetic routes not involving alkyne derivatives. For instance, Vroemans and co-workers described a synthetic method using 2-formyl-*meso*-tetraphenylporphyrin **P142** as starting material in a catalyzed multicomponent synthetic approach [92]. The reaction of **P142** with benzyl azide and nitroalkanes was performed under a nitrogen atmosphere in the presence of a mixture of *p*-toluenesulfonic acid/morpholine salt in catalytic amounts and butylhydroxytoluene as an anti-oxidant. When the reaction was carried out with the non-activated nitromethane the 2-(1,5-disubstituted-1,2,3-triazolyl)porphyrin derivative, **P143** (R = H) was obtained at a 22% yield. However, the reaction with ethyl nitroacetate afforded 2-(1,4,5-trisubstituted-1,2,3-triazolyl)porphyrin **P143** (R = CO_2_Et) at a 45% yield (Scheme 48). Similar yields were obtained when the reaction was performed with the Zn(II) complex of 2-formylporphyrin **P142-Zn**. All these reactions were regioselective.

**Scheme 49:**
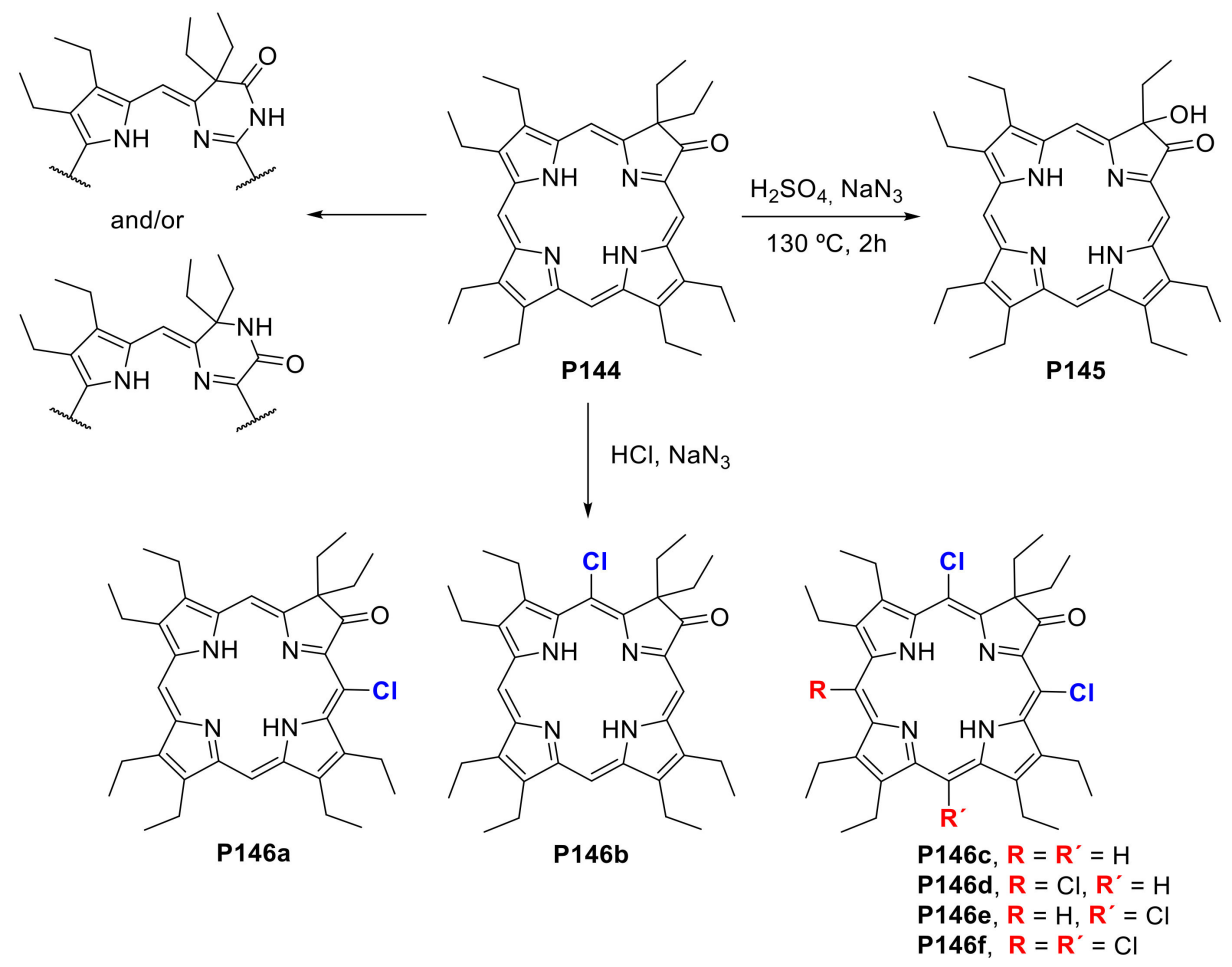
Products resulting from the sodium azide acidic treatment of oxochlorin **P144**.

The Schmidt reaction conditions, involving the treatment of ketones with sodium azide in an acidic medium, are usually used to prepare lactams from cyclic ketones by ring expansion and is an alternative to the Beckmann rearrangement. However, when Brückner and co-workers treated the octaethylporphyrin derivative **P144** under the Schmidt reaction conditions a α-hydroxyoxochlorin derivative was isolated (**P145**), or *meso*-chlorinated products (**P146**), instead of the expected lactams [93]. The reaction with the oxochlorin **P144** required the use of a large excess of NaN_3_ and conc. H_2_SO_4_ at 130 °C. After 2 h of reaction, a green product corresponding to **P145** was isolated as a racemic mixture with a 23% yield (Scheme 49). The authors proposed that the mechanistic pathway starts with the formation of an azidohydrin derivative, followed by dehydration, the formation of a nitrene by nitrogen elimination and 1,3-migration of an ethyl group, and finally hydrolysis of the ethylamine derivative. The attempt to perform the Schmidt reaction with conc. hydrochloric acid instead of sulfuric acid led to the formation of *meso*-chlorinated products **P146a**–**f** [93].

**Scheme 50:**
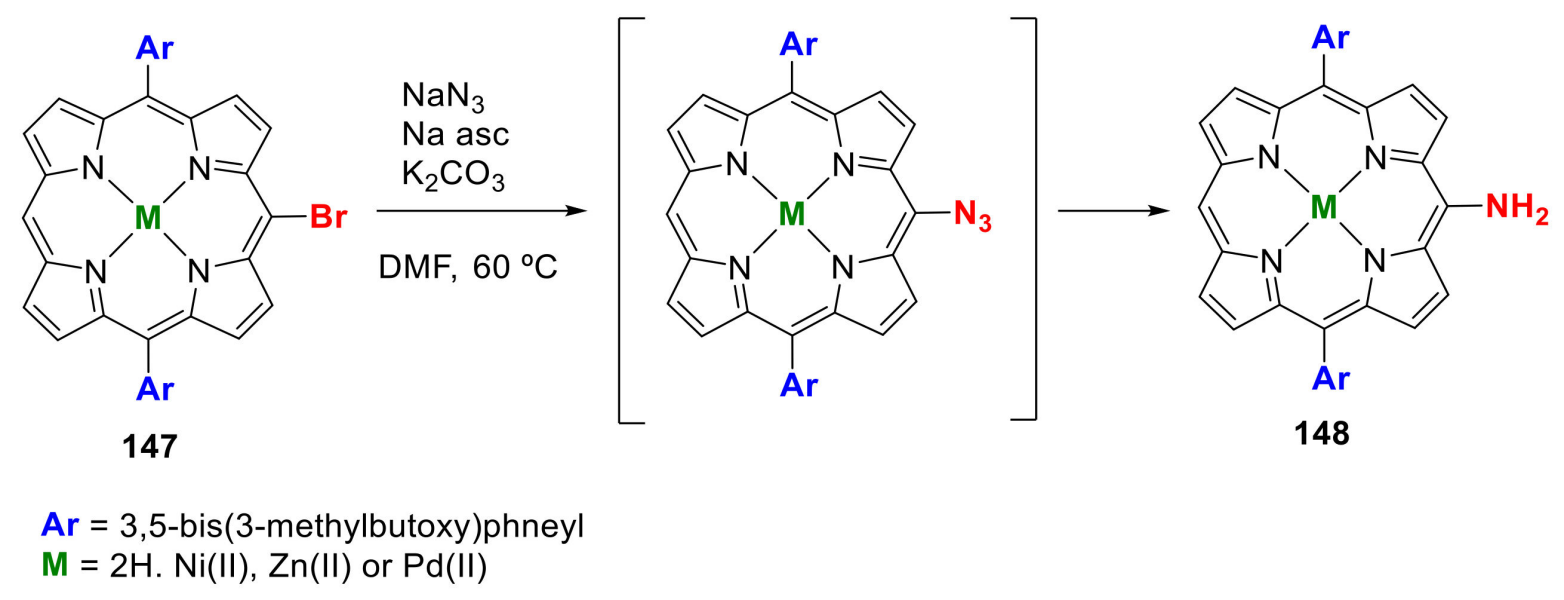
Preparation of *meso*-aminoporphyrin **P148** by reduction of the corresponding azide intermediate.

The treatment of 10-bromo-5,15-diarylporphyrins **P147** with sodium azide allowed for the preparation of the corresponding azidoporphyrin derivative as an intermediate, in high yields, through an aromatic nucleophilic substitution. The quickly in situ reduction of the azide intermediate by sodium ascorbate and K_2_CO_3_ afforded the amino derivative **P148** in yields ranging from 80% to 90% (Scheme 50) [94]. This procedure shows to be an excellent alternative to the previous method reported, involving the thermal decomposition of the azide (yields up to 44%).

**Figure 13:**
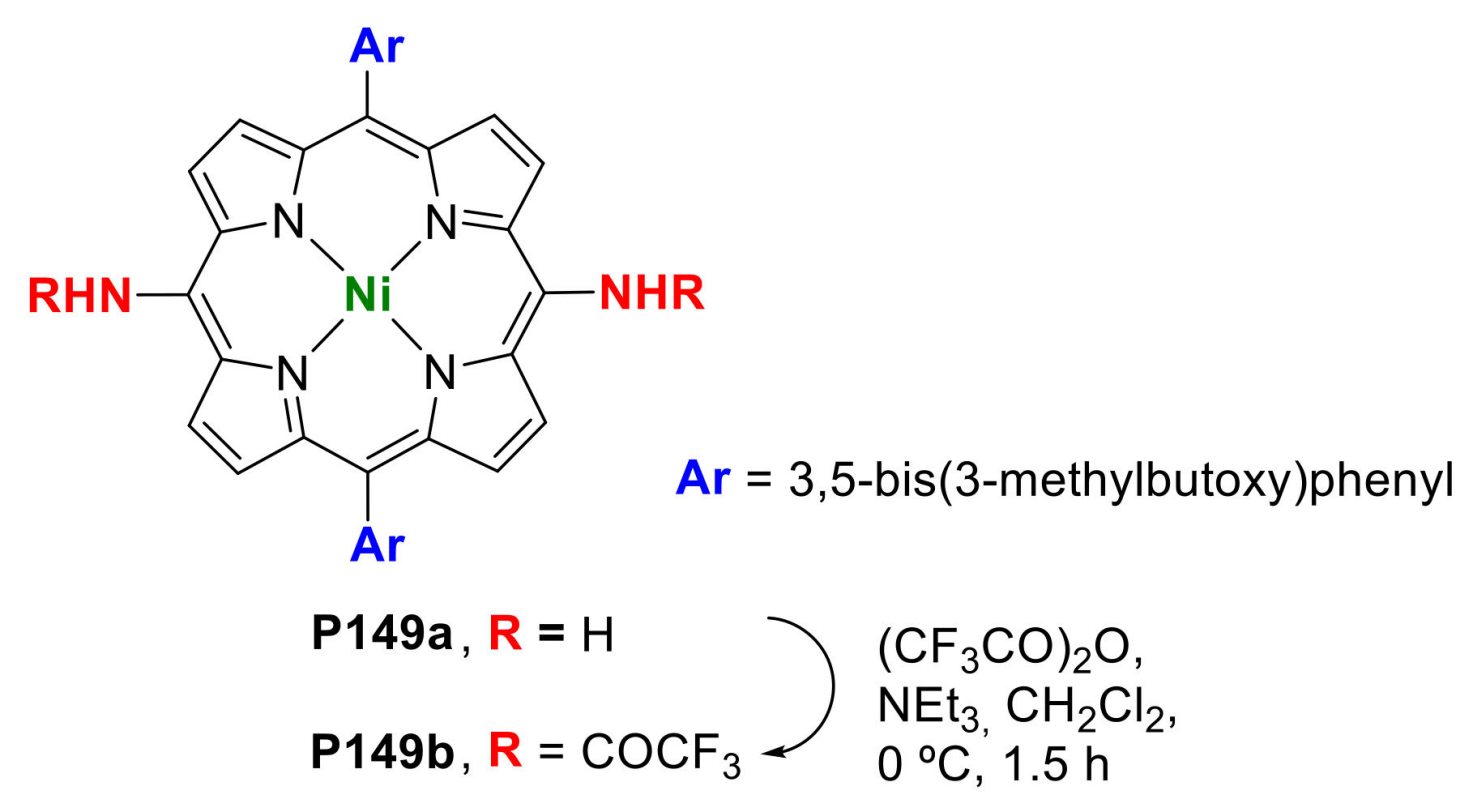
Structures of the Ni(II) complex of *meso*-substituted diaminoporphyrin **P149a** and the corresponding bis(trifluoroacetamide) **P149b**.

The authors extended this method to the free-base 5,15-dibromodiarylporphyrins, however only the mono-aminated derivative was isolated from the reaction (80% yield). When the S_N_Ar reaction was performed with the Ni(II) complex of the 5,15-dibromodiarylporphyrin the diaminated **P149a** was obtained; notwithstanding, this showed to be easily oxidized to the corresponding quinone derivative during purification. However, the crude of the Ni(II) complex of *meso*-substituted diaminoporphyrin **P149a** was successfully converted into the stable bis(trifluoroacetamide) **P149b** analogous (Figure 13) [94].

## 3. Catalytic Applications and Azide Transformations

Amongst several catalytic systems that have been reported for nitrene transfer reactions, some porphyrin metal complexes attracted attention as efficient catalysts. On the other hand, organic azides revealed to be potentially green resources to transfer nitrenes, since the only by-product formed throughout the generation of nitrenes from azides is dinitrogen. Alkene aziridination and C-H amination are among the most common uses for nitrene transfer reactions, although several other very promising applications have been recently explored [95,96,97,98,99,100,101,102,103,104]. Those most recent developments employing porphyrin metal complexes as efficient catalysts are summarized below.

**Scheme 51:**
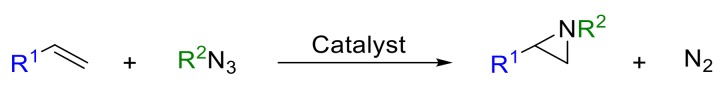
Synthesis of aziridines catalyzed by iron and ruthenium porphyrin catalysts.

Molecules containing an aziridine functional group are a versatile class of organic synthons due to the presence of a strained three-membered ring, which can be easily involved in ring-opening reactions and the aziridine functionality often shows interesting pharmaceutical and/or biological behaviors. Gallo and collaborators recently reviewed the most important results on the catalytic activity of iron and ruthenium porphyrin complexes in the aziridination of alkenes (Scheme 51) [105]. An interesting and straightforward two-step synthesis of *N*-arylaziridines starting from azides and anilines was efficiently achieved under continuous flow conditions, which was highlighted in the Gallo´s review [106].

**Scheme 52:**
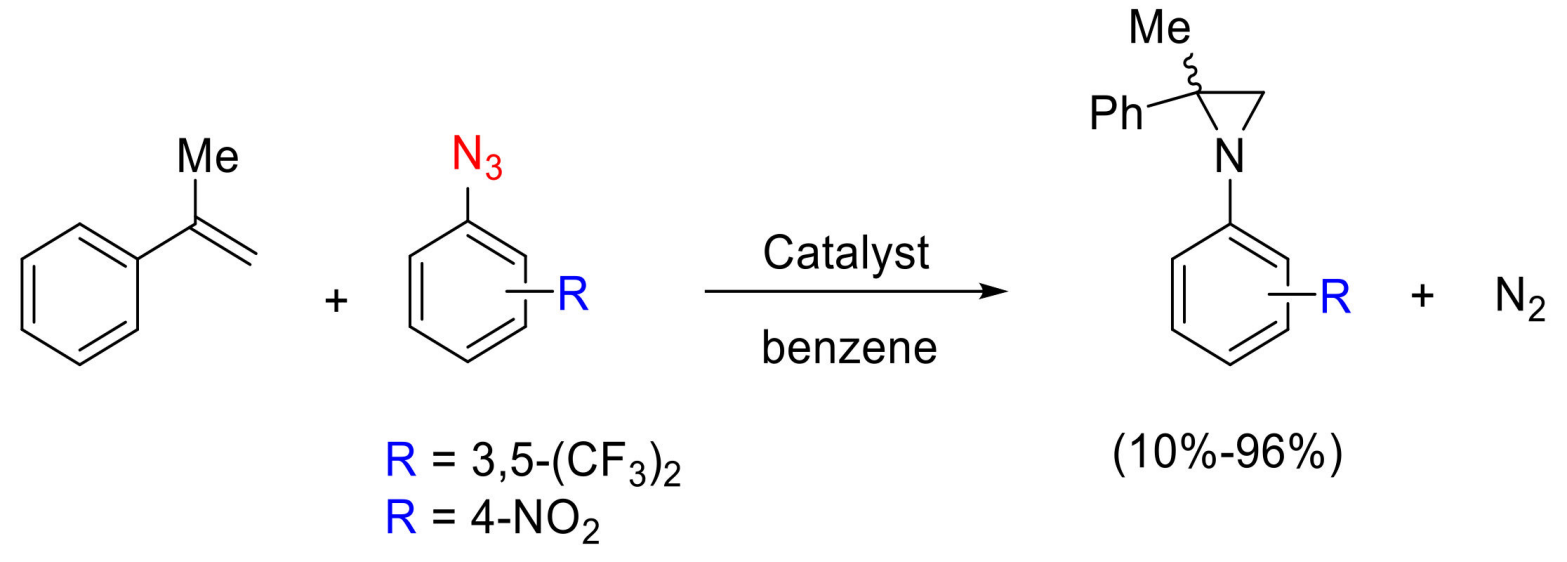
Aziridination of α-methylstyrene by either 3,5-bis(trifluoromethyl)phenyl azide or 4-nitrophenyl azide catalyzed by cobalt or ruthenium porphyrins.

Moreover, Gallo and collaborators reported the aziridination of α-methylstyrene with electron-poor aryl azides in the presence of cobalt or ruthenium porphyrins, a procedure which was accomplished under batch and continuous flow conditions (Scheme 52). Higher yields were registered for ruthenium porphyrins under batch conditions, although cobalt porphyrins showed higher efficiency under the used flow conditions [100,107].

The same group was able to prepare several iron and ruthenium glycoporphyrin complexes **P141** (Scheme 47) and reported their uses as catalysts in the aziridination of α-methylstyrene in the presence of several aryl azides. The yields obtained ranged from 10% to 96%, depending on the porphyrin metal complex and also on the substitution pattern at the phenyl ring of the azide [91].

**Scheme 53:**
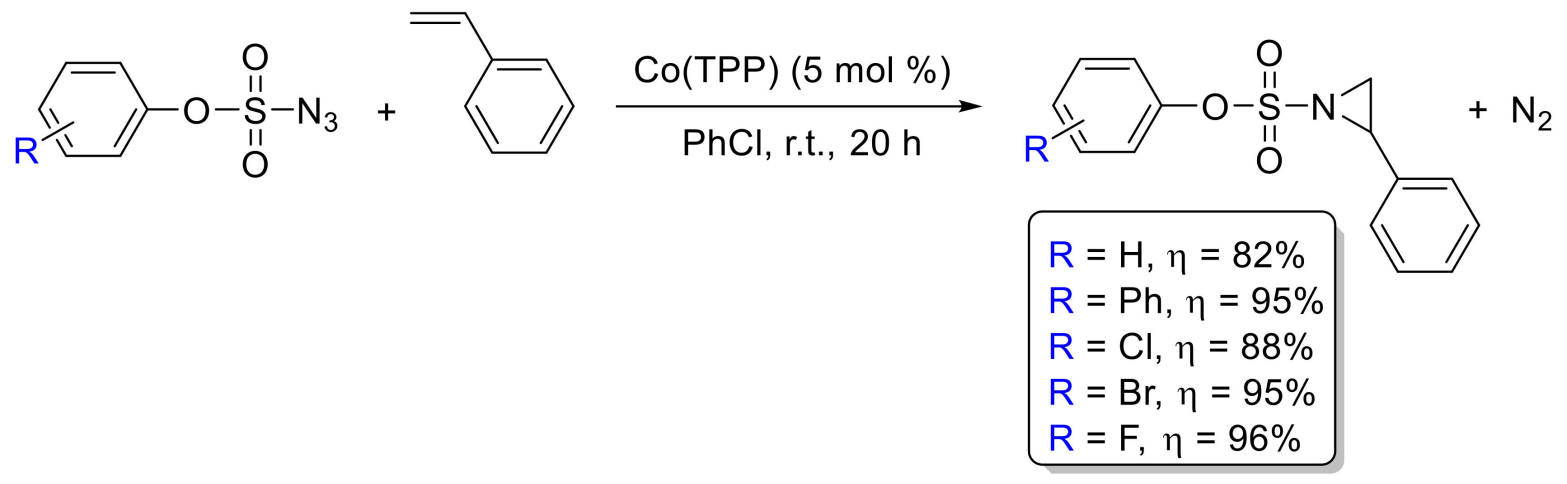
Aziridination of styrene with arylsulfonyl azides catalyzed by **Co(TPP)**.

Zhang and collaborators reported that aryloxysulfonyl azides (ArOSO_2_N_3_) can be successfully activated by the **Co(TPP)** catalyst, at room temperature, for selective aziridination of alkenes (Scheme 53). This **Co(TPP)**-based catalytic aziridination showed to be adequate for the reactions of several olefins and aryloxysulfonyl azides, thus generating *N*-aryloxysulfonyl aziridine derivatives in good to excellent yields [108].

**Scheme 54:**
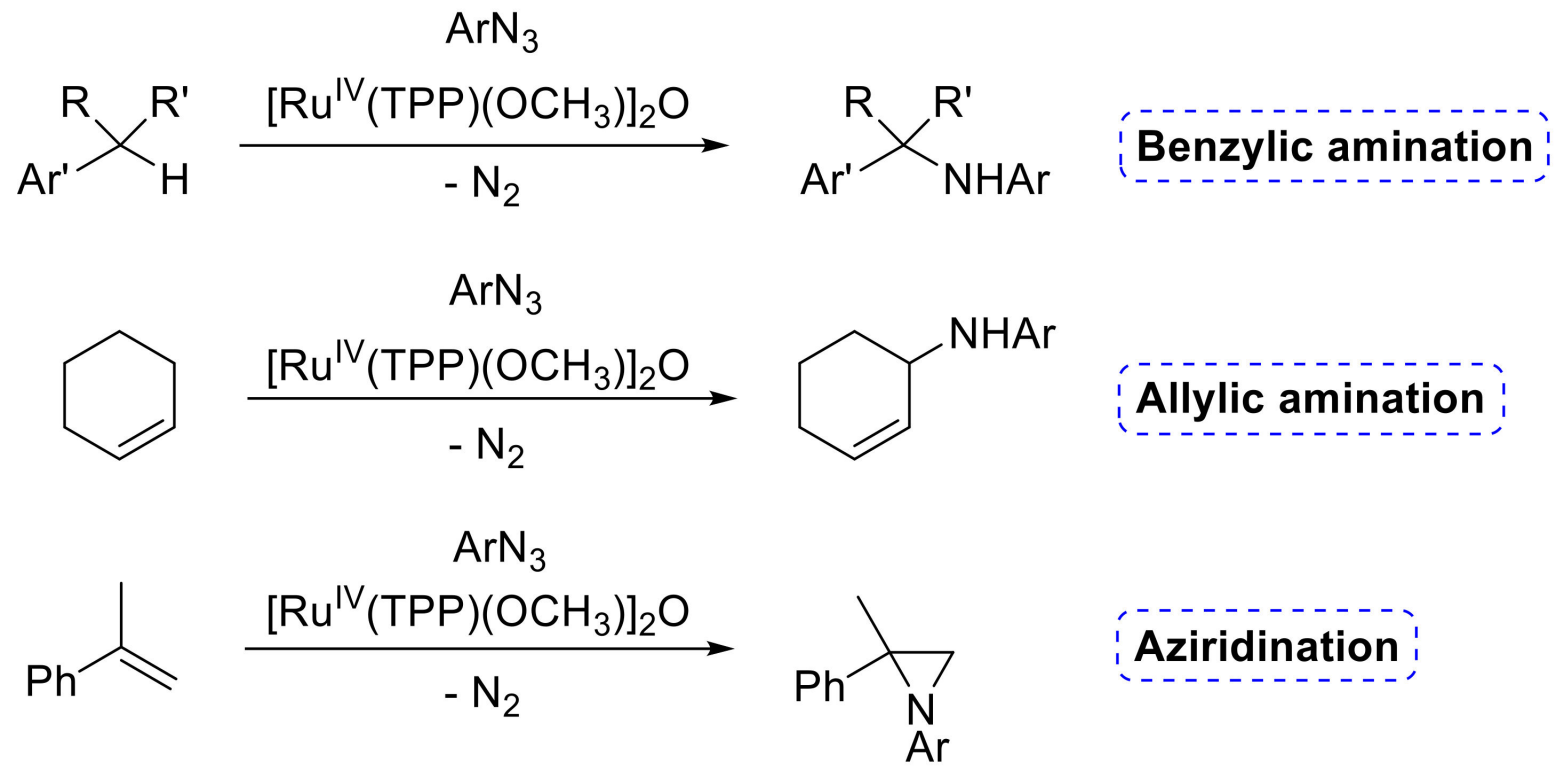
Nitrene transfer reactions catalyzed by [Ru^IV^(TPP)(OCH_3_)]_2_O.

Besides aziridination, the ruthenium(IV) µ-oxo porphyrin complex [Ru^IV^(**TPP**)(OCH_3_)]_2_O was shown to be catalytically active in allylic and benzylic amination by nitrene transfer reactions using aryl azides (ArN_3_) as the nitrene sources (Scheme 54). The catalytic efficiency of [Ru^IV^(**TPP**)(OCH_3_)]_2_O was similar to that of Ru^II^(**TPP**)CO [109]. Most recently, Gallo and collaborators reported the synthesis of some glycoporphyrin metal complexes such as cobalt(II), ruthenium(II) and iron(III), which were studied as catalysts for C-H bond aminations of ethylbenzene and other hydrocarbons by organic azides. In general, ruthenium(II) and iron(III) glycoporphyrin complexes have demonstrated to act with the best catalytic efficiency [110].

**Scheme 55:**
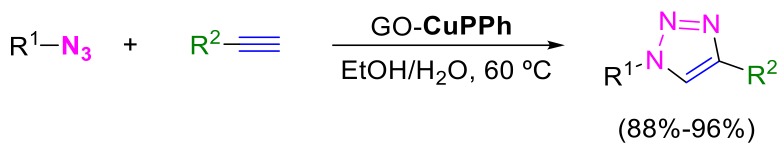
Synthesis of 1,4-disubstituted 1,2,3-triazoles in the presence of the heterogeneous catalyst (GO-**CuPPh**).

A heterogeneous catalyst was prepared by the nucleophilic reaction of the copper(II) complex of 5,10,15,20-tetrakis(4-aminophenyl)porphyrin (**CuPPh**) with the carboxyl groups at the edges of graphene oxide (GO), thus giving rise to the covalently cross-linked **CuPPh** catalyst, GO-**CuPPh**. The catalytic activity of GO-**CuPPh** was evaluated in the synthesis of 1,4-disubstituted 1,2,3-triazole derivatives by the reaction of several aryl azides with some terminal alkynes. Under the best conditions reported, the aryl azide, the terminal alkyne and the GO-**CuPPh** catalyst (0.5 mol%), in a mixture of H_2_O/EtOH (1:1), were irradiated at 60 °C for 5–30 min under ultrasonic conditions, affording the corresponding 1,4-disubstituted 1,2,3-triazoles in good to excellent yields (Scheme 55). Interestingly, all aryl azides carrying either electron-donating or electron-withdrawing groups reacted successfully and both aromatic and aliphatic terminal acetylenes gave rise to the corresponding 1,4-disubstituted 1,2,3-triazoles in excellent yields. The reusability of this catalyst (GO-**CuPPh**) was also studied; similar catalytic activity was obtained after the first (96%) and the fifth cycle (90%), hence with no significant loss of activity after recycling the catalyst [111].

**Scheme 56:**
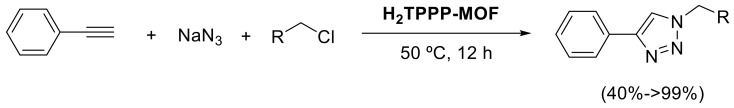
Schematic representation of the azide-alkyne click reactions catalyzed by the heterogeneous porphyrin-MOF catalysts.

**Figure 14:**
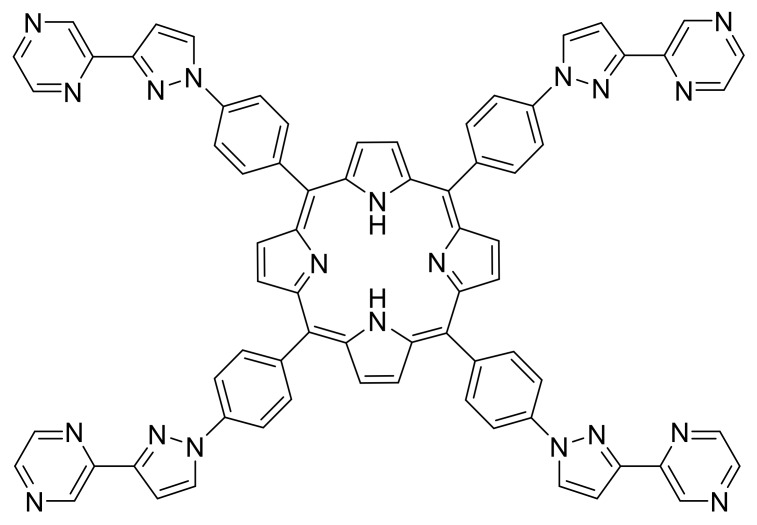
Structure of 5,10,15,20-tetrakis(4-(3-(pyrazin-2-yl)-1*H*-pyrazo-1-yl)phenyl)porphyrin (**H_2_TPPP**).

By using the 5,10,15,20-tetrakis(4-(3-(pyrazin-2-yl-1*H*-pyrazo-1-yl)phenyl)porphyrin (**H_2_TPPP**) (Figure 14), two mixed-valent Ag(I,II)- and Cu(I,II)-organic networks (MOFs) were synthesized and their catalytic activities evaluated in azide-alkyne cycloaddition reactions. In a typical procedure, benzyl chloride (or 3-methylbenzyl chloride, 2-fluorobenzyl chloride, and 4-methylbenzyl chloride), phenylacetylene and sodium azide were dissolved in methanol/water (4:1) and then the catalyst (1 mol%) was added to the solution, which was heated at 50 °C for 12 h (Scheme 56). The mixture porphyrin/Cu(I,II) MOF catalyst exhibited higher catalytic activity for the azide-alkyne cycloaddition reactions (always affording >99% yield for the corresponding 1,2,3-triazoles) than the analogous porphyrin / Ag(I,II) MOF (benzyl chloride and 2-fluorobenzyl chloride gave rise only to 40% and 42% yields, respectively, whereas for 3-methylbenzyl chloride or 4-methylbenzyl chloride no product was detected). Concerning the reusability of the porphyrin / Cu(I,II) MOF catalyst, a small decrease in the activity was registered after five runs (from 90% to 99%) [112].

**Scheme 57:**
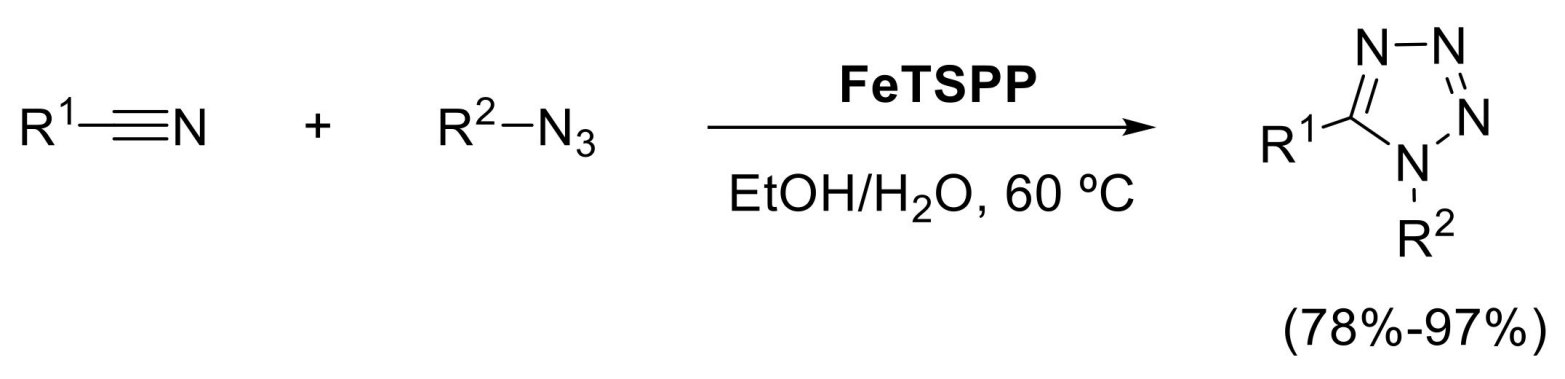
Synthesis of tetrazole derivatives in the presence of the water-soluble **FeTSPP** catalyst.

The water-soluble 5,10,15,20-tetrakis(4-sulfonatophenyl)porphyrin)iron(III) chloride (**FeTSPP**) was used as a catalyst for the synthesis of tetrazole and guanidinyltetrazole derivatives via [2 + 3] cycloaddition reaction between different nitriles and several azide derivatives in an aqueous medium (water/EtOH = 3:1) at 60 °C (Scheme 57). The reported 32 products were obtained in good to excellent yields (78%–97%), in short reaction times and the catalyst (0.5 mol%) could be used in four runs without loss of activity [113].

**Figure 15:**
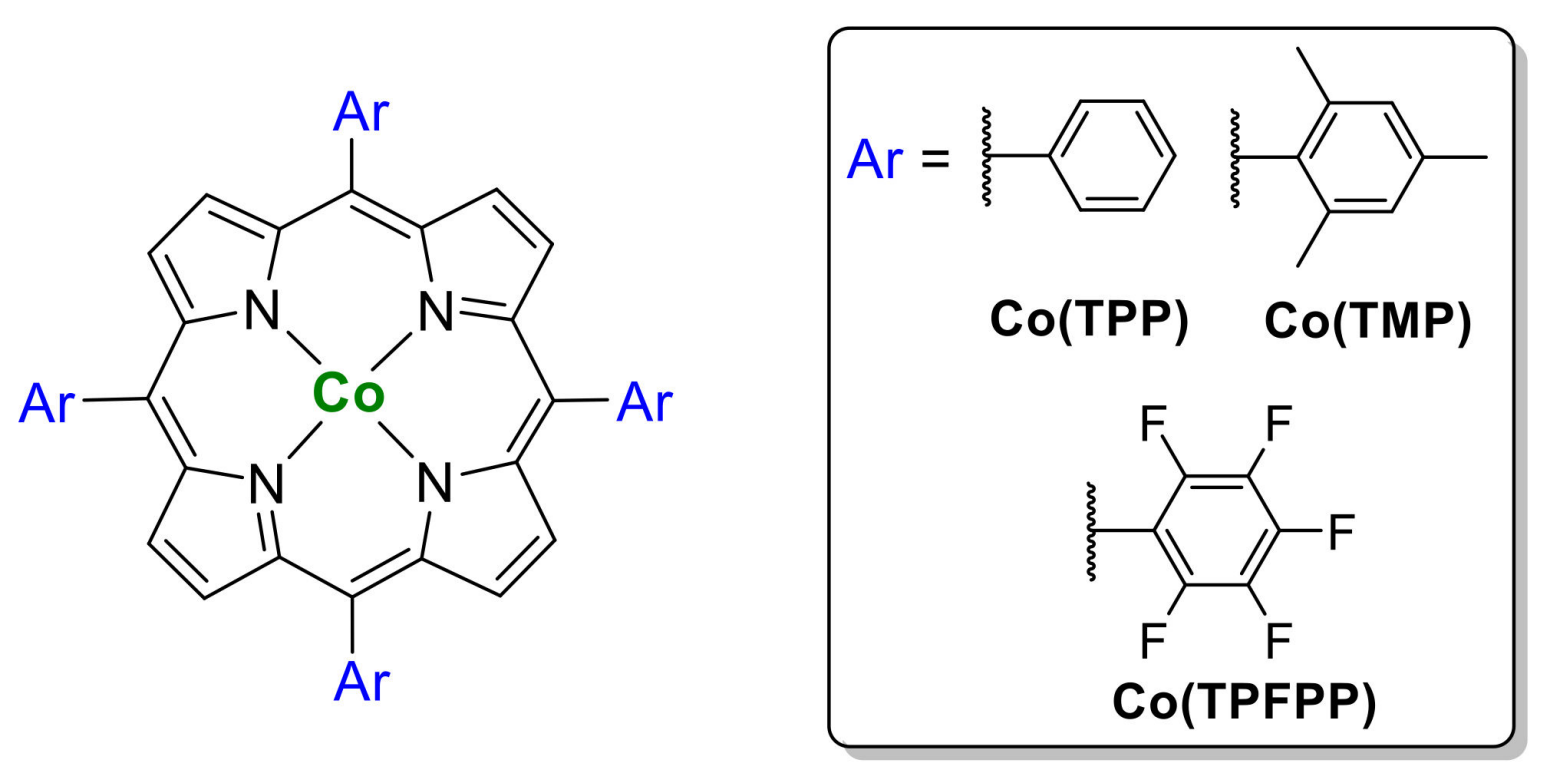
Cobalt(II) complexes used in the intramolecular ring-closing C-H bond amination study: cobalt(II) *meso*-tetraphenylporphyrin **Co(TPP)**; cobalt(II) *meso*-tetrakis(pentafluorophenyl)porphyrin **Co(TPFPP)**; cobalt(II) *meso*-tetramesitylporphyrin **Co(TMP)**.

**Scheme 58:**
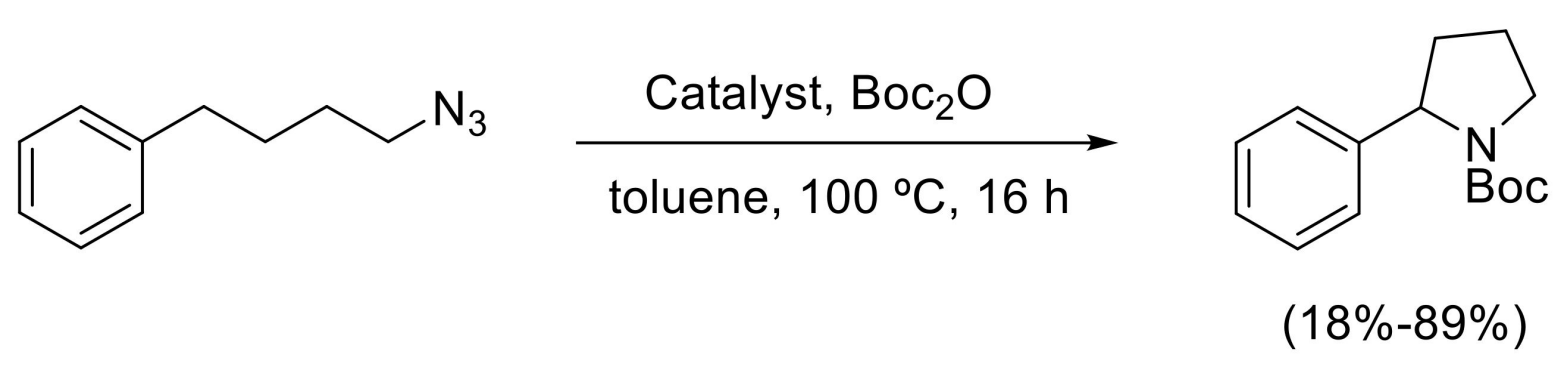
Catalytic intramolecular ring-closing C-H bond amination starting from (4-azidobutyl)benzene as substrate with formation of the corresponding pyrrolidine.

**Scheme 59:**
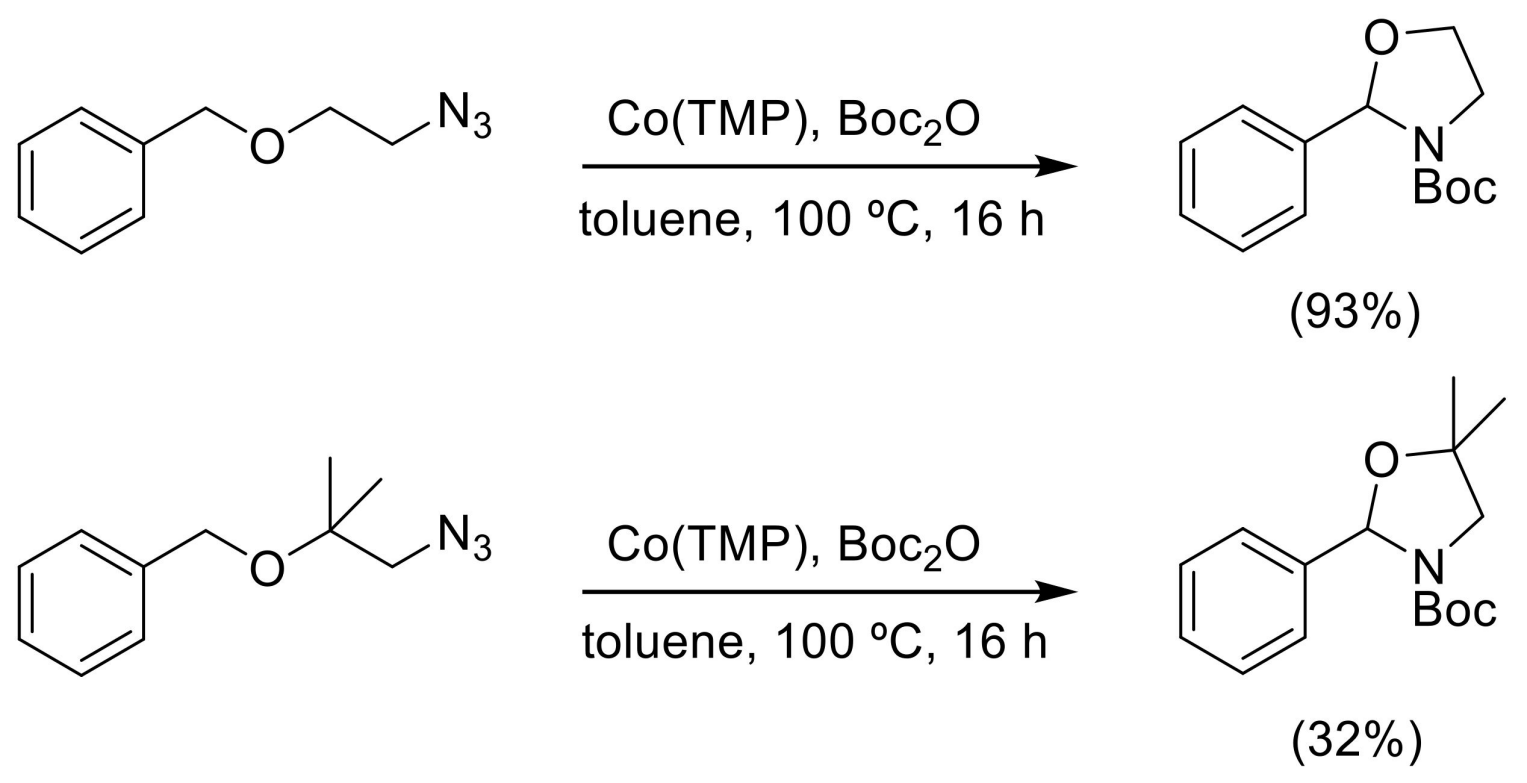
Catalytic formation of oxazolidines.

**Scheme 60:**
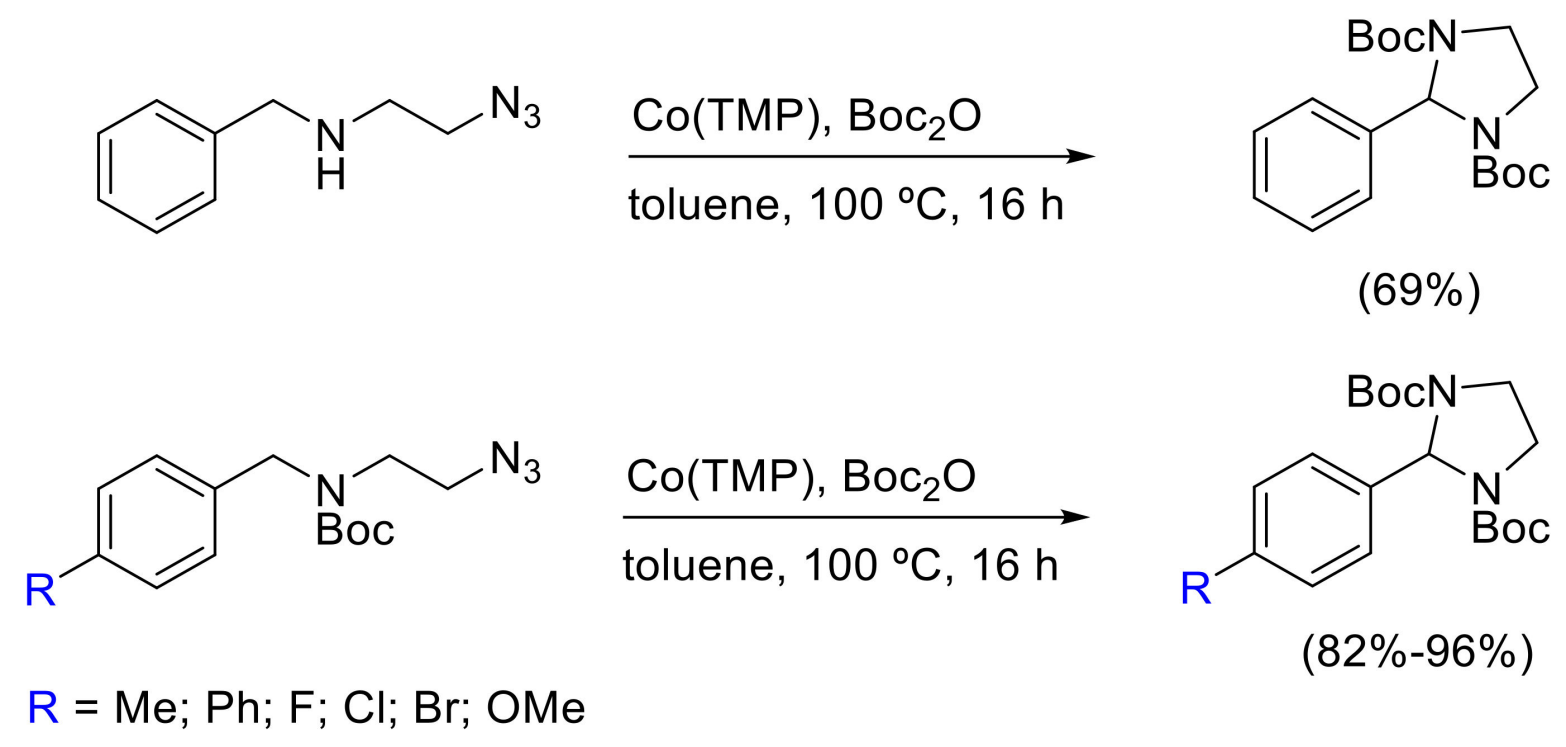
Catalytic formation of imidazolidines.

**Scheme 61:**
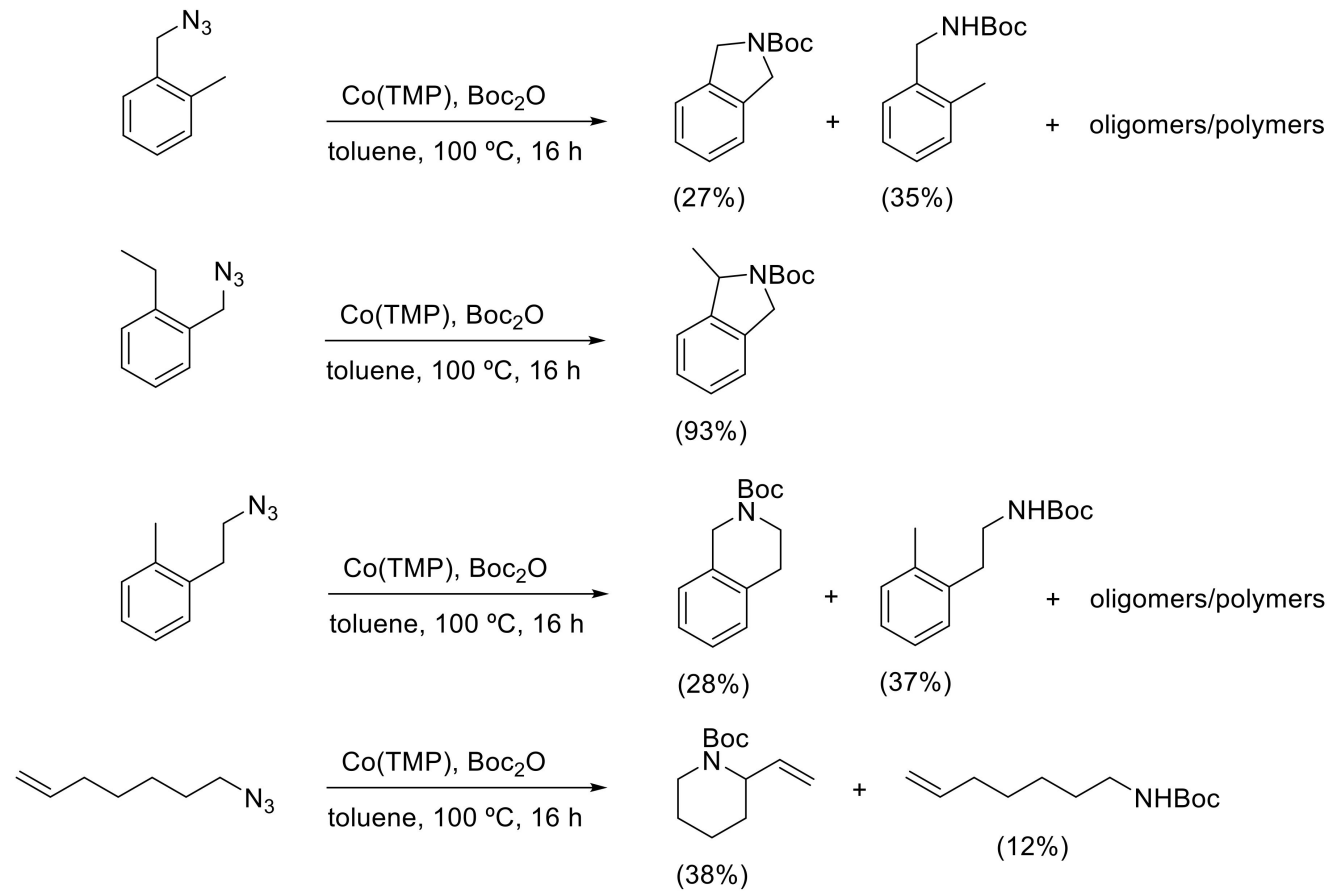
Application of the **Co(TMP)** catalyzed intramolecular ring-closing C-H bond amination reaction for the synthesis of other heterocycles.

As already stated the activation of organic azides by a metal-based catalyst allows selective amination reactions through the formation of active nitrene intermediate species [114]. The C-H bond amination reactions catalyzed by hemoproteins, iron porphyrins and phthalocyanines, also by non-heme-type iron complexes, as well as metal-organic framework-supported iron complexes, were recently reviewed and will not be explored further ([98] and references cited therein). Another review dealing with the use of ruthenium porphyrins to catalyze hydrocarbon amination reactions showed that this class of porphyrin catalysts are able to activate organic azides in the synthesis of several aza-derivatives. Various synthetic methodologies have been reported there, concerning ruthenium porphyrins as catalysts for nitrene transfer reactions in C-H bond amination reactions [114]. Intramolecular ring-closing C-H bond amination enables direct synthesis of various *N*-heterocycles from aliphatic azides. Pyrrolidines (Scheme 58), oxazolidines (Scheme 59), imidazolidines (Scheme 60), isoindolines and tetrahydroisoquinoline (Scheme 61) were obtained by de Bruin and collaborators in good to excellent yields in a single reaction step catalyzed by cobalt(II) porphyrins (Figure 15). In the absence of an amine trapping agent, complete recovery of the starting material was observed after 16 h at 100 °C. The use of di-*tert*-butyldicarbonate (Boc_2_O) significantly enhances the reaction rate by preventing competitive binding of the resulting amine and the use of toluene gave better results than benzene. Marked improvements both in yield and TON were observed using **Co(TMP)** as catalyst in the formation of pyrrolidines, and this was the Co(II)porphyrin complex of choice for the other reactions reported. Furthermore, the enantioselective ring-closing amination reaction was achieved when using a chiral cobalt-porphyrin catalyst with four (1*S*)-(−)-camphanic ester substituents in the second coordination sphere. Low enantioselectivities (29% *ee* at 100 °C and 46% *ee* at 80 °C) were attained for the ring-closure of (4-azidobutyl)benzene [99]. Recently, the same group reported the first example of the use of a cobalt(II) corrole complex in nitrene transfer reactions, more precisely in the ring-closing C-H amination of an aliphatic azide in the presence of Boc_2_O, giving the corresponding Boc-protected pyrrolidine (Scheme 58) [115]. Moreover, Che and collaborators described the synthesis of an iron(III) *N*-heterocyclic carbene (NHC) porphyrin complex, [Fe(III)(TDCPP)(IMe)_2_]I (**TDCPP** = *meso*-tetrakis(2,6-dichlorophenyl)porphyrinato; IMe = 1,3-dimethylimidazol-2-ylidene), which was used to catalyze the intramolecular amination of C-H bonds with a wide range of alkyl azides in the presence of Boc_2_O and under thermal and microwave-assisted conditions [116].

**Scheme 62:**
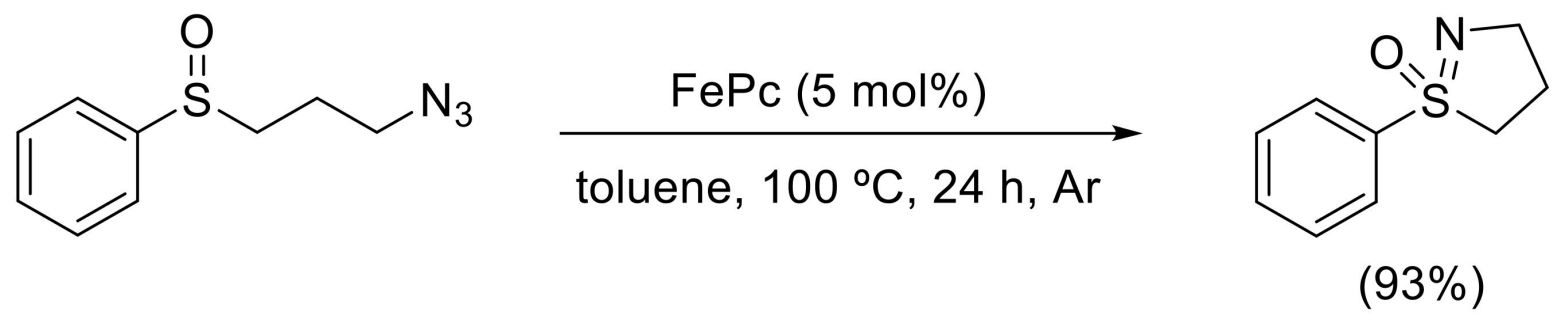
**FePc** catalyzed intramolecular imidation of (3-azidopropyl)sulfinylbenzene.

A broad and atom-economical method for the preparation of cyclic sulfoximines in up to 98% yield has been established by Bolm and collaborators via intramolecular imidation reactions of azido-substituted sulfoxides as nitrene precursors (Scheme 62). The catalytic process is based on a commercially available Fe(II) phthalocyanine (**FePc**) as catalyst for these for ring-closing sulfur imidations and exhibits large functional group tolerance. Toluene was chosen as the solvent and the yields were higher at 100 °C [117].

**Scheme 63:**
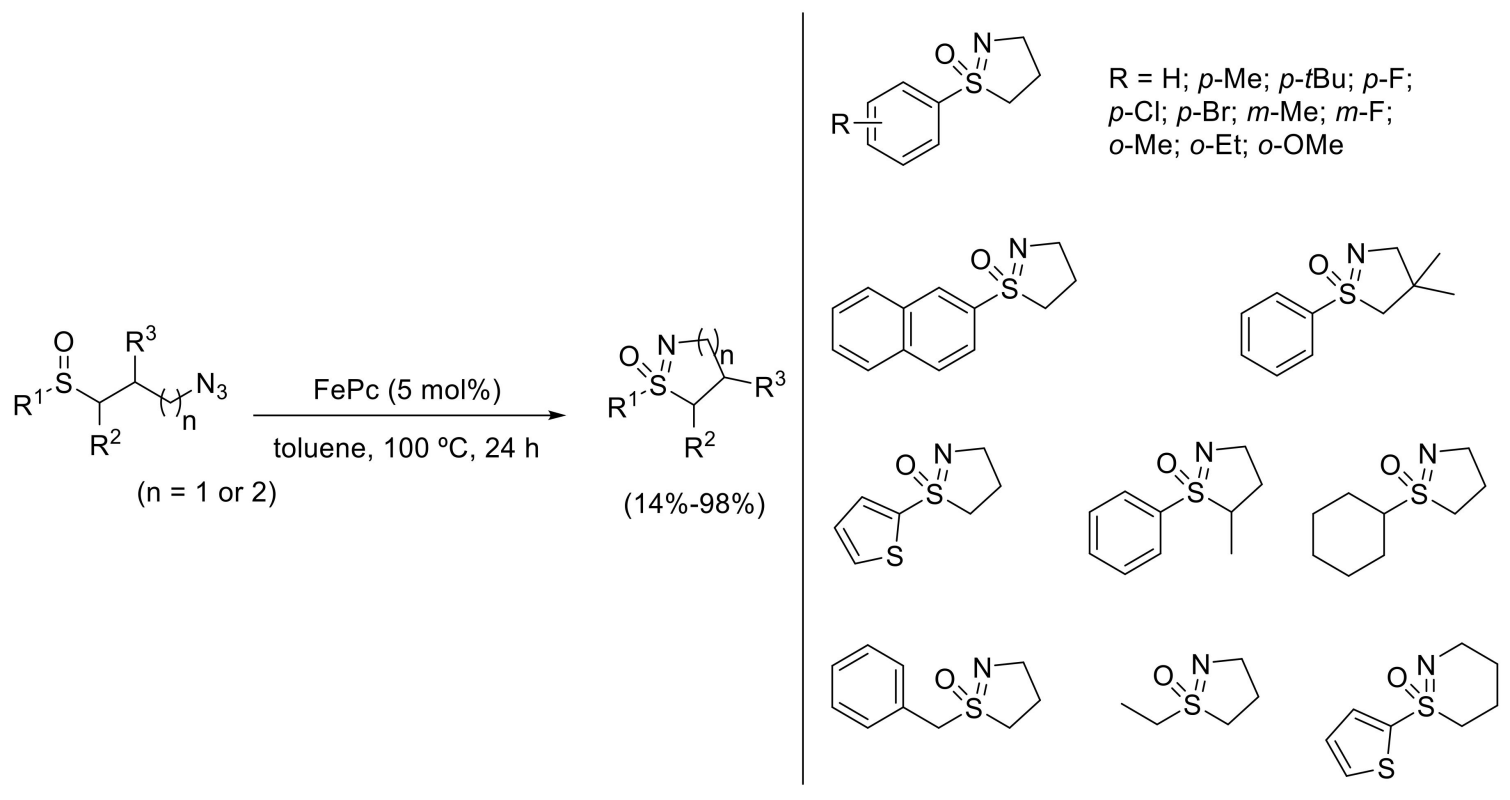
**FePc** catalyzed intramolecular imidation of several 3-azidosulfoxides and one 4-azidosulfoxide.

Under the best conditions studied and using 5 mol% of catalyst, several 3-azidoalkyl sulfoxides underwent intramolecular imidation, giving rise to the corresponding cyclized products with yields up to 98% (Scheme 63) [117].

**Scheme 64:**
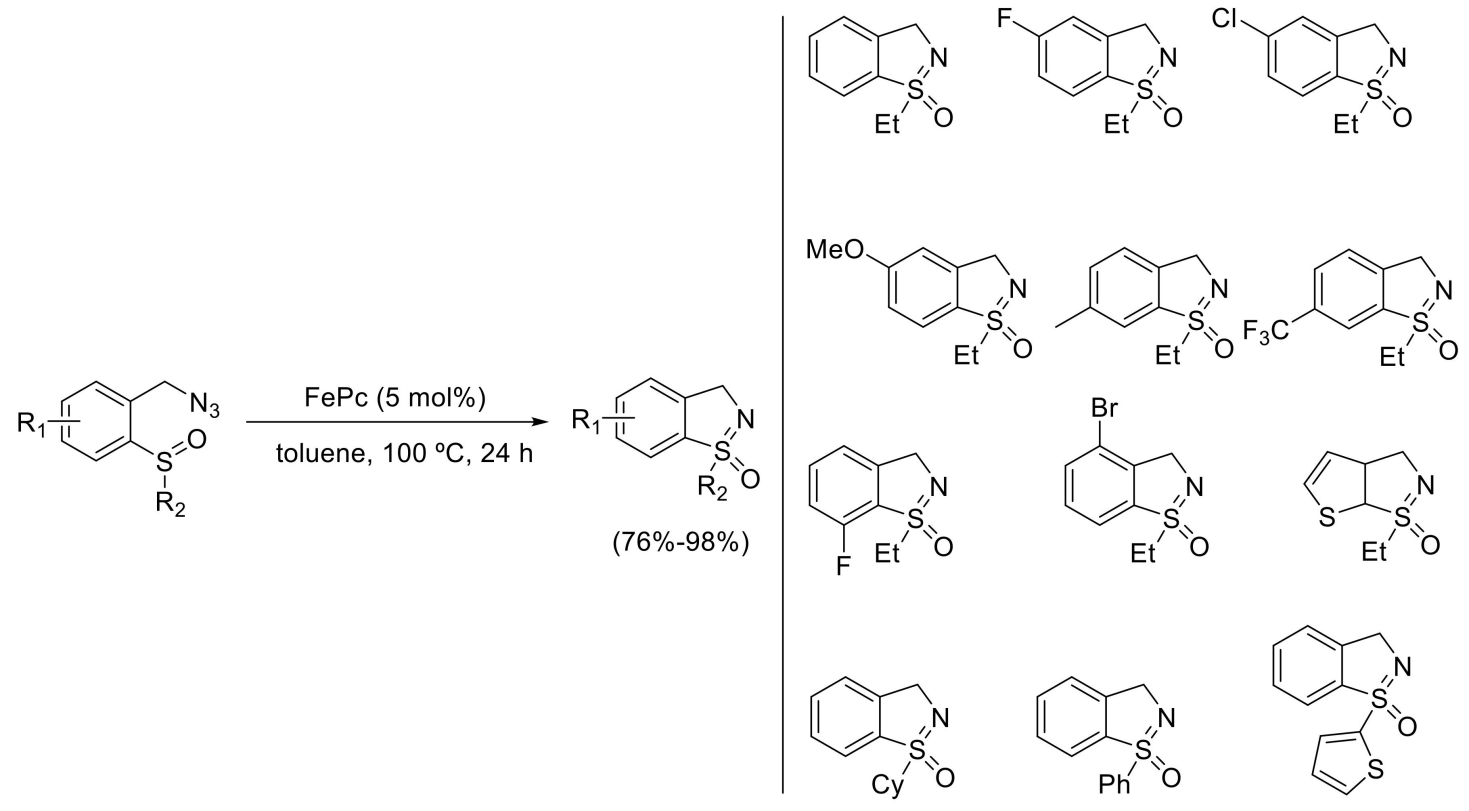
**FePc** catalyzed intramolecular imidation providing several benzo[*d*]isothiazole 1-oxides.

Starting from aryl sulfoxides with benzylic azido groups, the corresponding intramolecular imidation products were also obtained with yields for benzo[*d*]isothiazoles ranging from 76%–98% (Scheme 64) [117].

**Scheme 65:**
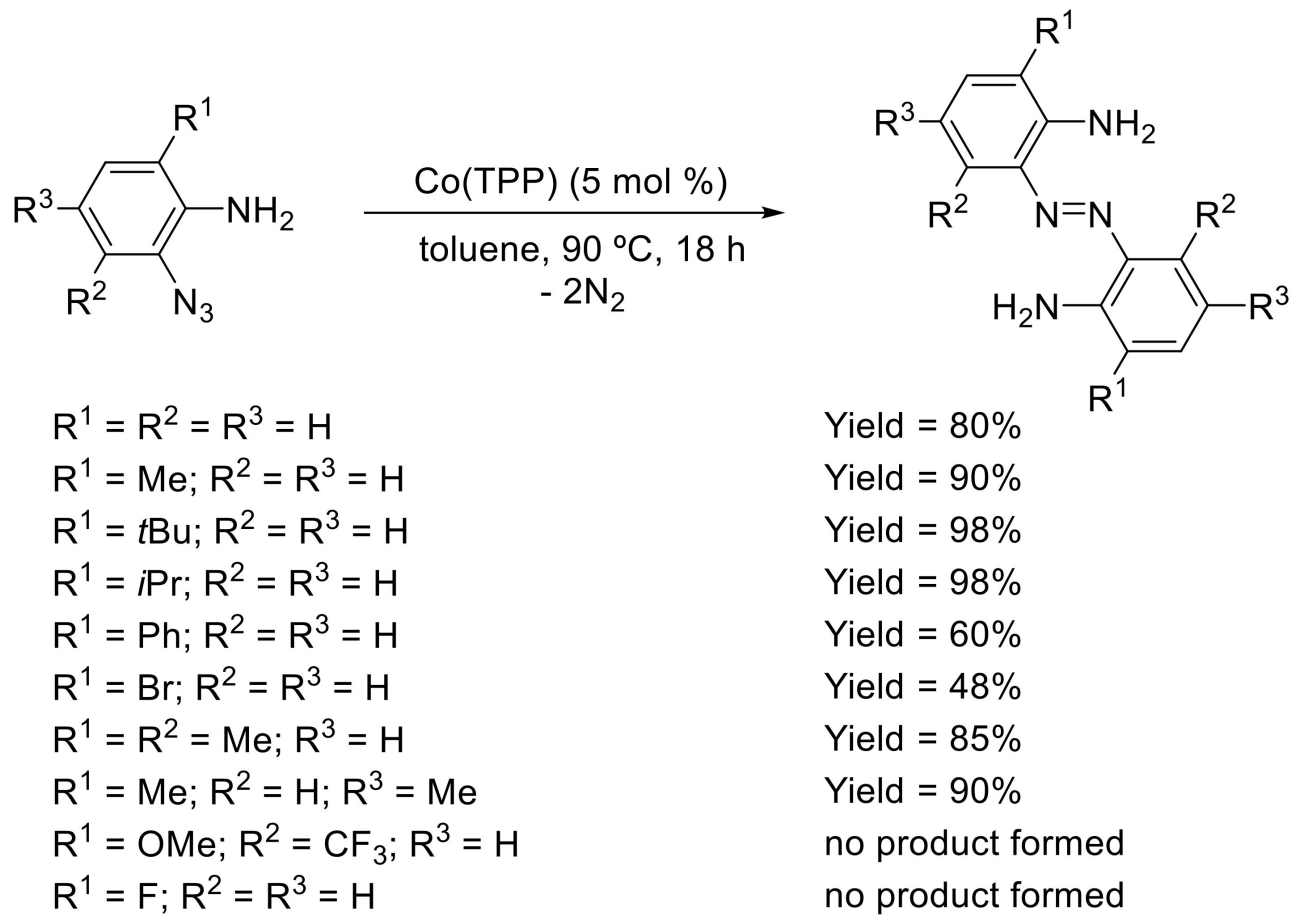
Synthesis of *o*-aminoazobenzenes catalyzed by the commercially available **Co(TPP)**.

A general method for the catalytic synthesis of *o*-aminoazobenzenes based on the commercially available Co(II) *meso*-tetraphenylporphyrin **Co(TPP)** was recently disclosed, which consists in a formal dimerization of two phenyl azides with simultaneous loss of two molecules of dinitrogen (Scheme 65) [118].

**Scheme 66:**
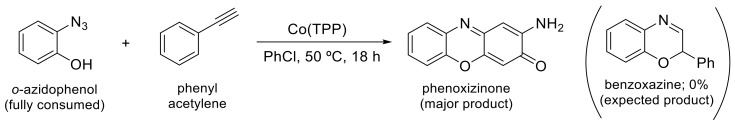
Formation of phenoxyzinone instead of benzoxazine from *o*-azidophenol in the presence of **Co(TPP)**.

In the pursuit of using 2-OH and 2-NH_2_ substituted aryl azides in Co(II) porphyrin catalyzed nitrene transfer reactions, de Bruin and collaborators found that from the *ortho*-hydroxy aryl azides both phenoxyzinones and benzoxazines could be synthesized in high yields, whereas from the 2-amino aryl azide substrates, azobenzenes were obtained as main products. So, the actually observed resulting compounds contradict the expected cobalt mediated catalytic coupling of nitrenes to alkynes or alkenes (Scheme 66). When the reaction was carried out in neat alkyne (thus using an excess of the alkyne), almost exclusive formation of phenoxyzinone was obtained [101].

**Scheme 67:**
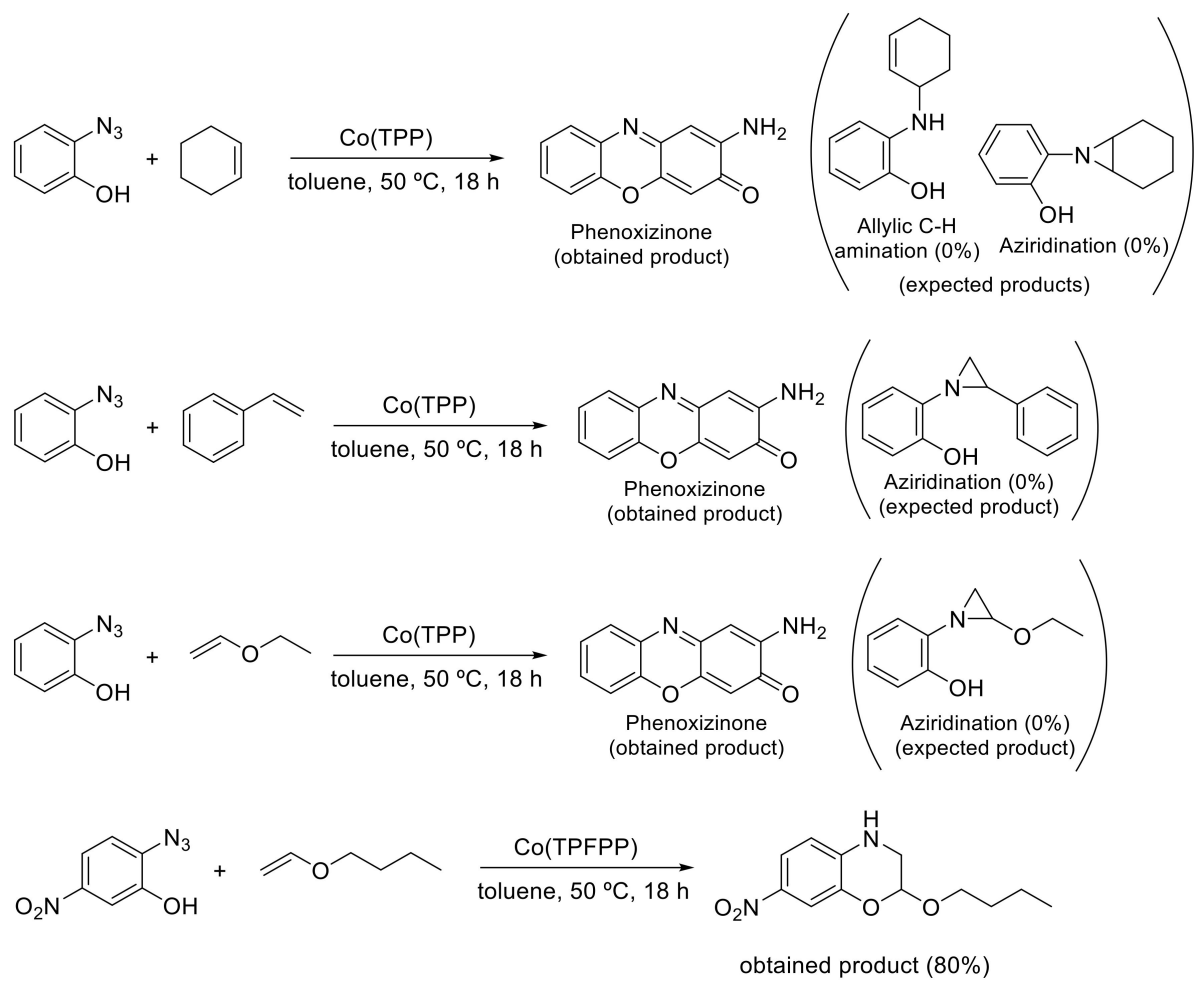
Formation of phenoxyzinone from *o*-azidophenol in the presence of alkenes and catalyzed by cobalt porphyrins.

Moreover, the aziridination of alkenes such as cyclohexene, styrene and 1-etoxyethene was also tried with *o*-azidophenol, although only phenoxyzinone was identified again as the major product. The authors were able to run this reaction with 2-azido-5-nitrophenol in the presence of **Co(TPFPP)** since **Co(TPP)** was inactive in this case, and the phenoxyzinone was not obtained in any case (Scheme 67) [101].

## 4. Corrole Macrocycles

Corroles are aromatic tetrapyrrolic macrocycles related to porphyrins but having only three methine bridges. Since 1999, the availability of these contracted macrocycles through facile synthetic strategies [119,120,121,122] allowed the scientific community to explore them successfully in different fields like catalysis, medicine (biomedical imaging, MRI contrast, cancer PDT, PDI of microorganisms, etc.) and in the design of photovoltaic materials and devices [123]. Among the different strategies used in the functionalization/post-functionalization of easily available corroles to obtain target molecules, the CuAAC approach is being successfully used. New corrole derivatives linked to other corroles [124], to porphyrins [125,126] and to BODIPYs [127] were successfully obtained, as recently reviewed by some of us [123]. In this section, only a few publications are being considered to illustrate the impact of the CuAAC approach in the functionalization of corroles.

**Figure 16:**
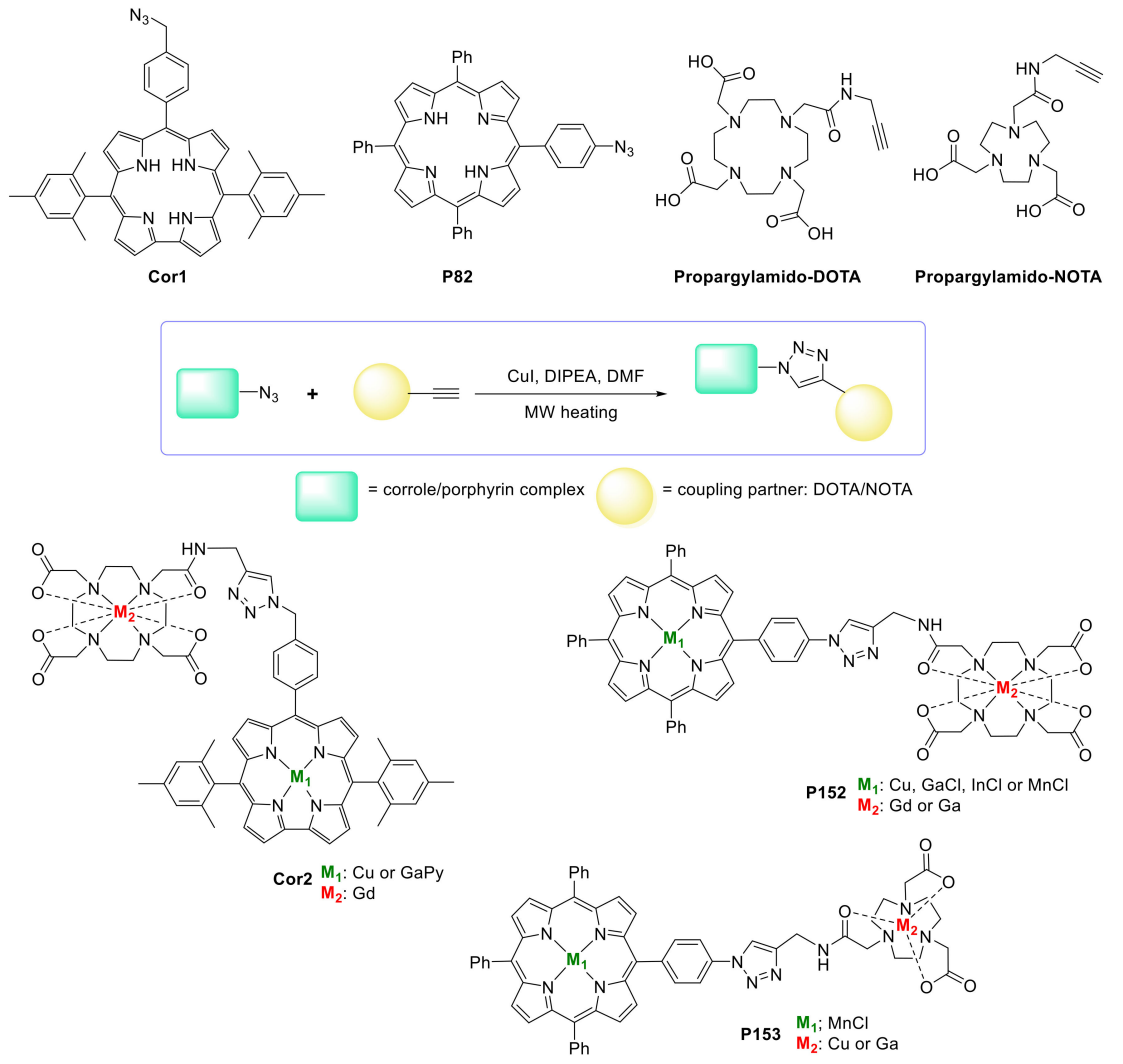
Structure of the heterobimetallic complexes **Cor2**, **P152** and **P153**, and of the free-base precursors used in their construction.

For instance, in 2015, Desbois and co-workers selected this approach as a convenient and rapid synthetic access to heterobimetallic complexes based on corroles (**Cor 2**) and on porphyrins (**P152** and **P153**) for medical imaging studies [128]. In the selection of the metal ions, authors took into account the potentiality of the Mn and Gd corrole complexes in the enhancement of contrast for MRI applications and of the “cold” metals complexes (Cu, Ga, In) for radionuclear imaging applications. The cycloaddition reactions were performed using azide **Cor1** or **P82** and the propargylamido-DOTA or propargylamido-NOTA in the presence of CuI and DIPEA under microwave irradiation. The expected complexes were obtained in yields ranging from 26% to 80% (Figure 16). Preliminary relaxivity assays seemed to indicate that these bimodal complexes are promising contrast agents in MRI.

**Scheme 68:**
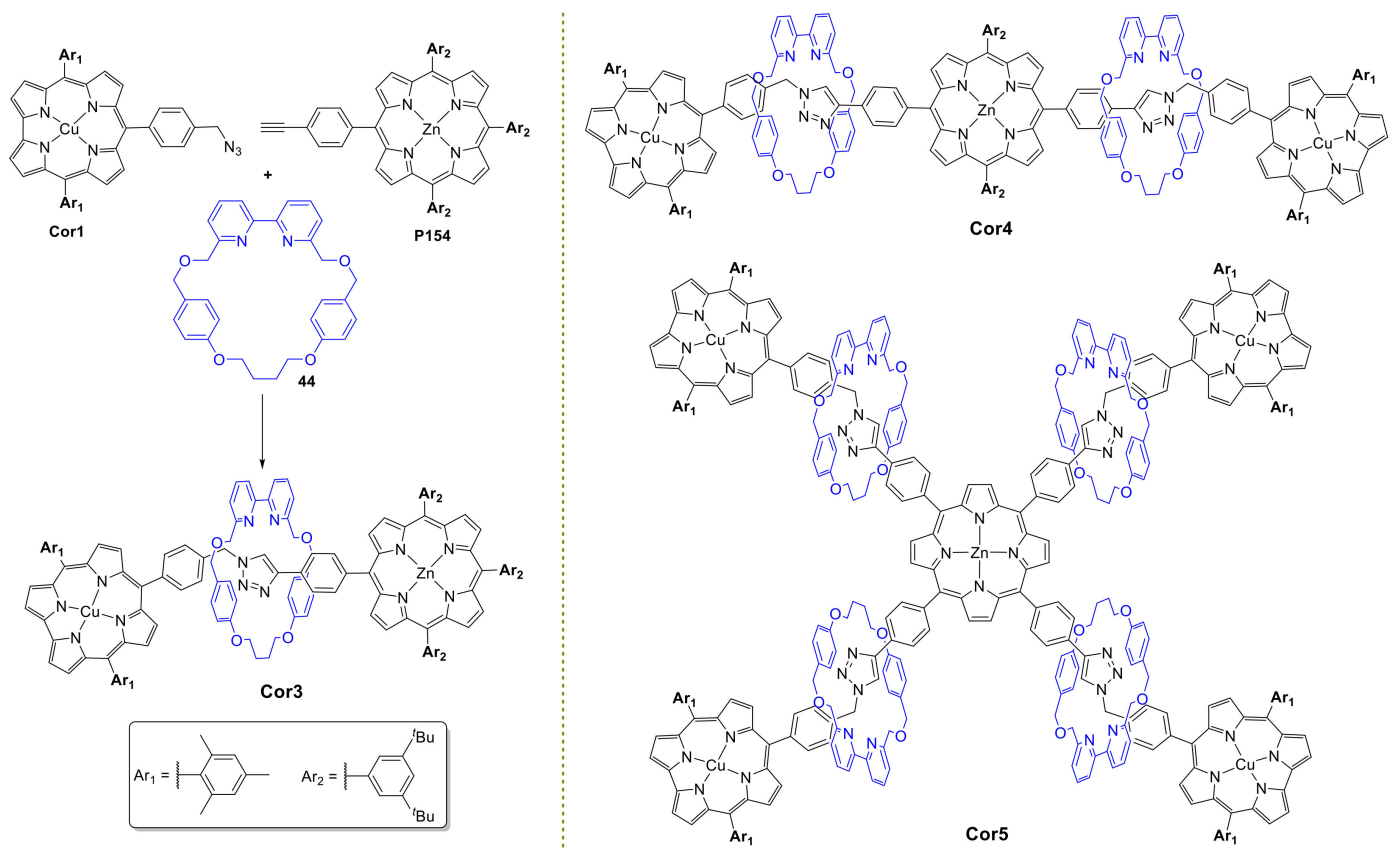
Strategy used to obtain porphyrin-corrole dyad **Cor3** and structures of the [3]rotaxane **Cor4** and [5]rotaxane **Cor5**.

In 2017, Ngo and co-workers explored the CuAAC approach to develop the rotaxanes based on porphyrins and corroles presented in Scheme 68 [129]. The reaction involved the azide corrole **Cor1**, the alkynyl porphyrin **P154** and the macrocycle **44** in the presence of CuI. The porphyrin-corrole dyad **Cor3** was obtained in 98% yield. When the method was extended to the synthesis of the triad [3]rotaxane **Cor4** and pentad [5]rotaxane **Cor5** the desired derivatives were also isolated in excellent yields (96% and 70% respectively). The study showed that these interlocked scaffolds are sterically hindered although the electronic properties of the axle components were not affected and so such compounds can be explored in the development of new catalysts and materials.

**Scheme 69:**
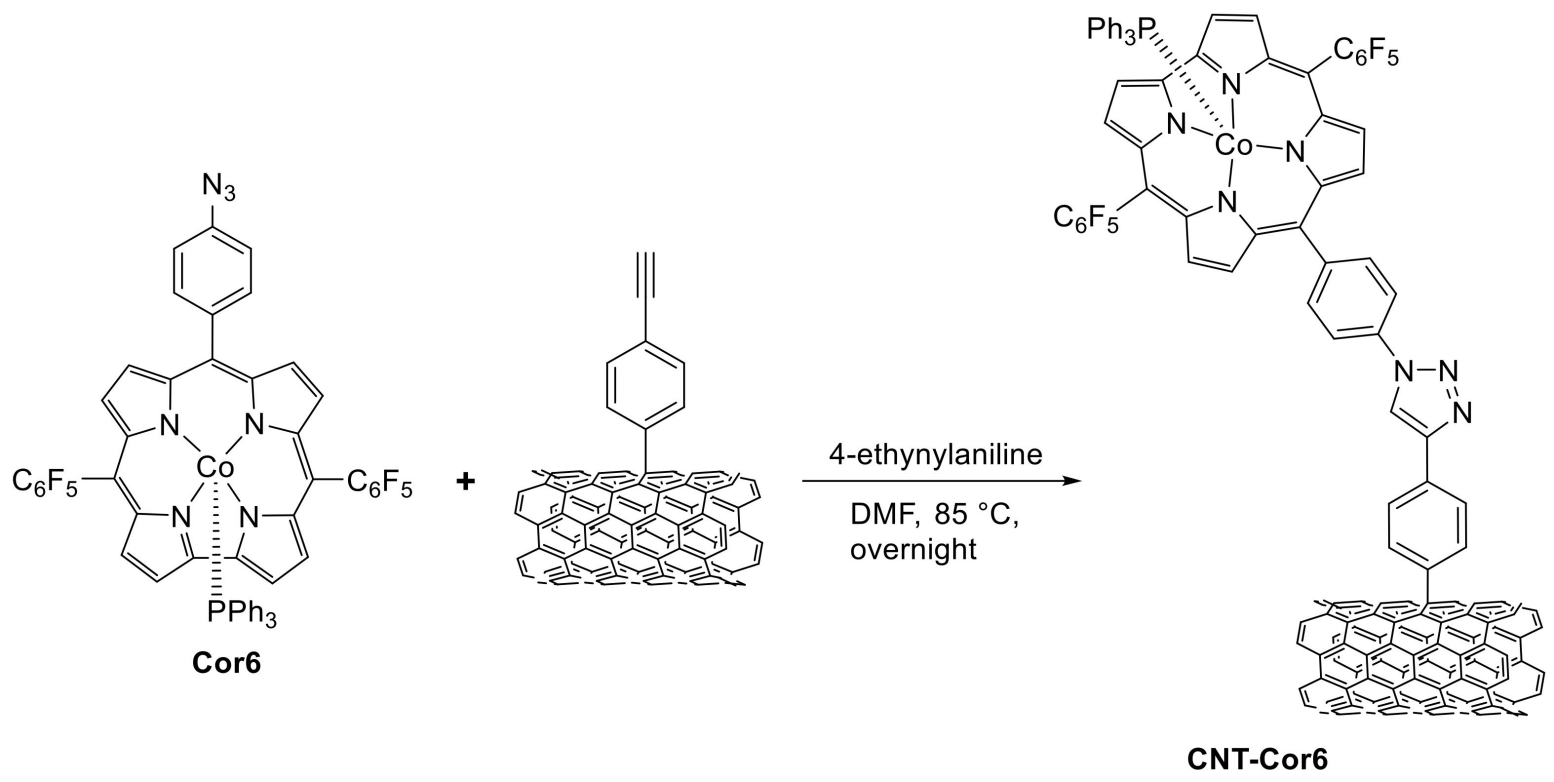
Synthetic strategy used to attach **Cor6** to carbon nanotubes.

In 2019, Cao and co-workers showed that the attachment of the cobalt complex of corrole **Cor6** as the azide component on carbon nanotubes functionalized with alkyne units leads to a system able to catalyze the 4e- reduction of O_2_ to H_2_O (Scheme 69) [130]. Typically, mononuclear Co complexes mediate the two-electron reduction of O_2_ to H_2_O_2_ but authors demonstrated that with an adequate covalent bond interaction to carbon nanotubes, mononuclear Co corroles can also become intrinsically active for the 4e- oxygen reduction reaction of O_2_ to H_2_O.

## 5. Final Remarks

Azides and pyrrolic derivatives are groups of compounds which play a key position in the synthesis of a large diversity of new derivatives, particularly those containing the triazole and porphyrin/corrole moieties. A wide range of potential applications are being put forward, particularly those related with light-harvesting, photovoltaic and photogalvanic devices, chemosensing, catalysis, and significantly on the medical side in MRI, drug delivery, hyperthermia therapy, in photodynamic therapy of cancer cells and in the photoinactivation of microorganisms.

The adaptation of the precursors to the final application requires some engineering in each component. In fact, different combinations with the alkynyl and azide components is in line with the excellence of the organic synthetic work developed under the context of CuAAC methods selected in this review. In most of the reported works the protection of the inner core of the macrocycle with a metal ion, mainly Zn(II) (which was easily removed by acid treatment), was required in order to avoid the coordination with Cu(II) ions. The used solvents comprised of general aqueous organic solvents but the use of only organic solvents does not seem to limit the applicability of the approach. Having in mind the significance of 1,2,3-triazoles in the medicinal and agricultural areas, it can be anticipated that the triazole unit might bring a synergic effect in the biological properties of the final target derivatives.

Besides the tremendous positive impact of the CuAAC reactions in organic synthesis, it must be stressed that they have some limitations. The main one is that CuAAC reactions are limited to terminal alkynes. These reactions lead to 1,4-disubstituted 1,2,3-triazoles but the regioisomeric 1,5-disubstituted 1,2,3-triazoles may be obtained selectively using the ruthenium(II)-catalyzed azide-alkyne cycloaddition. Other metal complexes (of silver, iridium, lutetium, scandium, and samarium) are also selective catalysts for the azide–alkyne cycloaddition reaction but they are rarely used [131]. Other methods for the synthesis of 1,2,3-triazole derivatives are also available in the literature. In some cases, they require very mild reaction conditions, including room temperature and aqueous media, and without the need to have alkyne derivatives.

Some metalloporphyrins are also excellent catalysts for nitrene transfer reactions leading to important transformations such as alkene aziridination and C-H amination suggesting that this partnership could be explored in more sophisticated systems.

Certainly, this scientific area in which this review is included will continue to be a “great target” in everyday objectives, mainly medicinal and industrial ones, of many researchers.

## Abbreviations

A-253	Human Epidermoid Carcinoma Cells
CB6	cucurbit[6]uril
CMPs	Conjugated Microporous Polymers
Conc.	concentrated
Co(TMP)	(5,10,15,20-tetramesitylporphyrinato)cobalt(II)
Co(TPFPP)	[5,10,15,20-tetrakis(pentafluorophenyl)porphyrinato]cobalt(II)
Co(TPP)	(5,10,15,20-tetraphenylporphyinato)cobalt(II)
CuAAC	copper(I)-catalyzed alkyne-azide cycloaddition
CuPPh	[5,10,15,20-tetrakis(4-aminophenyl)porphyrinato]copper(II)
DABCO	1,4-diazabicyclo[2.2.2]octane
DCC	*N*,*N’*-dicyclohexylcarbodiimide
DIPEA	*N*,*N-*Diisopropylethylamine
DMAP	4-(dimethylamino)pyridine
DMF	*N*,*N*-dimethylformamide
DMSO	Dimethyl Sulfoxide
DNA	Deoxyribonucleic Acid
DSSC	Dye-Sensitized Solar Cell
E. coli	Escherichia coli
EDC	1-Ethyl-3-(3-dimethylaminopropyl)carbodiimide
FeTSPP	[5,10,15,20-tetrakis-(4-sulfonatophenyl)porphyrinato]iron(III) chloride
FRET	Förster Resonance Energy Transfer
GCE	Glassy Carbon Electrodes
H_2_TPPP	5,10,15,20-tetrakis(4-(3-(pyrazin-2-yl)-1*H*-pyrazo-1-yl)phenyl)porphyrin
HOBt	1-hydroxybenzotriazole
hPG	Hyperbranched Polyglycerol
IME	1,3-dimethylimidazol-2-ylidene
ITO	Indium Tin Oxide
LbL	Layer-by-Layer
LNCaP	Prostate Carcinoma Cell Line
MCF7	Breast Cancer Cell Line
MiaPaCa-2	Pancreatic Carcinoma Cell Line
MOF	Metal–Organic Framework
mPEG	methoxypoly(ethylene glycol)
MRI	Magnetic Resonance Imaging
MW	Microwave Irradiation
PA-1	Human Ovarian Teratocarcinoma Cell
Pc	Phthalocyanine
PDI	Photodynamic Inactivation of Microorganisms
PDT	Photodynamic Therapy
PEG	Polyethylene Glycol
PET	Photoinduced Electron Transfer
PET	Positron Emission Tomography
PMEDTA	*N*,*N´*,*N′*,*N″*,*N″*-pentamethyl-diethylenetriamine
POSS	Polyhedral Oligomeric Silsesquioxane
rt	Room Temperature
Si-PAMAM	siloxane-poly(amido amine)
TBAF	Tetrabutylammonium fFuoride
TDCPP	5,10,15,20-tetrakis(2,6-dichlorophenyl)porphyrin
TFA	Trifluoroacetic Acid
THF	Tetrahydrofuran
TIPS	Triisopropylsilyl Ether
TPP	5,10,15,20-tetraphenylporphyrin
U-2 OS	Osteosarcoma Cell Line
UV	Ultraviolet
Vis	Visible
